# Enantioselective Reductive Alkenylation of α‐CF_2_H (‐CF_3_) Amino Halides: Rapid Access to Chiral α‐CF_2_H (‐CF_3_) Allylamines

**DOI:** 10.1002/advs.73986

**Published:** 2026-01-28

**Authors:** Peng Liu, Kuiliang Li, Yan He, Chen‐Hui Jiang, Ruo‐Xing Jin, Duo‐Duo Hu, Wei‐Cheng Zhao, Xuan Nie, Shang‐Zheng Sun, Xi‐Sheng Wang

**Affiliations:** ^1^ Department of Pharmacy, Division of Life Sciences and Medicine, The First Affiliated Hospital of USTC University of Science and Technology of China Hefei China; ^2^ Department of Chemistry University of Science and Technology of China Hefei China; ^3^ School of Chemical and Blasting Engineering Anhui University of Science and Technology Huainan China

**Keywords:** α‐CF_2_H amines, CF_2_H synthons, enantioselective, nickel catalysis, reductive coupling

## Abstract

The incorporation of fluoroalkyl groups into drug candidates has garnered increasing attention in the pharmaceutical industry due to their ability to modulate lipophilicity, permeability, metabolic stability, and binding affinity. Despite significant advances realized, the means to introducing fluoroalkyl groups such as ─CF_2_H or ─CF_3_ in an enantioselective manner remain scarce. Herein, we report a Ni‐catalyzed enantioselective reductive alkenylation of α‐CF_2_H or ─CF_3_ amino chlorides with vinyl iodides. This method provides an efficient and modular technique for constructing stereocenters bearing a fluoroalkyl group. A key to success was the incorporation of an arylamide moiety with the substrates, which stabilizes the α‐fluoroalkyl radical intermediate, thus offering a *de novo* approach to access enantioenriched α‐CF_2_H (─CF_3_) allylamines. Our protocol is characterized by its mild reaction conditions, broad substrate scope, as well as excellent enantio—and chemo‐selectivity, even in the context of late‐stage functionalization.

## Graphical abstract

Difluoromethyl (CF_2_H) group, the bioisostere of hydroxyl thiol methyl or amide is privileged in medicinal chemistry due to hydrogen‐binding ability and lipophilic, metabolically stable, and chemical inert nature. However, enatioseleceive CF_2_H introduction remains challenging. We report a modular approach for the constructing stereocenters bearing CF_2_H or CF_3_ groups via Ni‐catalyzed asymmetric reductive alkenylation of α‐CF_2_H or ‐CF_3_ amino chlorides with vinyl iodides.

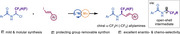

## 1 Introduction

**SCHEME 1:**
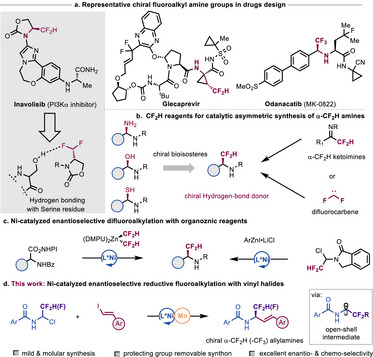
The synthesis of chiral α‐fluoroalkyl amine moieties.

In modern drug development, the optimization of physicochemical and ADME (absorption, distribution, metabolism, excretion) properties of lead compounds and drug‐like molecules plays a crucial role [1, 2]. Among various strategies, the bioisosteric replacement is a widely employed approach as it often leads to drug candidates with enhanced potency [3]. Driven by this, chemists have recently been challenged to design general and efficient techniques for the synthesis of bioisosteric analogues. The incorporation of fluoroalkyl moieties into drug molecules can confer significant benefits, as they can modulate lipophilicity, membrane permeability, metabolic stability, and enhance protein‐drug interactions [4, 5, 6, 7, 8, 9]. In particular, difluoromethyl (CF_2_H) group, the bioisostere of hydroxyl (‐OH), thiol (‐SH), methyl (‐Me) or amide group has emerged as privileged group in medicinal chemistry due to its ability to participate hydrogen‐binding interactions as well as its lipophilic, metabolically stable and chemical inert nature (Scheme 1a,b) [10, 11, 12, 13]. Indeed, a significant increase of CF_2_H containing lead compounds have been developed, including FDA‐ approved drugs in recent years (Scheme 1a) [12].

Although advanced techniques for accessing enantioenriched *sp^3^* C−CF_3_ architectures [14, 15, 16, 17, 18, 19], the introducing CF_2_H group into organic molecules in an enantioselective manner ‐even in the context of late‐stages—remains rare [20, 21]. This paucity is likely due to the limited availability of CF_2_H regents or precursors. Driven by the prevalence of alkyl amine in pharmaceuticals and preclinical candidates, the design of a general approach for constructing enantioenriched α‐CF_2_H centers in alkyl amines has gained increasing interest and would offer significant advantages in organic synthesis and medicinal chemistry [22, 23, 24]. While reductive couplings of imines are established for alkyl amines synthesis, the synthesis of fluoroalkyl‐substituted alkyl amines by this strategy has received far less attention [25, 26]. At present, existing methods for constructing chiral α‐CF_2_H amines are based on nucleophilic addition with α‐CF_2_H ketoimines [27, 28, 29] or difluorocarbene (Scheme 1b) [30]. However, these approaches either have limited scope, moderate enantioselectivity, or require using difluoromonochloromethane—an environmentally unfriendly gas—as a precursor. Very recently, Liu and co‐workers disclosed an enantioselective Ni‐catalyzed decarboxylative difluoromethylation of α‐amino acid derived redox esters with difluoromethyl zinc reagent (Scheme 1c, left) [31]. Simultaneously, our group developed an asymmetric difluoroalkylation of isoindolinone protected α‐CF_2_H synthons with arylzinc reagents (Scheme 1c, right) [32]. These methods, while efficient for the preparation of chiral α‐CF_2_H amines, suffer from the utilization of air‐ and moisture‐sensitive organometallics as well as the requirement of a multistep deprotection for the latter.

To address these challenges, we wondered whether an enantioselective difluoroalkylation could be implemented using easily available starting materials via asymmetric reductive coupling techniques [33, 34, 35]. If successful, this would offer a new strategic approach for the rapid construction of chiral α‐CF_2_H amines [36]. In our continuing interest in nickel‐catalyzed reductive couplings, we report herein the successful realization of this goal (Scheme 1d) [37, 38, 39]. The utilization of new CF_2_H and CF_3_ synthons offers an efficient technique for the synthesis of enantioenriched α‐CF_2_H or ‐CF_3_ allylamines in a modular fashion. This protocol is distinguished by its mild conditions, broad substrate scope, and excellent enantio‐ & chemo‐selectivity. Mechanistic studies reveal that an α‐amino‐stabilized fluoroalkyl radical intermediate participates in the stereoselective C─C bond formation. This technique highlights the advantage of using fluoroalkyl synthons for the enantioselective functionalization of fluoroalkyl open‐shell intermediates.

## 2 Result and Discussion

We began our investigation by evaluating the enantioselective reductive coupling using *N*‐(1‐chloro‐2,2‐difluoroethyl)benzamide (**1a**) as the CF_2_H‐containing coupling partner with methyl (*E*)‐4‐(2‐iodovinyl)benzoate (**2a**) (**Table** **1**). Careful evaluation of all reaction parameters revealed the critical role exerted by the nature and substitution pattern of the ligand backbone. Indeed, the utilization of bis‐oxazoline ligand (**L1**), or box‐type ligand **L2**‐**5** gave the desired product in low yields and enantiomeric excess (*ee*). To our delight, the 4,5‐diphenyl substituted bis(oxazoline) ligands provided promising results, with the benzyl group on the tether (**L7**) improved the *ee* value to 55%. Gratifyingly, the utilization of a more sterically encumbered group instead of a benzyl group on the tether led to a significant increase in both yield and enantioselectivity (**L8**, **L9**), with **L9** providing **3a** in 66% yield and 90% *ee*. However, the inclusion of di‐phenyl groups at the C5 position of bis(oxazoline) ligands (**L10**‐**L12**) significantly inhibited the reactivity, resulting in only trace amount product. Gratifyingly, employing TMSCl instead of TBAI as additives led to comparable yield and superior enantioselectivity (Table 1, entries 7–8). Moreover, the solvent choice was also crucial for success, as replacing THF with other common solvents drastically decreased both reactivity and enantioselectivity (Table 1, entries 1–4). As anticipated, extending the reaction time to 48 h, the desired product was obtained in 87% yield without erosion of the enantioselectivity (Table 1, entry 11). Importantly, nickel sources, reductant, additives, and solvents other than NiBr_2_· DME, Mn (2.0 equiv), TMSCl (0.8 equiv), and THF resulted in either lower yields or inferior *ee* (Table 1, entries 1–10). More details on the additional optimization of reaction conditions can be found in Tables S1–S7.

**TABLE 1:** Optimization of reaction conditions.

Entry	Ni cat.	solvent	additive	yield (%)^b^	ee (%)^c^
1	NiBr_2_· DME	DME	TBAI	54	87
2	NiBr_2_· DME	2‐Me THF	TBAI	41	89
3	NiBr_2_· DME	Diglyme	TBAI	64	81
4	NiBr_2_· DME	DMF	TBAI	10	59
5	NiBr_2_· DME	THF	NaI	63	86
6	NiBr_2_· DME	THF	KI	57	88
7	NiBr_2_· DME	THF	TMSCl	60	97
8 ^d^	NiBr_2_· DME	THF	TMSCl	65	97
9 ^d^	NiCl_2_· DME	THF	TMSCl	41	92
10 ^d^	NiBr_2_	THF	TMSCl	N.D.	—
11 ^d, e^	NiBr_2_· DME	THF	TMSCl	87 (82) ^f^	98

^a^ Unless otherwise noted, all reactions were performed with **1a** (0.12 mmol, 1.2 equiv), **2a** (0.1 mmol, 1.0 equiv), Ni cat. (0.01 mmol, 10 mol%), chiral ligand **L** (0.013 mmol, 13 mol%), Mn (2.0 equiv), additive (0.5 equiv), THF (1 mL), under Ar, at ‐20°C for 24 h.

^b^ Yields were determined by ^1^H NMR analysis of crude mixture with CH_2_Br_2_ as internal standard.

^c^ Enantiomeric excess (*ee*) values were determined by HPLC on a chiral stationary phase.

^d^ TMSCl (0.8 equiv) was used.

^e^ 48 h.

^f^ Isolated yield.

**SCHEME 2:**
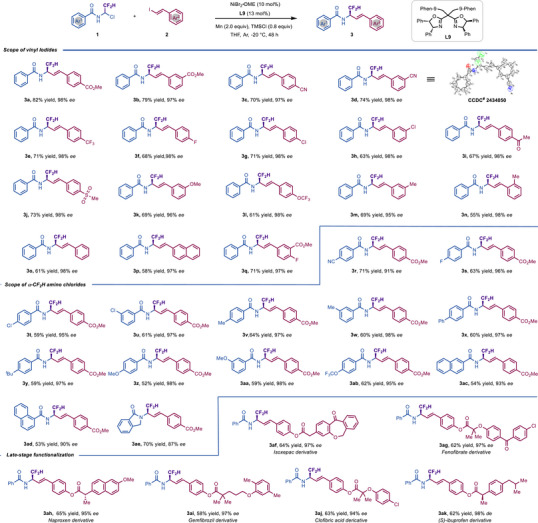
Scope of enantioselective reductive alkenylation of α‐CF_2_H amino chlorides with vinyl iodides.^a^Reaction conditions were as follows: **1** (0.24 mmol, 1.2 equiv), **2** (0.2 mmol, 1.0 equiv), NiBr_2_·DME (0.02 mmol, 10 mol%), **L9** (0.026 mmol, 13 mol%), TMSCl (0.16 mmol, 0.8 equiv), Mn (2.0 equiv), THF (2.0 mL), Ar, −20°C. Isolated yields. The *ee* values were determined by HPLC analysis on a chiral stationary phase.

Encouraged by these initial findings, we turned our attention to studying the generality of our enantioselective reductive alkenylation of α‐CF_2_H amino chlorides with vinyl iodides. As shown in Scheme 2, a diverse array of aryl‐conjugated vinyl iodides with *ortho‐*, *meta‐*, and *para‐* substituents, regardless of whether electron‐donating or electron‐withdrawing groups on the aromatic ring, were efficiently converted into desired products in moderate to good yields and with excellent enantioselective control (**3a**‐**3q**). The absolute stereochemistry of the products was unequivocally confirmed by X‐ray crystallographic analysis of **3d** [40]. The chemoselectivity of our reaction is illustrated by the compatibility in the presence of ester (**3a**, **3b**, **3q**), cyano (**3c**, **3d**), ketone (**3i**), sulfone (**3j**), among others. Notably, aryl halides (**3f**, **3** **g**, **3** **h**) could all be well accommodated, thus providing ample opportunities for further derivatization via conventional cross‐coupling reactions. Next, we evaluated the influence the α‐CF_2_H amino chlorides precursors on the catalytic asymmetric alkenylation events. Gratifyingly, the substituents on the phenyl ring of the aryl amides were largely inconsequential to the reactivity and stereoselectivity profile (**3r**–**3ad**). Importantly, the *N,N*‐disubstituted substrate such as 2‐(1‐chloro‐2,2‐difluoroethyl) isoindolin‐1‐one underwent targeted reaction in good yield with only slightly low enantioselectivity (70% yield, 87% *ee*), indicating that the steric effect of the protecting group might not compromise the reactivity or stereoselectivity (**3ae**). Motivated by the favorable functional group compatibility demonstrated in our reactions, we set out to explore the potential application of this transformation in the late‐stage diversification of biologically active molecules. Accordingly, we subjected a series of “drug‐like” vinyl iodides to this asymmetric alkenylation protocol. We were pleased to find that the vinyl halides derived from isoxepac (**3af**), fenofibrate (**3ag**), naproxen (**3ah**), gemfibrozil (**3al**), clofibric (**3aj**), and (*S*)‐ibuprofen (**3ak**) were perfectly accommodated, furnishing the chiral α‐CF_2_H allylamines in good yield and excellent enantioselectivity (up to 98% *ee*).

**SCHEME 3:**
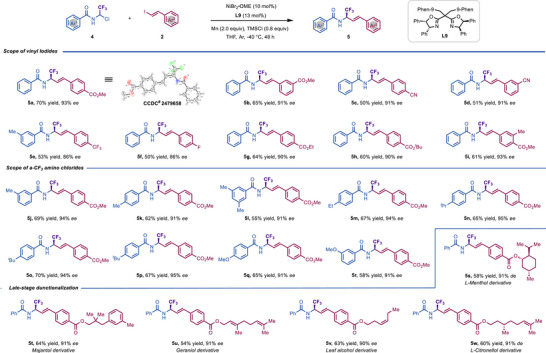
Scope of enantioselective reductive alkenylation of α‐CF_3_ amino chlorides with vinyl iodides.^a^Reaction conditions were as follows: **4** (0.28 mmol, 1.4 equiv), **2** (0.2 mmol, 1.0 equiv), NiBr_2_·DME (0.02 mmol, 10 mol%), **L9** (0.026 mmol, 13 mol%), TMSCl (0.16 mmol, 0.8 equiv), THF (1.2 mL), Ar, ‐40°C. Isolated yields. The *ee* values were determined by HPLC analysis on a chiral stationary phase.

Driven by the prevalence of the trifluoromethyl (‐CF_3_) group in pharmaceuticals and preclinical candidates, we wondered whether our enantioselective reductive coupling technique could be extended to α‐CF_3_ containing alkyl halides [41]. Gratifyingly, this was the case; the treatment of the reaction at −40°C was crucial for success, providing α‐CF_3_ allylamines in good yields with high levels of enantioselectivity (Scheme 3). The structure of **5a** was unambiguously confirmed by X‐ray crystallographic analysis [42]. As shown, the reaction could also accommodate ester (**5a**, **5b**, **5g**‐**5i**), cyano (**5c**, **5d**), trifluoromethyl (**5e**), aryl halides (**5f**), among others. However, this reaction presents poor compatibility with electron‐deficient vinyl iodides due to their slow oxidative addition ability with nickel species. As expected, the target enantioselective *sp^3^*−*sp^2^* bond formation could be extended to other aryl carboxylic acids protected α‐CF_3_ amines (**5j**‐**5r**). The applicability of this transformation is further illustrated in the preparation of derivatives from (*L*)‐menthol (**5s**), majantol (**5t**), geraniol (**5u**), leaf alcohol (**5v**), and (*L*)‐citronellol (**5w**).

**SCHEME 4:**
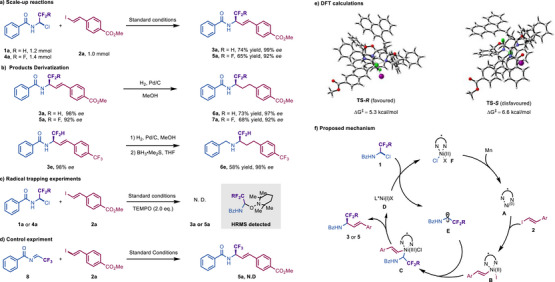
Synthetic application and mechanistic studies. (a) Scale‐up reactions. (b) Products derivatization. (c) Radical trapping experiment. (d) Control experiments. (e) DFT calculations. (f) Proposed mechanism.

Furthermore, the results shown in Scheme 4 illustrate the synthetic value of our protocol. As shown in Scheme 4a, our reaction could be scaled up without an erosion in yield and enantioselectivity under standard conditions. The corresponding products **3a** and **5a** were obtained with 74% and 65% yield, accompanied by an excellent *ee* value (99% *ee* and 92% *ee*), respectively. Moreover, the unsaturated double bond could be directly hydrogenation under mild conditions, furnishing chiral fluoroalkyl amides (**6a** and **7a**) in good yield and enantioselectivity. In addition, the successful preparation of **6e** from **3e** was achieved by a sequence of simple Pd/C reduction followed by BH_3_ reduction, further illustrating the potential that our technique might have for accessing chiral fluoroalkyl amines with a high level of enantioselectivity (Scheme 4b).

Next, we conducted preliminary experiments to gather indirect evidence about the mechanism of our asymmetric reductive coupling reactions. As shown in Scheme 4c, when the reaction was conducted in the presence of 2.0 equivalents of TEMPO, the formation of the desired product was entirely suppressed. Additionally, HRMS analysis of the crude reaction mixture revealed the formation of TEMPO adducts, suggesting the reaction mechanism involves a SET step leading to the generation of α‐CF_2_R amino radical intermediate. We also considered the possibility of the involvement of α‐CF_2_R imine intermediate [43]. To evaluate this scenario, α‐CF_3_ imine **8** was subjected to the standard conditions. The desired product **5a** was not detected in the crude mixture, thereby demonstrating that α‐CF_3_ imines are unlikely intermediates. To further understand the origin of the enantioselectivity, we performed density functional theory (DFT) calculations. As shown, with **L9** as the chiral ligand, the transition state **TS‐*R***, which leads to the formation of **Pro‐*R***, proceeds with a lower activation barrier (∆G^‡^ = 5.3 kcal/mol) compared with its counterpart, **TS‐*S*** (∆G^‡^ = 6.6 kcal/mol), indicating that the ***R*** configuration of two enantiomers was favored under ligand regulation (Scheme 4e). Based on the experimental results and precedents in asymmetric nickel catalysis, we postulate a reaction mechanism as outlined in Scheme 4f [14, 17, 34]. The catalytic cycle is initiated by oxidative addition of substrate **2** to the Ni(0) center coordinated by the chiral ligand *L9, forming Ni(II)/*L9 complex **B**, and followed by recombination with the open‐shell intermediate **E**. Reductive elimination from **C** delivers enantioenriched product and Ni(I)/*L9 (**D**), which triggers a SET to **1**, leading to Ni(II)/*L9 (**F**) that ultimately regenerates the propagating Ni(0)/*L9 (**A**) with Mn.^44^

## 3 Conclusion

In summary, we have developed an efficient and versatile Ni‐catalyzed asymmetric reductive alkenylation of α‐CF_2_H (‐CF_3_) amine halides with vinyl halides, enabling the rapid synthesis of a wide range of enantioenriched α‐CF_2_H and α‐CF_3_ containing allylamines. The method is characterized by its mild conditions, broad substrate scope, and excellent enantio‐ & chemo‐selectivity, even in the context of late‐stage functionalization. This technique highlights the benefit of using fluoroalkyl containing synthons for the enantioselective functionalization of fluoroalkyl open‐shell intermediates. We anticipate that this method will be valuable to the medicinal chemistry community and will accelerate the discovery of drug candidates containing CF_2_H and other fluoroalkyl stereocenters.

## Conflicts of Interest

The authors declare no conflict of interest.

## Supporting information




**Supporting File**: advs73986‐sup‐0001‐SuppMat.docx.

## Data Availability

The data that support the findings of this study are available in the Supporting Information of this article.
