# Supplementary material for: Enantioselective Reductive Alkenylation of α‐CF2H (‐CF3) Amino Halides: Rapid Access to Chiral α‐CF2H (‐CF3) Allylamines
Source: Adv Sci (Weinh). 2026 Jan 28;13(18):e73986. doi: 10.1002/advs.73986 (PMC13042639; doi:10.1002/advs.73986)
Supplement: Supplementary file 1 — Supporting File: advs73986‐sup‐0001‐SuppMat.docx. [file ADVS-13-e73986-s001.docx]

**Supplementary Information**

**for**

**Enantioselective Reductive Alkenylation of α-CF_2_H (-CF_3_) Amino Halides: Rapid Access to Chiral α-CF_2_H (-CF_3_) Allylamines**

Peng Liu^+^, Kuiliang Li^+^, Yan He^+^, Chen-Hui Jiang, Ruo-Xing Jin, Duoduo Hu, Wei-Cheng Zhao, Xuan Nie*, Shang-Zheng Sun* and Xi-Sheng Wang*

[a] Peng Liu, Xuan Nie, Xi-Sheng Wang

Department of Pharmacy, The First Affiliated Hospital of USTC, Division of Life Sciences and Medicine, University of Science and Technology of China, Hefei 230026, China.

E-mail: niexuan@ustc.edu.cn; xswang77@ustc.edu.cn

[b] Peng Liu, Yan He, Chen-Hui Jiang, Ruo-Xing Jin, Wei-Cheng Zhao, Shang-Zheng Sun, Xi-Sheng Wang.
Department of Chemistry, University of Science and Technology of China, Hefei 230026, China.
E-mail: ssun123@ustc.edu.cn

[c] Kuiliang Li, Duoduo Hu
School of Chemical and Blasting Engineering, Anhui University of Science and Technology, Huainan 232001, China.

^+^	These authors contributed equally to this article.

Supporting information for this article is given via a link at the end of the document.

**Tabel of Contents**

[General Information	2](#_Toc218539995)

[Optimization of conditions	3](#_Toc218539996)

[General procedures	7](#_Toc218539997)

[General procedure A for the synthesis of vinyl iodides	7](#_Toc218539998)

[General procedure B for the synthesis of the substrates 1a-1n, 4a-4j:	7](#_Toc218539999)

[General Procedure C1 for Enantioselective Reductive Alkenylation of *α*-CF_2_H Amino Halides	16](#_Toc218540000)

[General Procedure C2 for Enantioselective Reductive Alkenylation of *α*-CF_3_ Amino Halides	16](#_Toc218540001)

[Scale-up reactions	42](#_Toc218540002)

Products [Derivatization	43](#_Toc218540003)

[Mechanism Investigation	46](#_Toc218540004)

[Computational Details	48](#_Toc218540005)

[NMR Spectra of Products (^1^H NMR, ^19^F NMR, ^13^C NMR)	69](#_Toc218540006)

[HPLC Data of Products	200](#_Toc218540007)

[Unsuccessful examples	263](#_Toc218540008)

[X-ray Crystallographic Data of 3d and 5a	264](#_Toc218540009)

[References	266](#_Toc218540010)

General Information

NMR spectra were recorded on Bruker-400 MHz NMR spectrometer (400 MHz for ^1^H; 101 MHz for ^13^C and 376 MHz for ^19^F) or Bruker 500 MHz spectrometer (500 MHz for ^1^H; 126 MHz for ^13^C and 471 MHz for ^19^F) or Bruker 600 MHz spectrometer (600 MHz for ^1^H; 151 MHz for ^13^C and 565 MHz for ^19^F). ^1^H NMR chemical shifts were determined relative to internal (CH_3_)_4_Si at δ 0.0 or at the signal of a residual protonated solvent: CDCl_3_ δ 7.26. ^13^C NMR chemical shifts were determined relative to CDCl_3_ δ 77.16. ^19^F NMR chemical shifts were determined relative to CFCl_3_ at δ 0.0. Data for ^1^H, ^13^C, ^19^F NMR are recorded as follows: chemical shift (δ, ppm), multiplicity (s = singlet, d = doublet, t = triplet, m = multiplet, q = quartet, br = broad, et al.). High resolution mass spectra were recorded on P-SIMS-Gly of Bruker Daltonics Inc. using ESI-TOF (electrospray ionization-time of flight) or Micromass GCT using EI (electron impact). Unless otherwise noted, all other reagents and starting materials were purchased from commercial sources and used without further purification. NiBr_2_·DME was obtained from Strem Chemicals. Mn podwer was purchased from Adamas. TMSCl wsa purchased from Aladdin. Anhydrous THF was purchased from J&K Chemicals. CF_2_H/CF_3_-substitued alkyl chloride and vinyl iodides were synthesized via following method described in this supplementary information.

Optimization of conditions

**Table S1.** Chiral ligands screening^a^

**Table S2.** Solvent screening^a^

**Table S3.** Additive screening^a^

**Table S4.** The amount of TMSCl screening

**Table S5.** Ni catalyst screening^a^

**Table S6.** Temperature screening^a^

**Table S7.** Reaction time screening^a^

General procedures

General procedure A for the synthesis of vinyl iodides

General Procedure A^1^ : The vinyl iodides was synthesized according to the reported literature.^[1][2]^

General procedure B for the synthesis of the substrates 1a-1n, 4a-4j:

To an oven*-*dried 25 mL Schlenk tube with Teflon-coated stirrer bar was added benzamide compound (3 mmol, 1.0 equiv.) and 4Å MS (1g) under air. The tube was evacuated and backfilled with Ar (three times), then anhydrous dioxane (6 mL) and 1-ethoxy-2,2*-d*ifluoroethan-1-ol or 1-ethoxy-2,2,2-trifluoroethan-1-ol (3.6 mmol, 1.2equiv.) was added. The tube was sealed and the reaction was stirred vigorously at 101 ℃ for 72 h. After evaporation of the solvent, the residue was purified by flash column chromatography to afford the desired products **S1a-S1n, S4a-S4j** and SOCl_2_ (2.5 equiv) were dissolved in CH_2_Cl_2_ (5 mL) into a 10 mL tube. The reaction was stirred at room tempature for 24 h. Then, the reaction mixture was evaporated in vacuo to give the desired compound **1a**-**1n**, **4a**-**4j** without furthure purification.

**N-(1-chloro-2,2-difluoroethyl)benzamide (1a)**

 Prepared following general procedure B as a white solid (394.2 mg, 60% yield for two steps). ^1^H NMR (400 MHz, Chloroform*-d*) δ 7.82 (d, *J =* 7.5 Hz, 2H), 7.59 (t, *J =* 7.3 Hz, 1H), 7.48 (t, *J =* 7.5 Hz, 2H), 7.04 (d, *J =* 10.3 Hz, 1H), 6.48 – 6.26 (m, 1H), 6.06 (t, *J =* 54.9 Hz, 1H) ppm; ^13^C NMR (101 MHz, Chloroform*-d*) δ 166.85, 133.09, 132.25, 129.02, 127.56, 112.48 (t, *J =* 248.3 Hz), 63.05 (dd, *J =* 27.6, 24.3 Hz) ppm; ^19^F NMR (376 MHz, Chloroform*-d*) δ -121.26 (ddd, *J =* 281.1, 54.8, 8.5 Hz), -131.35 (ddd, *J =* 281.3, 54.8, 13.6 Hz) ppm. HRMS (ESI) (*m/z*): [M+Na]^+^ calcd. for C_9_H_8_ClF_2_NNaO: 242.0155, found: 242.0164.

**N-(1-chloro-2,2-difluoroethyl)-4-cyanobenzamide (1b)**

 Prepared following general procedure B as a white solid (256.2 mg, 35% yield for two steps). ^1^H NMR (400 MHz, Chloroform*-d*) δ 7.94 (d, *J =* 8.4 Hz, 2H), 7.80 (d, *J =* 8.3 Hz, 2H), 6.99 (d, *J =* 10.5 Hz, 1H), 6.35 (dddd, *J =* 14.5, 10.0, 8.0, 1.5 Hz, 1H), 6.07 (td, *J =* 54.8, 1.6 Hz, 1H) ppm. ^13^C NMR (101 MHz, Chloroform*-d*) δ 165.16, 136.10, 132.87, 128.29, 117.73, 116.62, 112.36 (t, *J =* 248.5 Hz), 62.59 (dd, *J =* 28.0, 23.5 Hz) ppm. ^19^F NMR (376 MHz, Chloroform*-d*) δ -120.48 (ddd, *J =* 281.7, 55.1, 8.7 Hz), -132.30 (ddd, *J =* 281.8, 55.0, 15.0 Hz) ppm. HRMS (ESI) (*m/z*): [M+H]^+^ calcd. for C_10_H_8_ClF_2_N_2_O: 245.0288, found: 245.0287.

**N-(1-chloro-2,2-difluoroethyl)-4-fluorobenzamide (1c)**

Prepared following general procedure B as a white solid (404.0 mg, 52% yield for two steps). ^1^H NMR (400 MHz, Chloroform*-d*) δ 8.00 – 7.70 (m, 2H), 7.16 (t, *J =* 8.5 Hz, 2H), 6.94 (d, *J =* 10.4 Hz, 1H), 6.36 (dddd, *J =* 14.0, 10.3, 8.4, 1.6 Hz, 1H), 6.05 (td, *J =* 54.9, 1.6 Hz, 1H) ppm. ^13^C NMR (101 MHz, Chloroform*-d*) δ165.75, 165.69 (d, *J =* 254.6 Hz), 130.13 (d, *J =* 9.3 Hz), 128.50, 116.24 (d, *J =* 22.1 Hz), 112.46 (t, *J =* 248.5 Hz), 63.00 (dd, *J =* 28.0, 24.0 Hz) ppm. ^19^F NMR (376 MHz, Chloroform*-d*) δ -105.16 (td, *J =* 8.8, 4.7 Hz), -120.99 (ddd, *J =* 281.6, 55.2, 9.4 Hz), -131.76 (ddd, *J =* 281.5, 55.2, 14.7 Hz) ppm. HRMS (ESI) (*m/z*): [M+Na]^+^ calcd. for C_9_H_7_ClF_3_NNaO: 260.0060, found: 260.0087.

**4-chloro-N-(1-chloro-2,2-difluoroethyl) benzamide (1d)**

 Prepared following general procedure B as a white solid (438.5 mg, 58% yield for two steps). 1H NMR (400 MHz, Chloroform*-d*) δ 8.17 – 7.65 (m, 2H), 7.46 (d, *J =* 8.6 Hz, 2H), 6.93 (d, *J =* 10.8 Hz, 1H), 6.35 (dddd, *J =* 14.1, 10.3, 8.4, 1.6 Hz, 1H), 6.05 (td, *J =* 54.9, 1.6 Hz, 1H) ppm. ^13^C NMR (101 MHz, Chloroform*-d*) δ 165.84, 139.58, 130.65, 129.37, 129.00, 112.43 (t, *J =* 248.5 Hz), 62.90 (dd, *J =* 27.9, 23.9 Hz) ppm. ^19^F NMR (376 MHz, Chloroform*-d*) δ -120.91 (ddd, *J =* 281.5, 55.2, 9.0 Hz), -131.85 (ddd, *J =* 281.6, 55.1, 14.7 Hz) ppm. HRMS (ESI) (*m/z*): [M+H]^+^ calcd. for C_9_H_8_Cl_2_F_2_NO: 253.9946, found: 253.9953.

**3-chloro-N-(1-chloro-2,2-difluoroethyl)benzamide (1e)**

 Prepared following general procedure B as a white solid (461.2 mg, 61% yield for two steps). ^1^H NMR (400 MHz, Chloroform*-d*) δ 7.80 (t, *J =* 1.9 Hz, 1H), 7.68 (dt, *J =* 7.8, 1.4 Hz, 1H), 7.57 – 7.52 (m, 1H), 7.43 (t, *J =* 7.9 Hz, 1H), 6.95 (d, *J =* 10.6 Hz, 1H), 6.35 (dddd, *J =* 14.0, 10.3, 8.3, 1.6 Hz, 1H), 6.05 (td, *J =* 54.8, 1.6 Hz, 1H) ppm. ^13^C NMR (101 MHz, Chloroform*-d*) δ 165.61, 135.33, 134.04, 133.13, 130.38, 127.93, 125.57, 112.41 (t, *J =* 248.5 Hz), 62.81 (dd, *J =* 28.0, 24.0 Hz) ppm. ^19^F NMR (376 MHz, Chloroform*-d*) δ -120.94 (ddd, *J =* 281.6, 55.2, 9.2 Hz), -131.80 (ddd, *J =* 281.4, 55.1, 14.5 Hz) ppm. HRMS (ESI) (*m/z*): [M+H]^+^ calcd. for C_9_H_8_Cl_2_F_2_NO: 253.9946, found: 253.9932.

**N-(1-chloro-2,2-difluoroethyl)-4-methylbenzamide (1f)**

 Prepared following general procedure B as a white solid (335.5 mg, 48% yield for two steps). ^1^H NMR (400 MHz, Chloroform*-d*) δ 7.72 (d, *J =* 7.9 Hz, 2H), 7.28 (d, *J =* 7.7 Hz, 2H), 6.93 (d, *J =* 10.4 Hz, 1H), 6.39 (dt, *J =* 13.6, 9.7 Hz, 1H), 6.05 (t, *J =* 54.9 Hz, 1H) ppm. ^13^C NMR (101 MHz, Chloroform*-d*) δ 166.68, 143.90, 129.68, 129.41, 127.58, 112.51 (t, *J =* 248.2 Hz), 63.16 (dd, *J =* 27.6, 24.1 Hz). 21.73 ppm. ^19^F NMR (376 MHz, Chloroform*-d*) δ -121.31 (ddd, *J =* 281.2, 55.4, 9.8 Hz), -131.40 (ddd, *J =* 281.3, 55.2, 14.3 Hz) ppm. HRMS (ESI) (*m/z*): [M+H]^+^ calcd. for C_10_H_11_ClF_2_NO: 234.0492, found: 234.0490.

**N-(1-chloro-2,2-difluoroethyl)-4-methylbenzamide (1g)**

 Prepared following general procedure B as a white solid (377.5 mg, 54% yield for two steps. ^1^H NMR (400 MHz, Chloroform*-d*) δ 7.69 – 7.55 (m, 2H), 7.45 – 7.32 (m, 2H), 6.97 (d, *J =* 10.5 Hz, 1H), 6.38 (dt, *J =* 13.5, 9.7 Hz, 1H), 6.05 (td, *J =* 54.9, 1.5 Hz, 1H), 2.41 (s, 3H) ppm. ^13^C NMR (101 MHz, Chloroform*-d*) δ 167.00, 139.03, 133.85, 132.25, 128.90, 128.22, 124.53, 112.49 (t, *J =* 248.3 Hz), 63.07 (dd, *J =* 27.5, 24.4 Hz), 21.45 ppm. ^19^F NMR (376 MHz, Chloroform*-d*) δ -121.28 (ddd, *J =* 281.4, 55.3, 9.7 Hz), -131.39 (ddd, *J =* 281.3, 55.2, 14.3 Hz) ppm. HRMS (ESI) (*m/z*): [M+Na]^+^ calcd. for C_10_H_10_ClF_2_NNaO: 256.0311, found: 256.0304.

**N-(1-chloro-2,2-difluoroethyl)-[1,1'-biphenyl]-4-carboxamide (1h)**

 Prepared following general procedure B as a white solid (389.5 mg, 44% yield for two steps). ^1^H NMR (400 MHz, Chloroform*-d*) δ 7.90 (d, *J =* 7.8 Hz, 2H), 7.69 (d, *J =* 7.8 Hz, 2H), 7.61 (d, *J =* 7.5 Hz, 2H), 7.48 (t, *J =* 7.3 Hz, 2H), 7.42 (d, *J =* 7.1 Hz, 1H), 7.09 (d, *J =* 10.2 Hz, 1H), 6.42 (dt, *J =* 13.6, 9.4 Hz, 1H), 6.08 (t, *J =* 54.9 Hz, 1H) ppm. ^13^C NMR (101 MHz, Chloroform*-d*) δ 166.60, 145.88, 139.62, 130.80, 129.13, 128.48, 128.15, 127.61, 127.36, 112.51 (t, *J =* 248.2 Hz), 63.13 (dd, *J =* 27.7, 24.1 Hz) ppm. ^19^F NMR (376 MHz, Chloroform*-d*) δ -121.20 (ddd, *J =* 281.3, 55.3, 9.9 Hz), -131.26 (ddd, *J =* 281.3, 55.6, 14.7 Hz) ppm. HRMS (ESI) (*m/z*): [M+H]^+^ calcd. for C_15_H_13_ClF_2_NO: 296.0648, found: 296.0657.

**4-(tert-butyl)-N-(1-chloro-2,2-difluoroethyl)benzamide (1i)**

 Prepared following general procedure B as a white solid (313.5 mg, 38% yield for two steps). ^1^H NMR (400 MHz, Chloroform*-d*) δ 7.76 (d, *J =* 8.5 Hz, 2H), 7.49 (d, *J =* 8.4 Hz, 2H), 6.92 (d, *J =* 10.7 Hz, 1H), 6.39 (dddd, *J =* 13.6, 10.5, 8.8, 1.6 Hz, 1H), 6.05 (td, *J =* 54.9, 1.6 Hz, 1H), 1.34 (s, 9H) ppm. ^13^C NMR (101 MHz, Chloroform*-d*) δ 166.64, 156.92, 129.42, 127.45, 126.01, 112.40 (t, *J =* 248.3 Hz), 63.02 (dd, *J =* 27.6, 24.1 Hz), 35.24, 31.21 ppm. ^19^F NMR (376 MHz, Chloroform*-d*) δ -121.36 (ddd, *J =* 281.3, 55.4, 9.7 Hz), -131.47 (ddd, *J =* 281.4, 55.2, 14.3 Hz) ppm. HRMS (ESI) (*m/z*): [M+Na]^+^ calcd. for C_13_H_16_ClF_2_NNaO: 298.0781, found: 298.0789.

**N-(1-chloro-2,2-difluoroethyl)-4-methoxybenzamide (1j)**

 Prepared following general procedure B as a white solid (455.7 mg, 61% yield for two steps). ^1^H NMR (400 MHz, Chloroform*-d*) δ 7.79 (d, *J =* 8.9 Hz, 2H), 6.95 (d, *J =* 8.9 Hz, 2H), 6.90 (d, *J =* 11.3 Hz, 1H), 6.38 (dddd, *J =* 13.6, 10.6, 8.9, 1.7 Hz, 1H), 6.04 (td, *J =* 55.0, 1.7 Hz, 1H), 3.86 (s, 3H) ppm. ^13^C NMR (101 MHz, Chloroform*-d*) δ 166.12, 163.35, 129.50, 124.34, 114.11, 112.43 (t, *J =* 248.3 Hz), 63.23 (dd, *J =* 27.7, 24.2 Hz), 55.55 ppm. ^19^F NMR (376 MHz, Chloroform*-d*) δ -121.38 (ddd, *J =* 281.3, 55.2, 9.5 Hz), -131.24 (ddd, *J =* 281.0, 55.2, 14.1 Hz) ppm. HRMS (ESI) (*m/z*): [M+Na]^+^ calcd. for C_10_H_10_ClF_2_NNaO_2_: 272.0260, found: 272.0281.

**N-(1-chloro-2,2-difluoroethyl)-3-methoxybenzamide (1k)**

 Prepared following general procedure B as a white solid (410.8 mg, 55% yield for two steps). ^1^H NMR (400 MHz, Chloroform*-d*) δ 7.40 – 7.35 (m, 2H), 7.34 – 7.31 (m, 1H), 7.11 (ddd, *J =* 8.0, 2.7, 1.2 Hz, 1H), 6.99 (d, *J =* 10.6 Hz, 1H), 6.37 (dddd, *J =* 13.8, 10.6, 8.8, 1.7 Hz, 1H), 6.05 (td, *J =* 54.9, 1.7 Hz, 1H), 3.85 (s, 3H) ppm. ^13^C NMR (101 MHz, Chloroform*-d*) δ 166.74, 160.10, 133.70, 130.04, 119.30, 119.19, 112.87, 112.46 (t, *J =* 248.3 Hz), 63.06 (dd, *J =* 27.8, 24.2 Hz), 55.65 ppm. ^19^F NMR (376 MHz, Chloroform*-d*) δ -121.32 (ddd, *J =* 281.3, 55.0, 8.7 Hz), -131.36 (ddd, *J =* 281.3, 54.7, 13.6 Hz) ppm. HRMS (ESI) (*m/z*): [M+H]^+^ calcd. for C_11_H_11_ClF_2_NO_2_: 250.0441, found: 250.0430.

**N-(1-chloro-2,2-difluoroethyl)-4-(trifluoromethoxy)benzamide (1l)**

 Prepared following general procedure B as a white solid (390.9 mg, 43% yield for two steps). ^1^H NMR (400 MHz, Chloroform*-d*) δ 7.88 (d, *J =* 8.3 Hz, 2H), 7.30 (d, *J =* 8.2 Hz, 2H), 7.04 (d, *J =* 10.6 Hz, 1H), 6.35 (dt, *J =* 13.8, 9.3 Hz, 1H), 6.05 (t, *J =* 54.9 Hz, 1H). 120.39 (q, *J =* 258.6 Hz) ppm. ^13^C NMR (101 MHz, Chloroform*-d*) δ 165.65, 152.63, 130.66, 129.64, 120.94, 120.37 (q, *J =* 259.0 Hz), 112.44 (t, *J =* 248.5 Hz), 62.89 (dd, *J =* 28.1, 23.7 Hz) ppm. ^19^F NMR (376 MHz, Chloroform*-d*) δ -57.73, -120.95 (ddd, *J =* 281.9, 55.1, 8.6 Hz), -131.79 (ddd, *J =* 281.3, 54.6, 13.8 Hz) ppm. HRMS (ESI) (*m/z*): [M+Na]^+^ calcd. for C_10_H_7_ClF_5_NNaO_2_: 325.9978, found: 326.0004.

**N-(1-chloro-2,2-difluoroethyl)-2-naphthamide (1m)**

 Prepared following general procedure B as a white solid (403.6 mg, 50% yield for two steps). ^1^H NMR (400 MHz, Chloroform*-d*) δ 8.32 (d, *J =* 1.8 Hz, 1H), 7.96 – 7.80 (m, 4H), 7.65 – 7.52 (m, 2H), 7.19 (d, *J =* 10.6 Hz, 1H), 6.54 – 6.38 (m, 1H), 6.10 (td, *J =* 54.9, 1.7 Hz, 1H) ppm. ^13^C NMR (101 MHz, Chloroform*-d*) δ 167.01, 135.40, 132.47, 129.40, 129.25, 129.03, 128.62, 128.60, 127.95, 127.30, 123.44, 112.44 (t, *J =* 248.3 Hz), 63.09 (dd, *J =* 27.7, 24.2 Hz) ppm. ^19^F NMR (376 MHz, Chloroform*-d*) δ -121.17 (ddd, *J =* 281.2, 55.3, 9.6 Hz), -131.22 (ddd, *J =* 281.3, 55.1, 14.3 Hz) ppm. [M+Na]^+^ calcd. for C_13_H_10_ClF_2_NNaO: 292.0311, found: 292.0331.

**N-(1-chloro-2,2-difluoroethyl)-1-naphthamide (1n)**

 Prepared following general procedure B as a white solid (250.2 mg, 31% yield for two steps). ^1^H NMR (400 MHz, Chloroform*-d*) δ 8.29 (dd, *J =* 8.2, 1.5 Hz, 1H), 7.97 (dd, *J =* 8.1, 1.2 Hz, 1H), 7.93 – 7.86 (m, 1H), 7.66 (dd, *J =* 7.2, 1.2 Hz, 1H), 7.57 (dddd, *J =* 14.8, 8.2, 6.9, 1.5 Hz, 2H), 7.46 (dd, *J =* 8.3, 7.1 Hz, 1H), 6.89 (d, *J =* 10.7 Hz, 1H), 6.45 (dddd, *J =* 14.1, 10.4, 8.2, 1.7 Hz, 1H), 6.07 (td, *J =* 54.9, 1.7 Hz, 1H) ppm. ^13^C NMR (101 MHz, Chloroform*-d*) δ 168.73, 133.80, 132.28, 131.72, 130.03, 128.65, 127.94, 126.94, 125.94, 124.98, 124.68, 112.48 (t, *J =* 248.4 Hz), 62.71 (dd, *J =* 28.2, 23.9 Hz) ppm. ^19^F NMR (376 MHz, Chloroform*-d*) δ -120.76 (ddd, *J =* 281.3, 55.1, 8.1 Hz), -131.70 (ddd, *J =* 281.3, 54.8, 14.0 Hz) ppm. HRMS (ESI) (*m/z*): [M+H]^+^ calcd. for C_13_H_11_ClF_2_NO: 270.0492, found: 270.0504.

**N-(1-chloro-2,2,2-trifluoroethyl)benzamide (4a)**

 Prepared following general procedure B as a white solid (355.5 mg, 50% yield for two steps). ^1^H NMR (400 MHz, Chloroform*-d*) δ 7.92 – 7.75 (m, 2H), 7.67 – 7.54 (m, 1H), 7.49 (t, *J =* 7.7 Hz, 2H), 6.94 (d, *J =* 10.5 Hz, 1H), 6.56 (dq, *J =* 11.0, 5.5 Hz, 1H) ppm. ^13^C NMR (101 MHz, Chloroform*-d*) δ 166.49, 133.32, 131.93, 129.10, 127.62, 122.03 (q, *J =* 279.3 Hz), 61.48 (q, *J =* 38.5 Hz) ppm. ^19^F NMR (376 MHz, Chloroform*-d*) δ -77.32 (d, *J =* 5.7 Hz) ppm. HRMS (ESI) (*m/z*): [M+H]^+^ calcd. for C_9_H_8_ClF_3_NO: 238.0241, found: 238.0245.

**N-(1-chloro-2,2,2-trifluoroethyl)-3-methylbenzamide (4b)**

 Prepared following general procedure B as a white solid (361.1 mg, 48% yield for two steps). ^1^H NMR (500 MHz, Chloroform*-d*) δ 7.71 – 7.55 (m, 2H), 7.38 (dt, *J =* 15.0, 7.6 Hz, 2H), 6.90 (d, *J =* 10.5 Hz, 1H), 6.55 (dq, *J =* 11.0, 5.5 Hz, 1H), 2.41 (s, 3H) ppm. ^13^C NMR (126 MHz, Chloroform*-d*) δ 166.65, 139.14, 134.06, 131.92, 128.96, 128.29, 124.59, 122.05 (q, *J =* 279.2 Hz), 61.52 (q, *J =* 38.5 Hz), 21.43 ppm. ^19^F NMR (471 MHz, Chloroform*-d*) δ -77.31 (d, *J =* 5.4 Hz) ppm. HRMS (ESI) (*m/z*): [M+H]^+^ calcd. for C_10_H_10_ClF_3_NO: 252.0398, found: 252.0405.

**N-(1-chloro-2,2,2-trifluoroethyl)-4-methylbenzamide (4c)**

 Prepared following general procedure B as a white solid (316.3 mg, 42% yield for two steps). ^1^H NMR (500 MHz, Chloroform*-d*) δ 7.71 (d, *J =* 7.8 Hz, 2H), 7.28 (d, *J =* 7.8 Hz, 2H), 6.88 (d, *J =* 10.8 Hz, 1H), 6.55 (dq, *J =* 11.1, 5.6 Hz, 1H), 2.42 (s, 3H) ppm. ^13^C NMR (126 MHz, Chloroform*-d*) δ 166.36, 144.18, 129.74, 129.09, 127.65, 122.06 (q, *J =* 279.2 Hz), 61.58 (q, *J =* 38.6 Hz), 21.73 ppm. ^19^F NMR (471 MHz, Chloroform*-d*) δ -77.31 (d, *J =* 6.3 Hz) ppm. HRMS (ESI) (*m/z*): [M+H]^+^ calcd. for C_10_H_10_ClF_3_NO: 252.0398, found: 252.0387.

**N-(1-chloro-2,2,2-trifluoroethyl)-3,5-dimethylbenzamide (4d)**

 Prepared following general procedure B as a white solid (445.2 mg, 56% yield for two steps). ^1^H NMR (500 MHz, Chloroform*-d*) δ 7.40 (s, 2H), 7.21 (s, 1H), 6.98 (d, *J =* 12.8 Hz, 1H), 6.54 (dq, *J =* 11.1, 5.6 Hz, 1H), 2.36 (s, 6H) ppm. ^13^C NMR (126 MHz, Chloroform*-d*) δ 166.93, 138.90, 134.88, 131.91, 125.33, 122.06 (q, *J =* 279.3 Hz), 61.53 (q, *J =* 38.4 Hz), 21.28 ppm. ^19^F NMR (471 MHz, Chloroform*-d*) δ -77.28 (d, *J =* 5.3 Hz) ppm. HRMS (ESI) (*m/z*): [M+Na]^+^ calcd. for C_11_H_11_ClF_3_NNaO: 288.0373, found: 288.0355.

**N-(1-chloro-2,2,2-trifluoroethyl)-4-ethylbenzamide (4e)**

 Prepared following general procedure B as a white solid (493.0 mg, 62% yield for two steps). ^1^H NMR (500 MHz, Chloroform*-d*) δ 7.74 (d, *J =* 8.0 Hz, 2H), 7.31 (d, *J =* 7.9 Hz, 2H), 6.91 (d, *J =* 10.9 Hz, 1H), 6.56 (dq, *J =* 11.1, 5.6 Hz, 1H), 2.72 (q, *J =* 7.6 Hz, 2H), 1.25 (t, *J =* 7.6 Hz, 3H) ppm. ^13^C NMR (126 MHz, Chloroform*-d*) δ 166.39, 150.36, 129.29, 128.58, 127.77, 122.06 (q, *J =* 279.2 Hz), 61.57 (q, *J =* 38.4 Hz), 29.02, 15.35 ppm. ^19^F NMR (471 MHz, Chloroform*-d*) δ -77.31 (d, *J =* 5.4 Hz) ppm. HRMS (ESI) (*m/z*): [M+H]^+^ calcd. for C_11_H_12_ClF_3_NO: 266.0554, found: 266.0540.

**N-(1-chloro-2,2,2-trifluoroethyl)-4-isopropylbenzamide (4f)**

 Prepared following general procedure B as a white solid (401.8 mg, 48% yield for two steps). ^1^H NMR (500 MHz, Chloroform*-d*) δ 7.75 (d, *J =* 7.9 Hz, 2H), 7.33 (d, *J =* 7.9 Hz, 2H), 6.92 (d, *J =* 10.7 Hz, 1H), 6.56 (d, *J =* 12.1 Hz, 1H), 3.18 – 2.73 (m, 1H), 1.26 (d, *J =* 7.1 Hz, 6H) ppm. ^13^C NMR (126 MHz, Chloroform*-d*) δ 166.39, 154.94, 129.44, 127.82, 127.20, 122.08 (q, *J =* 279.2 Hz), 61.59 (q, *J =* 38.5 Hz), 34.37, 23.80 ppm. ^19^F NMR (471 MHz, Chloroform*-d*) δ -77.31 (d, *J =* 5.6 Hz) ppm. HRMS (ESI) (*m/z*): [M+H]^+^ calcd. for C_12_H_14_ClF_3_NO: 280.0711, found: 280.0715.

**N-(1-chloro-2,2,2-trifluoroethyl)-4-isobutylbenzamide (4g)**

 Prepared following general procedure B as a white solid (483.6 mg, 55% yield for two steps). ^1^H NMR (500 MHz, Chloroform*-d*) δ 7.67 (d, *J =* 7.9 Hz, 2H), 7.18 (d, *J =* 8.0 Hz, 2H), 6.88 (d, *J =* 10.8 Hz, 1H), 6.49 (dq, *J =* 11.0, 5.6 Hz, 1H), 2.47 (d, *J =* 7.2 Hz, 2H), 1.81 (dt, *J =* 13.6, 6.8 Hz, 1H), 0.83 (d, *J =* 6.6 Hz, 6H) ppm. ^13^C NMR (126 MHz, Chloroform*-d*) δ 166.46, 147.89, 129.78, 129.33, 127.56, 122.06 (q, *J =* 279.3 Hz), 61.58 (q, *J =* 38.4 Hz), 45.41, 30.27, 22.38 ppm. ^19^F NMR (471 MHz, Chloroform*-d*) δ -77.29 (d, *J =* 5.4 Hz) ppm. HRMS (ESI) (*m/z*): [M+H]^+^ calcd. for C_13_H_16_ClF_3_NO: 294.0867, found: 294.0861.

**4-(tert-butyl)-N-(1-chloro-2,2,2-trifluoroethyl)benzamide (4h)**

 Prepared following general procedure B as a white solid (483.6 mg, 55% yield for two steps). ^1^H NMR (500 MHz, Chloroform*-d*) δ 7.90 – 7.70 (m, 2H), 7.56 – 7.39 (m, 2H), 6.97 (d, *J =* 11.0 Hz, 1H), 6.57 (dq, *J =* 11.0, 5.5 Hz, 1H), 1.34 (s, 9H) ppm. ^13^C NMR (126 MHz, Chloroform*-d*) δ 165.22, 156.01, 127.86, 126.39, 124.88, 120.91 (q, *J =* 279.1 Hz), 60.40 (q, *J =* 38.4 Hz), 34.10, 30.02 ppm. ^19^F NMR (471 MHz, Chloroform*-d*) δ -77.31 (d, *J =* 6.1 Hz) ppm. HRMS (ESI) (*m/z*): [M+H]^+^ calcd. for C_13_H_16_ClF_3_NO: 294.0867, found: 294.0870.

**N-(1-chloro-2,2,2-trifluoroethyl)-4-methoxybenzamide (4i)**

 Prepared following general procedure B as a white solid (376.5 mg, 47% yield for two steps). ^1^H NMR (400 MHz, Chloroform*-d*) δ 7.87 – 7.70 (m, 2H), 6.96 (d, *J =* 8.7 Hz, 2H), 6.76 (d, *J =* 10.7 Hz, 1H), 6.56 (dq, *J =* 11.0, 5.5 Hz, 1H), 3.87 (s, 3H) ppm. ^13^C NMR (101 MHz, Chloroform*-d*) δ 165.78, 163.66, 129.69, 124.07, 122.09 (q, *J =* 279.1 Hz), 114.31, 61.72 (q, *J =* 38.3 Hz), 55.70 ppm. ^19^F NMR (376 MHz, Chloroform*-d*) δ -77.29 (d, *J =* 5.5 Hz) ppm. HRMS (ESI) (*m/z*): [M+H]^+^ calcd. for C_10_H_10_ClF_3_NO_2_: 268.0347, found: 268.0328.

**N-(1-chloro-2,2,2-trifluoroethyl)-3-methoxybenzamide (4j)**

 Prepared following general procedure B as a white solid (416.6 mg, 52% yield for two steps). ^1^H NMR (500 MHz, Chloroform*-d*) δ 7.44 – 7.35 (m, 2H), 7.33 (d, *J =* 7.7 Hz, 1H), 7.12 (dd, *J =* 8.2, 2.6 Hz, 1H), 6.98 (d, *J =* 10.8 Hz, 1H), 6.55 (dq, *J =* 11.0, 5.5 Hz, 1H), 3.85 (s, 3H) ppm. ^13^C NMR (126 MHz, Chloroform*-d*) δ 166.43, 160.14, 133.31, 130.10, 122.01 (q, *J =* 279.3 Hz), 119.46, 119.25, 112.99, 61.49 (q, *J =* 38.5 Hz), 55.67 ppm. ^19^F NMR (471 MHz, Chloroform*-d*) δ -77.29 (d, *J =* 5.5 Hz) ppm. HRMS (ESI) (*m/z*): [M+H]^+^ calcd. for C_10_H_10_ClF_3_NO_2_: 268.0347, found: 268.0333.

General Procedure C1 for Enantioselective Reductive Alkenylation of *α*-CF_2_H Amino Halides

NiBr_2_•DME (10 mol%, 0.02 mmol), **L9** (13 mol%, 0.026 mmol) were firstly combined in a 25 mL oven*-*dried sealing tube. The vessel was evacuated and backfilled with Ar (repeated for 3 times), 2 mLTHF was added via syringe and the complex was allowed to pre-stir at 25 ˚C for 30 min. Then CF_2_H-substituted secondary alkyl chloride **1** (1.2 equiv, 0.24 mmol), vinyl iodides **2** (1.0 equiv, 0.20 mmol), Mn podwer (2.0 equiv, 0.4 mmol ) and TMSCl (0.8 equiv, 0.16 mmol) were added and the tube was cooled to -20 ˚C. The tube was sealed with a Teflon lined cap and stired at -20 ˚C for 48 h. The reaction mixture was then diluted with EtOAc (~10 mL) and filtered through a pad of celite. The filtrate was added brine (20 mL) and extracted with EtOAc (3×15 mL), the combined organic layer was dried over Na_2_SO_4_, filtrated and concentrated under vacuum. The residue was then purified by flash column chromatography to give desired products.

General Procedure C2 for Enantioselective Reductive Alkenylation of *α*-CF_3_ Amino Halides

NiBr_2_•DME (10 mol%, 0.02 mmol), **L9** (13 mol%, 0.026 mmol) were firstly combined in a 25 mL oven*-*dried sealing tube. The vessel was evacuated and backfilled with Ar (repeated for 3 times), 1.2 mLTHF was added via syringe and the complex was allowed to pre-stir at 25 ˚C for 30 min. Then CF_3_-substituted secondary alkyl chloride **4** (1.4 equiv, 0.28 mmol), vinyl iodides **2** (1.0 equiv, 0.20 mmol), Mn podwer (2.0 equiv, 0.4 mmol ) and TMSCl (0.8 equiv, 0.16 mmol) were added and the tube was cooled to -40 ˚C. The tube was sealed with a Teflon lined cap and stired at -40 ˚C for 48 h. The reaction mixture was then diluted with EtOAc (~10 mL) and filtered through a pad of celite. The filtrate was added brine (20 mL) and extracted with EtOAc (3×15 mL), the combined organic layer was dried over Na_2_SO_4_, filtrated and concentrated under vacuum. The residue was then purified by flash column chromatography to give desired products.

**methyl (R,E)-4-(3-benzamido-4,4*-d*ifluorobut-1-en-1-yl)benzoate (3a)**

The product **3a** was purified with silica gel chromatography (PE:EA = 7:1) as a white solid (84% yield, 57.9 mg). ^1^H NMR (500 MHz, Chloroform*-d*) δ 8.02 – 7.96 (m, 2H), 7.89 – 7.81 (m, 2H), 7.59 – 7.51 (m, 1H), 7.50 – 7.40 (m, 4H), 6.80 (dd, *J =* 16.0, 1.4 Hz, 1H), 6.63 (d, *J =* 8.6 Hz, 1H), 6.37 (dd, *J =* 16.0, 6.7 Hz, 1H), 6.03 (ddd, *J =* 56.8, 54.9, 2.2 Hz, 1H), 5.28 (dddd, *J =* 19.0, 10.4, 8.6, 6.6 Hz, 1H), 3.90 (s, 3H) ppm. ^13^C NMR (126 MHz, Chloroform*-d*) δ 140.21, 134.69, 133.50, 132.33, 130.10, 129.92, 128.89, 127.30, 126.74, 122.94, 114.44 (t, *J =* 246.0 Hz), 53.42 (dd, *J =* 24.8, 21.5 Hz), 52.28 ppm. ^19^F NMR (471 MHz, Chloroform*-d*) δ -124.99 (ddd, *J =* 284.0, 55.0, 10.7 Hz), -128.59 (ddd, *J =* 283.6, 56.6, 18.5 Hz) ppm. HRMS (ESI) (*m/z*): [M+Na]^+^ calcd. for C_19_H_17_F_2_NNaO_3_: 368.1069, found: 368.1079. **[α]_D_^20^** = -27 (*c* = 1.0, CHCl_3_), HPLC chiralcel AD-H column (40% isopropanol in hexanes, 0.5 mL/min, λ = 269 nm), t_R_ = 9.7 min (minor), 10.3 min (major), 98% ee.

**methyl (R,E)-3-(3-benzamido-4,4*-d*ifluorobut-1-en-1-yl)benzoate (3b)**

The product **3b** was purified with silica gel chromatography (PE:EA = 7:1) as a white solid (79% yield, 54.5 mg). ^1^H NMR (600 MHz, Chloroform*-d*) δ 8.02 (d, *J =* 1.8 Hz, 1H), 7.90 (dt, *J =* 7.8, 1.5 Hz, 1H), 7.85 (dd, *J =* 8.2, 1.4 Hz, 2H), 7.54 – 7.48 (m, 2H), 7.42 (t, *J =* 7.7 Hz, 2H), 7.35 (t, *J =* 7.7 Hz, 1H), 6.87 (d, *J =* 8.7 Hz, 1H), 6.76 (dd, *J =* 16.0, 1.4 Hz, 1H), 6.32 (dd, *J =* 16.0, 6.8 Hz, 1H), 6.01 (ddd, *J =* 56.8, 55.0, 2.3 Hz, 1H), 5.25 (tt, *J =* 17.6, 7.7 Hz, 1H), 3.89 (s, 3H) ppm. ^13^C NMR (151 MHz, Chloroform*-d*) δ 167.48, 166.95, 136.15, 134.55, 133.52, 132.23, 131.19, 130.62, 129.40, 128.85, 128.79, 127.70, 127.33, 121.59, 114.48 (t, *J =* 246.0 Hz), 53.46 (dd, *J =* 24.9, 21.6 Hz), 52.33 ppm. ^19^F NMR (565 MHz, Chloroform*-d*) δ -124.78 (ddd, *J =* 283.1, 55.8, 11.3 Hz), -128.29 (ddd, *J =* 282.0, 55.7, 16.8 Hz) ppm. HRMS (ESI) (*m/z*): [M+Na]^+^ calcd. for C_19_H_17_F_2_NNaO_3_: 368.1069, found: 368.1081. **[α]_D_^20^** = -20 (*c* = 1.0, CHCl_3_), HPLC chiralcel AD-H column (25% isopropanol in hexanes, 0.5 mL/min, λ = 254 nm), t_R_ = 12.8 min (minor), 20.5 min (major), 98% *ee*.

**(R, E)-N-(4-(4-cyanophenyl)-1,1*-d*ifluorobut-3-en-2-yl) benzamide (3c)**

The product **3c** was purified with silica gel chromatography (PE:EA = 8:1) as a white solid (70% yield, 43.7 mg). ^1^H NMR (500 MHz, Chloroform*-d*) δ 7.86 – 7.81 (m, 2H), 7.61 (d, *J =* 8.4 Hz, 2H), 7.58 – 7.53 (m, 1H), 7.48 (dt, *J =* 9.3, 3.6 Hz, 4H), 6.79 (dd, *J =* 16.1, 1.5 Hz, 1H), 6.50 (d, *J =* 8.8 Hz, 1H), 6.40 (dd, *J =* 16.0, 6.6 Hz, 1H), 6.04 (ddd, *J =* 56.8, 54.9, 2.2 Hz, 1H), 5.29 (dddd, *J =* 20.9, 10.4, 8.5, 6.6 Hz, 1H) ppm. ^13^C NMR (126 MHz, Chloroform*-d*) δ 167.31, 140.24, 133.97, 133.36, 132.64, 132.47, 128.96, 127.36, 127.27, 124.32, 118.80, 114.33 (t, *J =* 246.0 Hz), 111.88, 53.32 (dd, *J =* 24.8, 21.6 Hz) ppm. ^19^F NMR (471 MHz, Chloroform*-d*) δ -124.79 (ddd, *J =* 284.8, 55.3, 11.1 Hz), -128.70 (ddd, *J =* 284.8, 56.7, 19.3 Hz) ppm. HRMS (ESI) (*m/z*): [M+H]^+^ calcd. for C_18_H_15_F_2_N_2_O: 313.1147, found: 313.1153. **[α]_D_^20^** = -38 (*c* = 1.0, CHCl_3_), HPLC chiralcel AD-H column (25% isopropanol in hexanes, 0.5 mL/min, λ = 254 nm), t_R_ = 15.3 min (minor), 19.4 min (major), 98% *ee*.

**(R,E)-N-(4-(3-cyanophenyl)-1,1*-d*ifluorobut-3-en-2-yl)benzamide (3d)**

The product **3d** was purified with silica gel chromatography (PE:EA = 8:1) as a white solid (74% yield, 46.2 mg). ^1^H NMR (500 MHz, Chloroform*-d*) δ 7.86 – 7.83 (m, 2H), 7.63 (d, *J =* 1.7 Hz, 1H), 7.62 – 7.58 (m, 1H), 7.54 (ddd, *J =* 7.8, 3.8, 2.4 Hz, 2H), 7.47 (dd, *J =* 8.4, 7.1 Hz, 2H), 7.43 (t, *J =* 7.8 Hz, 1H), 6.75 (dd, *J =* 16.1, 1.4 Hz, 1H), 6.65 (d, *J =* 8.8 Hz, 1H), 6.33 (dd, *J =* 16.0, 6.7 Hz, 1H), 6.03 (ddd, *J =* 56.8, 54.9, 2.3 Hz, 1H), 5.35 – 5.16 (m, 1H) ppm. ^13^C NMR (126 MHz, Chloroform*-d*) δ 167.37, 137.07, 133.41, 133.34, 132.42, 131.73, 131.02, 130.17, 129.66, 128.91, 127.29, 123.20 (d, *J =* 5.2 Hz), 118.62, 114.33 (t, *J =* 246.0 Hz), 113.01, 53.33 (dd, *J =* 25.0, 21.6 Hz) ppm. ^19^F NMR (471 MHz, Chloroform*-d*) δ -124.71 (ddd, *J =* 284.3, 55.0, 10.8 Hz), -128.61 (ddd, *J =* 284.3, 56.5, 19.1 Hz) ppm. HRMS (ESI) (*m/z*): [M+H]^+^ calcd. for C_18_H_15_F_2_N_2_O: 313.1147, found: 313.1155. **[α]_D_^20^** = -34 (*c* = 1.0, CHCl_3_), HPLC chiralcel AD-H column (25% isopropanol in hexanes, 0.5 mL/min, λ = 245 nm), t_R_ = 15.0 min (minor), 29.6 min (major), 98% *ee*.

**(R,E)-N-(1,1*-d*ifluoro-4-(4-(trifluoromethyl)phenyl)but-3-en-2-yl)benzamide (3e)**

The product **3e** was purified with silica gel chromatography (PE:EA = 10:1) as a white solid (71% yield, 50.4 mg). ^1^H NMR (500 MHz, Chloroform*-d*) δ 7.84 (d, *J =* 7.6 Hz, 2H), 7.57 (t, *J =* 9.7 Hz, 3H), 7.48 (d, *J =* 8.1 Hz, 4H), 6.81 (d, *J =* 16.1 Hz, 1H), 6.55 (d, *J =* 8.7 Hz, 1H), 6.36 (dd, *J =* 16.0, 6.7 Hz, 1H), 6.04 (t, *J =* 55.4 Hz, 1H), 5.29 (dq, *J =* 17.9, 8.8 Hz, 1H) ppm. ^13^C NMR (126 MHz, Chloroform*-d*) δ 167.35, 139.23, 134.32, 133.43, 132.41, 130.33 (q, *J =* 32.8 Hz), 128.94, 127.28, 127.02, 125.67 (q, *J =* 3.8 Hz), 124.03 (q, *J =* 272.0 Hz), 122.94, 114.29 (t, *J =* 246.1 Hz), 53.23 (dd, *J =* 24.8, 21.4 Hz) ppm. ^19^F NMR (471 MHz, Chloroform*-d*) δ -62.60, -125.02 (ddd, *J =* 283.8, 54.9, 10.5 Hz), -128.69 (ddd, *J =* 284.2, 56.6, 18.9 Hz) ppm. HRMS (ESI) (*m/z*): [M+H]^+^ calcd. for C_18_H_15_F_5_NO: 356.1068, found: 356.1075. **[α]_D_^20^** = -21 (*c* = 1.0, CHCl_3_), HPLC chiralcel AD-H column (25% isopropanol in hexanes, 0.5 mL/min, λ = 254 nm), t_R_ = 9.9 min (minor), 13.3 min (major), 98% *ee*.

**(R,E)-N-(1,1*-d*ifluoro-4-(4-fluorophenyl)but-3-en-2-yl)benzamide (3f)**

The product **3f** was purified with silica gel chromatography (PE:EA = 10:1) as a white solid (68% yield, 41.5 mg). ^1^H NMR (500 MHz, Chloroform*-d*) δ 7.92 – 7.75 (m, 2H), 7.61 – 7.50 (m, 1H), 7.46 (t, *J =* 7.6 Hz, 2H), 7.41 – 7.32 (m, 2H), 7.01 (t, *J =* 8.7 Hz, 2H), 6.74 (d, *J =* 16.0 Hz, 1H), 6.54 (d, *J =* 8.8 Hz, 1H), 6.17 (dd, *J =* 16.0, 7.0 Hz, 1H), 6.01 (ddd, *J =* 56.9, 54.9, 2.2 Hz, 1H), 5.23 (dtd, *J =* 19.6, 9.2, 6.9 Hz, 1H) ppm. ^13^C NMR (126 MHz, Chloroform*-d*) δ 167.34, 162.91 (d, *J =* 248.2 Hz), 134.67, 133.60, 132.29, 132.03 (d, *J =* 3.5 Hz), 128.89, 128.46 (d, *J =* 8.1 Hz), 127.27, 119.88, 115.77 (d, *J =* 21.7 Hz), 114.52 (t, *J =* 245.9 Hz), 53.49 (dd, *J =* 24.7, 21.4 Hz) ppm. ^19^F NMR (471 MHz, Chloroform*-d*) δ -112.99 (t, *J =* 5.2 Hz, 1F), -125.30 (ddd, *J =* 283.3, 55.1, 10.9 Hz, 1F), -128.65 (dddd, *J =* 283.3, 57.0, 18.3, 3.0 Hz, 1F) ppm. HRMS (ESI) (*m/z*): [M+H]^+^ calcd. for C_17_H_15_F_3_NO: 356.1068, found: 356.1075. **[α]_D_^20^** = -14 (*c* = 1.0, CHCl_3_), HPLC chiralcel AD-H column (20% isopropanol in hexanes, 0.5 mL/min, λ = 254 nm), t_R_ = 12.6 min (minor), 17.1 min (major), 98% *ee*.

**(R,E)-N-(4-(4-chlorophenyl)-1,1*-d*ifluorobut-3-en-2-yl)benzamide (3g)**

The product **3g** was purified with silica gel chromatography (PE:EA = 9:1) as a white solid (71% yield, 45.6 mg). ^1^H NMR (500 MHz, Chloroform*-d*) δ 7.86 – 7.80 (m, 2H), 7.58 – 7.52 (m, 1H), 7.46 (t, *J =* 7.7 Hz, 2H), 7.35 – 7.27 (m, 4H), 6.73 (dd, *J =* 16.0, 1.5 Hz, 1H), 6.52 (d, *J =* 8.8 Hz, 1H), 6.23 (dd, *J =* 16.0, 6.9 Hz, 1H), 6.01 (ddd, *J =* 56.8, 54.9, 2.2 Hz, 1H), 5.30 – 5.18 (m, 1H) ppm. ^13^C NMR (126 MHz, Chloroform*-d*) δ 167.33, 134.56, 134.33, 134.31, 133.55, 132.33, 129.00, 128.90, 128.05, 127.27, 120.83, 114.47 (t, *J =* 246.0 Hz), 53.43 (dd, *J =* 24.7, 21.4 Hz) ppm. ^19^F NMR (471 MHz, Chloroform*-d*) δ -125.22 (ddd, *J =* 283.5, 54.9, 10.7 Hz, 1F), -128.66 (ddd, *J =* 283.5, 56.5, 18.4 Hz, 1F) ppm. HRMS (ESI) (*m/z*): [M+H]^+^ calcd. for C_17_H_15_ClF_2_NO: 322.0805, found: 322.0812. **[α]_D_^20^** = -17(*c* = 1.0, CHCl_3_), HPLC chiralcel AD-H column (20% isopropanol in hexanes, 0.5 mL/min, λ = 254 nm), t_R_ = 13.5 min (minor), 17.8 min (major), 98% *ee*.

**(R,E)-N-(4-(3-chlorophenyl)-1,1*-d*ifluorobut-3-en-2-yl)benzamide (3h)**

The product **3h** was purified with silica gel chromatography (PE:EA = 10:1) as a white solid (63% yield, 40.4 mg). ^1^H NMR (500 MHz, Chloroform*-d*) δ 7.73 (d, *J =* 7.6 Hz, 2H), 7.48 – 7.24 (m, 4H), 7.14 (s, 3H), 6.60 (d, *J =* 16.1 Hz, 1H), 6.52 (d, *J =* 14.0 Hz, 1H), 6.16 (dd, *J =* 16.0, 6.8 Hz, 1H), 5.90 (t, *J =* 55.7 Hz, 1H), 5.26 – 5.07 (m, 1H) ppm. ^13^C NMR (126 MHz, Chloroform*-d*) δ 167.29, 137.56, 134.66, 134.25, 133.42, 132.21, 129.92, 128.78, 128.39, 127.18, 126.57, 125.01, 121.70, 114.34 (t, *J =* 246.0 Hz), 53.43 (dd, *J =* 24.7, 21.4 Hz) ppm. ^19^F NMR (471 MHz, Chloroform*-d*) δ -124.51 – -125.60 (m), -128.54 (dddd, *J =* 283.3, 55.6, 26.5, 16.9 Hz) ppm. HRMS (ESI) (*m/z*): [M+H]^+^ calcd. for C_17_H_15_ClF_2_NO: 322.0805, found: 322.0815. **[α]_D_^20^** = -23 (*c* = 1.0, CHCl_3_), HPLC chiralcel AD-H column (20% isopropanol in hexanes, 0.5 mL/min, λ = 269 nm), t_R_ = 11.7 min (minor), 18.8 min (major), 98% *ee.*

**(R,E)-N-(4-(4-acetylphenyl)-1,1*-d*ifluorobut-3-en-2-yl)benzamide (3i)**

The product **3i** was purified with silica gel chromatography (PE:EA = 8:1) as a white solid (67% yield, 44.1 mg). ^1^H NMR (600 MHz, Chloroform*-d*) δ 7.90 (d, *J =* 8.0 Hz, 2H), 7.85 (d, *J =* 7.3 Hz, 2H), 7.55 (t, *J =* 7.4 Hz, 1H), 7.50 – 7.41 (m, 4H), 6.81 (d, *J =* 16.0 Hz, 1H), 6.66 (d, *J =* 8.7 Hz, 1H), 6.39 (dd, *J =* 16.0, 6.7 Hz, 1H), 6.16 – 5.89 (m, 1H), 5.29 (dq, *J =* 17.7, 8.6 Hz, 1H), 2.58 (s, 3H) ppm. ^13^C NMR (151 MHz, Chloroform*-d*) δ 197.76, 167.36, 140.37, 136.77, 134.57, 133.45, 132.36, 128.91, 127.31, 126.94, 123.15, 123.12, 114.42 (t, *J =* 246.0 Hz), 53.41(dd, *J =* 24.9, 21.4 Hz), 26.75 ppm. ^19^F NMR (565 MHz, Chloroform*-d*) δ -124.80 (ddd, *J =* 283.4, 54.3, 11.1 Hz), -128.50 (ddd, *J =* 283.1, 57.1, 19.0 Hz) ppm. HRMS (ESI) (*m/z*): [M+H]^+^ calcd. for C_19_H_18_F_2_NO_2_: 330.1300, found: 330.1311. **[α]_D_^20^** = -31 (*c* = 1.0, CHCl_3_), HPLC chiralcel AD-H column (25% isopropanol in hexanes, 0.5 mL/min, λ = 269 nm), t_R_ = 16.8 min (minor), 18.4 min (major), 98% *ee*.

**(R,E)-N-(1,1*-d*ifluoro-4-(4-(methylsulfonyl)phenyl)but-3-en-2-yl)benzamide (3j)**

The product **3j** was purified with silica gel chromatography (PE: EA = 4:1) as a white oil (73% yield, 53.3 mg). ^1^H NMR (500 MHz, Chloroform*-d*) δ 7.91 – 7.82 (m, 4H), 7.59 – 7.51 (m, 3H), 7.50 – 7.46 (m, 2H), 6.82 (dd, *J =* 16.0, 1.6 Hz, 1H), 6.62 (d, *J =* 8.8 Hz, 1H), 6.43 (dd, *J =* 16.0, 6.6 Hz, 1H), 6.05 (ddd, *J =* 56.8, 54.9, 2.3 Hz, 1H), 5.31 (dddt, *J =* 18.8, 17.0, 8.6, 1.8 Hz, 1H), 3.04 (s, 3H) ppm. ^13^C NMR (126 MHz, Chloroform*-d*) δ 167.36, 141.28, 139.94, 133.67, 133.38, 132.44, 128.94, 127.96, 127.57, 127.34, 124.70, 114.37 (t, *J =* 246.2 Hz), 53.33 (dd, *J =* 24.7, 21.8 Hz), 44.65 ppm. ^19^F NMR (471 MHz, Chloroform*-d*) δ -124.63 (ddd, *J =* 284.4, 54.7, 10.2 Hz), -128.66 (ddd, *J =* 284.4, 56.4, 19.0 Hz) ppm. HRMS (ESI) (*m/z*): [M+H]^+^ calcd. for C_18_H_18_F_2_NO_3_S: 366.0970, found: 366.0982. **[α]_D_^20^** = -40 (*c* = 1.0, CHCl_3_), HPLC chiralcel AD-H column (40% isopropanol in hexanes, 0.5 mL/min, λ = 254 nm), t_R_ = 14.4 min (minor), 16.2 min (major), 98% *ee*.

**(R,E)-N-(1,1*-d*ifluoro-4-(3-methoxyphenyl)but-3-en-2-yl)benzamide (3k)**

The product **3k** was purified with silica gel chromatography (PE:EA = 8:1) as a white solid (69% yield, 43.7 mg). ^1^H NMR (500 MHz, Chloroform*-d*) δ 7.94 – 7.83 (m, 2H), 7.68 – 7.54 (m, 1H), 7.50 (dd, *J =* 8.4, 6.9 Hz, 2H), 7.35 – 7.20 (m, 1H), 7.02 (dt, *J =* 7.7, 1.3 Hz, 1H), 6.96 (t, *J =* 2.1 Hz, 1H), 6.87 (ddd, *J =* 8.2, 2.6, 0.9 Hz, 1H), 6.79 (dd, *J =* 16.0, 1.4 Hz, 1H), 6.49 (d, *J =* 8.6 Hz, 1H), 6.28 (dd, *J =* 16.0, 6.9 Hz, 1H), 6.05 (ddd, *J =* 56.9, 55.0, 2.1 Hz, 1H), 5.34 – 5.23 (m, 1H), 3.84 (s, 3H) ppm. ^13^C NMR (126 MHz, Chloroform*-d*) δ 167.31, 159.98, 137.25, 135.76, 133.65, 132.27, 129.82, 128.90, 127.26, 120.45, 119.46, 114.54 (t, *J =* 245.9 Hz), 114.34, 112.09, 55.40, 53.44 (dd, *J =* 24.6, 21.3 Hz) ppm. ^19^F NMR (471 MHz, Chloroform*-d*) δ -125.47 (ddd, *J =* 283.3, 55.0, 10.9 Hz), -128.65 (ddd, *J =* 283.2, 56.7, 18.4 Hz) ppm. HRMS (ESI) (*m/z*): [M+H]^+^ calcd. for C_18_H_18_F_2_NO_2_: 318.1300, found: 318.1304. **[α]_D_^20^** = -18 (*c* = 1.0, CHCl_3_), HPLC chiralcel AD-H column (25% isopropanol in hexanes, 0.5 mL/min, λ = 254 nm), t_R_ = 10.0 min (minor), 11.7 min (major), 96% *ee.*

**(R,E)-N-(1,1*-d*ifluoro-4-(4-(trifluoromethoxy)phenyl)but-3-en-2-yl)benzamide (3l)**

The product **3l** was purified with silica gel chromatography (PE:EA = 10:1) as a white solid (61% yield, 45.2 mg). ^1^H NMR (600 MHz, Chloroform*-d*) δ 7.84 (d, *J =* 7.6 Hz, 2H), 7.54 (t, *J =* 7.4 Hz, 1H), 7.44 (t, *J =* 7.6 Hz, 2H), 7.38 (d, *J =* 8.3 Hz, 2H), 7.15 (d, *J =* 8.2 Hz, 2H), 6.74 (d, *J =* 16.0 Hz, 1H), 6.70 (d, *J =* 8.7 Hz, 1H), 6.23 (dd, *J =* 16.0, 6.9 Hz, 1H), 6.00 (t, *J =* 55.7 Hz, 1H), 5.24 (dq, *J =* 18.1, 8.9 Hz, 1H) ppm. ^13^C NMR (151 MHz, Chloroform*-d*) δ 167.43, 149.20, 134.55, 134.25, 133.52, 132.31, 128.87, 128.12, 127.30, 121.22, 120.51 (q, *J =* 257.5 Hz), 114.45 (t, *J =* 245.9 Hz), 53.55(dd, *J =* 24.7, 21.0 Hz) ppm. ^19^F NMR (565 MHz, Chloroform*-d*) δ -57.75, -124.98 (ddd, *J =* 284.5, 55.3, 10.8 Hz), -128.44 (ddd, *J =* 283.1, 57.1, 18.9 Hz) ppm. HRMS (ESI) (*m/z*): [M+H]^+^ calcd. for C_18_H_15_F_5_NO_2_: 372.1017, found: 372.1029. **[α]_D_^20^** = -15 (*c* = 1.0, CHCl_3_), HPLC chiralcel AD-H column (25% isopropanol in hexanes, 0.5 mL/min, λ = 254 nm), t_R_ = 10.1 min (minor), 12.3 min (major), 98% *ee*.

**(R,E)-N-(1,1*-d*ifluoro-4-(m-tolyl)but-3-en-2-yl)benzamide (3m)**

The product **3m** was purified with silica gel chromatography (PE:EA = 10:1) as a white solid (69% yield, 41.5 mg). ^1^H NMR (500 MHz, Chloroform*-d*) δ 7.84 (dt, *J =* 7.2, 1.4 Hz, 2H), 7.60 – 7.51 (m, 1H), 7.46 (dd, *J =* 8.4, 7.0 Hz, 2H), 7.25 – 7.17 (m, 3H), 7.14 – 7.07 (m, 1H), 6.75 (dd, *J =* 16.0, 1.4 Hz, 1H), 6.54 (d, *J =* 8.7 Hz, 1H), 6.25 (dd, *J =* 16.0, 6.9 Hz, 1H), 6.01 (ddd, *J =* 56.8, 55.0, 2.2 Hz, 1H), 5.33 – 5.14 (m, 1H), 2.34 (s, 3H) ppm. ^13^C NMR (126 MHz, Chloroform*-d*) δ 167.32, 138.40, 135.91, 135.73, 133.65, 132.22, 129.37, 128.86, 128.68, 127.49, 127.26, 123.99, 119.82, 114.55 (t, *J =* 245.9 Hz), 53.50 (dd, *J =* 24.6, 21.4 Hz), 21.47 ppm. ^19^F NMR (471 MHz, Chloroform*-d*) δ -125.43 (ddd, *J =* 282.7, 54.8, 10.9 Hz), -128.60 (ddd, *J =* 282.7, 56.6, 18.2 Hz) ppm. HRMS (ESI) (*m/z*): [M+H]^+^ calcd. for C_18_H_18_F_2_NO: 302.1351, found: 302.1357. **[α]_D_^20^** = -14 (*c* = 1.0, CHCl_3_), HPLC chiralcel AD-H column (20% isopropanol in hexanes, 0.5 mL/min, λ = 254 nm), t_R_ = 10.8 min (minor), 14.9 min (major), 95% *ee*.

**(R,E)-N-(1,1*-d*ifluoro-4-(o-tolyl)but-3-en-2-yl)benzamide (3n)**

The product **3n** was purified with silica gel chromatography (PE:EA = 10:1) as a white solid (55% yield, 33.1 mg). ^1^H NMR (500 MHz, Chloroform*-d*) δ 7.89 – 7.75 (m, 2H), 7.59 – 7.51 (m, 1H), 7.52 – 7.39 (m, 3H), 7.18 (dqd, *J =* 13.7, 7.0, 2.0 Hz, 3H), 7.03 (dd, *J =* 15.8, 1.4 Hz, 1H), 6.49 (d, *J =* 8.7 Hz, 1H), 6.16 – 6.12 (m, 1H), 6.11 – 5.91 (m, 1H), 5.33 – 5.20 (m, 1H), 2.34 (s, 3H) ppm. ^13^C NMR (126 MHz, Chloroform*-d*) δ 167.37, 135.98, 135.06, 134.08, 133.74, 132.29, 130.60, 128.94, 128.52, 127.27, 126.36, 125.97, 121.41, 114.59 (t, *J =* 245.8 Hz), 53.83 (dd, *J =* 24.7, 21.2 Hz), 19.94 ppm. ^19^F NMR (471 MHz, Chloroform*-d*) δ -125.45 (ddd, *J =* 283.3, 55.3, 11.2 Hz), -128.78 (ddd, *J =* 282.9, 56.6, 18.2 Hz) ppm. HRMS (ESI) (*m/z*): [M+H]^+^ calcd. for C_18_H_18_F_2_NO: 302.1351, found: 302.1358. **[α]_D_^20^** = -10 (*c* = 1, CHCl_3_), HPLC chiralcel AD-H column (10% isopropanol in hexanes, 0.5 mL/min, λ = 254 nm), t_R_ = 20.3 min (minor), 29.3 min (major), 98% *ee*.

**(R,E)-N-(1,1*-d*ifluoro-4-phenylbut-3-en-2-yl)benzamide (3o)**

The product **3o** was purified with silica gel chromatography (PE: EA = 10:1) as a white solid (61% yield, 35.0 mg). ^1^H NMR (400 MHz, Chloroform*-d*) δ 7.84 (d, *J =* 7.1 Hz, 2H), 7.54 (t, *J =* 7.3 Hz, 1H), 7.46 (t, *J =* 7.5 Hz, 2H), 7.39 (d, *J =* 7.0 Hz, 2H), 7.36 – 7.28 (m, 3H), 6.78 (d, *J =* 15.9 Hz, 1H), 6.58 (d, *J =* 8.7 Hz, 1H), 6.26 (dd, *J =* 16.0, 6.9 Hz, 1H), 6.01 (ddd, *J =* 56.7, 55.0, 2.1 Hz, 1H), 5.26 (tt, *J =* 17.9, 7.8 Hz, 1H) ppm. ^13^C NMR (101 MHz, Chloroform*-d*) δ 167.34, 135.80, 133.65, 132.22, 128.85, 128.79, 128.57, 127.27, 126.81, 120.11, 120.06, 114.54 (t, *J =* 245.9 Hz), 53.50 (dd, *J =* 24.6, 21.4 Hz) ppm. ^19^F NMR (376 MHz, Chloroform*-d*) δ -125.36 (ddd, *J =* 283.1, 55.4, 11.7 Hz), -128.58 (ddd, *J =* 283.1, 56.8, 18.5 Hz) ppm. HRMS (ESI) (*m/z*): [M+H]^+^ calcd. for C_17_H_16_F_2_NO: 288.1194, found: 288.1203. **[****α]_D_^20^** = -12 (*c* = 1.0, CHCl_3_), HPLC chiralcel AD-H column (10% isopropanol in hexanes, 0.5 mL/min, λ = 254 nm), t_R_ = 24.4min (minor), 42.2 min (major), 98% *ee*.

**(R,E)-N-(1,1*-d*ifluoro-4-(naphthalen-2-yl)but-3-en-2-yl)benzamide (3p)**

The product **3p** was purified with silica gel chromatography (PE:EA = 10:1) as a white solid (58% yield, 39.1 mg). ^1^H NMR (400 MHz, Chloroform*-d*) δ 7.92 – 7.84 (m, 2H), 7.80 (td, *J =* 6.6, 5.8, 2.7 Hz, 3H), 7.75 (d, *J =* 1.7 Hz, 1H), 7.63 – 7.53 (m, 2H), 7.51 – 7.43 (m, 4H), 6.94 (d, *J =* 16.0 Hz, 1H), 6.56 (d, *J =* 8.7 Hz, 1H), 6.39 (dd, *J =* 16.0, 6.9 Hz, 1H), 6.06 (ddd, *J =* 56.8, 55.0, 2.2 Hz, 1H), 5.39 – 5.26 (m, 1H) ppm. ^13^C NMR (101 MHz, Chloroform*-d*) δ 167.34, 135.88, 133.65, 133.55, 133.45, 133.25, 132.27, 128.89, 128.52, 128.25, 127.82, 127.35, 127.29, 126.58, 126.46, 123.42, 120.39, 114.59 (t, *J =* 245.9 Hz), 53.57 (dd, *J =* 24.6, 21.4 Hz) ppm. ^19^F NMR (376 MHz, Chloroform*-d*) δ -125.29 (ddd, *J =* 283.2, 55.2, 11.5 Hz), -128.56 (ddd, *J =* 283.0, 56.8, 18.6 Hz) ppm. HRMS (ESI) (m/z): [M+H]^+^ calcd. for C_21_H_18_F_2_NO: 338.1351, found: 338.1360. **α]_D_^20^** = -28 (*c* = 1.0, CHCl_3_) HPLC chiralcel AD-H column (25% isopropanol in hexanes, 0.5 mL/min, λ = 254 nm), t_R_ = 12.4 min (minor), 17.4 min (major), 97% *ee*.

**methyl (R,E)-5-(3-benzamido-4,4*-d*ifluorobut-1-en-1-yl)-2-fluorobenzoate (3q)**

The product **3q** was purified with silica gel chromatography (PE: EA = 7:1) as a white solid (71% yield, 51.5 mg). ^1^H NMR (400 MHz, Chloroform*-d*) δ 7.92 (dd, *J =* 6.9, 2.4 Hz, 1H), 7.84 (dt, *J =* 7.0, 1.4 Hz, 2H), 7.56 – 7.51 (m, 1H), 7.47 – 7.42 (m, 2H), 7.07 (dd, *J =* 10.4, 8.6 Hz, 1H), 6.80 – 6.62 (m, 2H), 6.24 (dd, *J =* 16.0, 6.8 Hz, 1H), 6.01 (ddd, *J =* 56.8, 54.9, 2.2 Hz, 1H), 5.24 (ddt, *J =* 18.7, 10.4, 7.8 Hz, 1H), 3.92 (s, 3H) ppm. ^13^C NMR (101 MHz, Chloroform*-d*) δ 167.37, 164.76, 161.71 (d, *J =* 262.0 Hz), 133.45, 132.57, 132.48, 132.30, 132.09 (d, *J =* 4.0 Hz), 130.31, 128.85, 127.29, 121.43, 118.82 (d, *J =* 10.6 Hz), 117.50 (d, *J =* 23.1 Hz), 114.43 (t, *J =* 246.0 Hz), 53.39 (dd, *J =* 24.8, 21.5 Hz), 52.59 ppm. ^19^F NMR (376 MHz, Chloroform*-d*) δ -109.63 (ddd, *J =* 10.9, 6.8, 4.3 Hz), -124.90 (ddd, *J =* 283.6, 55.0, 10.6 Hz), -128.62 (ddd, *J =* 283.5, 56.5, 18.4 Hz) ppm. HRMS (ESI) (*m/z*): [M+Na]^+^ calcd. for C_19_H_16_Cl_2_F_3_NNaO_3_: 386.0974, found: 386.0983. **[α]_D_^20^** = -21 (*c* = 1.0, CHCl_3_), HPLC chiralcel AD-H column (25% isopropanol in hexanes, 0.5 mL/min, λ = 254 nm), t_R_ = 13.8 min (minor), 22.4 min (major), 97% *ee*.

**methyl (R,E)-4-(3-(4-cyanobenzamido)-4,4*-d*ifluorobut-1-en-1-yl)benzoate (3r)**

The product **3r** was purified with silica gel chromatography (PE:EA = 6:1) as a white solid (71% yield, 52.5 mg). ^1^H NMR (500 MHz, Chloroform*-d*) δ 7.97 (dd, *J =* 18.8, 8.0 Hz, 4H), 7.76 (d, *J =* 8.0 Hz, 2H), 7.44 (d, *J =* 8.0 Hz, 2H), 6.82 (d, *J =* 16.0 Hz, 1H), 6.65 (d, *J =* 8.8 Hz, 1H), 6.34 (dd, *J =* 16.0, 7.1 Hz, 1H), 6.04 (t, *J =* 55.5 Hz, 1H), 5.27 (dq, *J =* 18.6, 9.1 Hz, 1H), 3.91 (s, 3H) ppm. ^13^C NMR (126 MHz, Chloroform*-d*) δ 166.86, 165.63, 139.92, 137.37, 135.31, 132.73, 130.16, 130.12, 128.06, 126.78, 122.33, 117.95, 115.88, 114.21 (t, *J =* 246.1 Hz), 53.86 (d, *J =* 22.8 Hz), 52.38 ppm. ^19^F NMR (471 MHz, Chloroform*-d*) δ -125.77 (ddd, *J =* 283.5, 54.8, 11.9 Hz), -127.82 (ddd, *J =* 284.2, 56.2, 16.9 Hz) ppm. HRMS (ESI) (*m/z*): [M+H]^+^ calcd. for C_20_H_17_F_2_N_2_O_3_: 371.1202, found: 371.1208. **[α]_D_^20^** = -38 (*c* = 1.0, CHCl_3_), HPLC chiralcel AD-H column (40% isopropanol in hexanes, 0.5 mL/min, λ = 254 nm), t_R_ = 12.5 min (minor), 14.7 min (major), 91% *ee*.

**methyl (R,E)-4-(4,4*-d*ifluoro-3-(4-fluorobenzamido)but-1-en-1-yl)benzoate (3s)**

The product **3s** was purified with silica gel chromatography (PE:EA = 8:1) as a white solid (63% yield, 45.7 mg). ^1^H NMR (400 MHz, Chloroform*-d*) δ 7.98 (d, *J =* 8.4 Hz, 2H), 7.91 – 7.79 (m, 2H), 7.53 – 7.28 (m, 2H), 7.14 (dd, *J =* 9.5, 7.6 Hz, 2H), 6.92 – 6.73 (m, 1H), 6.52 (d, *J =* 8.7 Hz, 1H), 6.35 (dd, *J =* 16.0, 6.8 Hz, 1H), 6.03 (ddd, *J =* 56.7, 54.9, 2.1 Hz, 1H), 5.26 (dddd, *J =* 18.0, 10.9, 8.8, 6.9 Hz, 1H), 3.91 (s, 3H) ppm. ^13^C NMR (101 MHz, Chloroform*-d*) δ 166.86, 166.27, 165.25 (d, *J =* 253.2 Hz), 140.10, 134.86, 130.12, 129.97, 129.78, 129.69, 126.75, 122.73, 116.00 (d, *J =* 22.0 Hz), 114.38 (t, *J =* 246.0 Hz), 53.50 (dd, *J =* 24.6, 21.6 Hz), 52.33 ppm. ^19^F NMR (376 MHz, Chloroform*-d*) δ -106.73, -125.24 (ddd, *J =* 283.9, 55.0, 11.1 Hz), -128.45 (ddd, *J =* 283.9, 56.5, 18.2 Hz) ppm. HRMS (ESI) (*m/z*): [M+H]^+^ calcd. for C_19_H_17_F_3_NO_3_: 364.1155, found: 364.1160. **[α]_D_^20^** = -24 (*c* = 1.0, CHCl_3_), HPLC chiralcel AD-H column (40% isopropanol in hexanes, 0.5 mL/min, λ = 254 nm), t_R_ = 9.3 min (minor), 10.2 min (major), 96% *ee.*

**methyl (R,E)-4-(3-(4-chlorobenzamido)-4,4*-d*ifluorobut-1-en-1-yl)benzoate (3t)**

The product **3t** was purified with silica gel chromatography (PE: EA = 8:1) as a white solid (59% yield, 44.7 mg). ^1^H NMR (500 MHz, Chloroform*-d*) δ 7.97 (d, *J =* 8.0 Hz, 2H), 7.78 (d, *J =* 8.2 Hz, 2H), 7.41 (dd, *J =* 8.0, 4.8 Hz, 4H), 6.78 (d, *J =* 16.0 Hz, 1H), 6.66 (d, *J =* 8.7 Hz, 1H), 6.34 (dd, *J =* 16.0, 6.9 Hz, 1H), 6.02 (t, *J =* 55.7 Hz, 1H), 5.24 (dq, *J =* 17.8, 8.7 Hz, 1H), 3.90 (s, 3H) ppm. ^13^C NMR (126 MHz, Chloroform*-d*) δ 166.87, 166.37, 140.10, 138.65, 134.87, 131.86, 130.11, 129.97, 129.13, 128.77, 126.73, 122.74, 114.36 (t, *J =* 246.0 Hz), 53.55 (dd, *J =* 24.7, 21.6 Hz), 52.31 ppm. ^19^F NMR (471 MHz, Chloroform*-d*) δ -125.22 (ddd, *J =* 284.1, 55.6, 12.5 Hz), -128.25 (ddd, *J =* 284.2, 57.1, 18.9 Hz) ppm. HRMS (ESI) (*m/z*): [M+H]^+^ calcd. for C_19_H_17_ClF_2_NO_3_: 380.0860, found: 380.0870. **[α]_D_^20^** = -30 (*c* = 1.0, CHCl_3_), HPLC chiralcel AD-H column (40% isopropanol in hexanes, 0.5 mL/min, λ = 254 nm), t_R_ = 10.3 min (minor), 11.8 min (major), 95% *ee*.

**methyl (R,E)-4-(3-(3-chlorobenzamido)-4,4*-d*ifluorobut-1-en-1-yl)benzoate (3u)**

The product **3u** was purified with silica gel chromatography (PE: EA = 8:1) as a white solid (61% yield, 46.2 mg). ^1^H NMR (500 MHz, Chloroform*-d*) δ 8.07 – 7.92 (m, 2H), 7.83 (t, *J =* 1.9 Hz, 1H), 7.71 (dt, *J =* 7.8, 1.4 Hz, 1H), 7.50 (ddd, *J =* 8.0, 2.1, 1.0 Hz, 1H), 7.46 – 7.35 (m, 3H), 6.78 (dd, *J =* 16.0, 1.4 Hz, 1H), 6.72 (d, *J =* 8.7 Hz, 1H), 6.34 (dd, *J =* 16.0, 6.9 Hz, 1H), 6.02 (ddd, *J =* 56.6, 54.9, 2.2 Hz, 1H), 5.25 (dddd, *J =* 17.6, 10.6, 8.9, 6.8 Hz, 1H), 3.90 (s, 3H) ppm. ^13^C NMR (126 MHz, Chloroform*-d*) δ 166.89, 166.14, 140.09, 135.28, 135.07, 134.92, 132.32, 130.18, 130.10, 129.96, 127.68, 126.74, 125.42, 122.65, 114.32 (t, *J =* 246.2 Hz), 53.61 (dd, *J =* 24.7, 21.5 Hz), 52.31 ppm. ^19^F NMR (471 MHz, Chloroform*-d*) δ -125.19 (ddd, *J =* 283.7, 54.8, 11.1 Hz), -128.17 (ddd, *J =* 283.7, 56.4, 17.8 Hz) ppm. HRMS (ESI) (*m/z*): [M+H]^+^ calcd. for C_19_H_17_ClF_2_NO_3_: 380.0860, found: 380.0866. **[α]_D_^20^** = -34 (*c* = 1.0, CHCl_3_), HPLC chiralcel AD-H column (25% isopropanol in hexanes, 0.5 mL/min, λ = 254 nm), t_R_ = 12.2 min (minor), 14.0 min (major), 97% *ee*.

**methyl (R,E)-4-(4,4*-d*ifluoro-3-(4-methylbenzamido)but-1-en-1-yl)benzoate (3v)**

The product **3v** was purified with silica gel chromatography (PE: EA = 8:1) as a white solid (64% yield, 45.9 mg). ^1^H NMR (500 MHz, Chloroform*-d*) δ 7.98 (d, *J =* 8.4 Hz, 2H), 7.74 (d, *J =* 8.2 Hz, 2H), 7.43 (d, *J =* 8.4 Hz, 2H), 7.26 (d, *J =* 8.0 Hz, 2H), 6.79 (dd, *J =* 16.0, 1.4 Hz, 1H), 6.54 (d, *J =* 8.8 Hz, 1H), 6.37 (dd, *J =* 16.0, 6.7 Hz, 1H), 6.02 (ddd, *J =* 56.9, 55.0, 2.2 Hz, 1H), 5.42 – 5.17 (m, 1H), 3.90 (s, 3H), 2.41 (s, 3H) ppm. ^13^C NMR (126 MHz, Chloroform*-d*) δ 167.27, 166.87, 142.95, 140.23, 134.59, 130.58, 130.10, 129.86, 129.54, 127.29, 126.73, 122.97, 114.46 (t, *J =* 245.9 Hz), 53.31 (dd, *J =* 24.9, 21.5 Hz), 52.30, 21.64 ppm. ^19^F NMR (471 MHz, Chloroform*-d*) δ -124.85 (ddd, *J =* 284.1, 55.3, 11.1 Hz), -128.81 (ddd, *J =* 283.7, 56.8, 19.2 Hz) ppm. HRMS (ESI) (*m/z*): [M+H]^+^ calcd. for C_20_H_20_F_2_NO_3_: 360.1406, found: 360.1429. **[α]_D_^20^** = -25 (*c* = 1.0, CHCl_3_), HPLC chiralcel AD-H column (40% isopropanol in hexanes, 0.5 mL/min, λ = 254 nm), t_R_ = 10.6min (minor), 13.7 min (major), 97% *ee*.

**methyl (R,E)-4-(4,4*-d*ifluoro-3-(3-methylbenzamido)but-1-en-1-yl)benzoate (3w)**

The product **3w** was purified with silica gel chromatography (PE: EA = 8:1) as a white solid (60% yield, 43.1 mg). ^1^H NMR (500 MHz, Chloroform*-d*) δ 7.98 (d, *J =* 8.0 Hz, 2H), 7.75 – 7.61 (m, 2H), 7.44 (d, *J =* 8.0 Hz, 2H), 7.35 (d, *J =* 5.6 Hz, 2H), 6.80 (d, *J =* 16.0 Hz, 1H), 6.55 (d, *J =* 8.8 Hz, 1H), 6.38 (dd, *J =* 16.0, 6.7 Hz, 1H), 6.20 – 5.80 (m, 1H), 5.28 (dq, *J =* 17.7, 8.8 Hz, 1H), 3.90 (s, 3H), 2.40 (s, 3H) ppm. ^13^C NMR (126 MHz, Chloroform*-d*) δ 167.53, 166.86, 140.21, 138.84, 134.65, 133.44, 133.09, 130.10, 129.89, 128.77, 127.99, 126.74, 124.24, 122.94, 114.44 (t, *J =* 246.0 Hz), 53.35 (dd, *J =* 24.8, 21.4 Hz), 52.31, 21.47 ppm. ^19^F NMR (471 MHz, Chloroform*-d*) δ -124.94 (ddd, *J =* 283.9, 55.7, 11.4 Hz), -128.71 (ddd, *J =* 283.8, 56.9, 19.2 Hz) ppm. HRMS (ESI) (*m/z*): [M+H]^+^ calcd. for C_20_H_20_F_2_NO_3_: 360.1406, found: 360.1416. **[α]_D_^20^** = -29 (*c* = 1.0, CHCl_3_), HPLC chiralcel AD-H column (40% isopropanol in hexanes, 0.5 mL/min, λ = 254 nm), t_R_ = 8.9 min (minor), 10.0 min (major), 98% *ee.*

**methyl (R,E)-4-(3-([1,1'-biphenyl]-4-carboxamido)-4,4*-d*ifluorobut-1-en-1-yl)benzoate (3x)**

The product **3x** was purified with silica gel chromatography (PE: EA = 8:1) as a white solid (60% yield, 50.5 mg). ^1^H NMR (500 MHz, Chloroform*-d*) δ 8.03 – 7.96 (m, 2H), 7.95 – 7.86 (m, 2H), 7.69 (d, *J =* 8.4 Hz, 2H), 7.63 – 7.59 (m, 2H), 7.49 – 7.43 (m, 4H), 7.43 – 7.38 (m, 1H), 6.83 (dd, *J =* 16.0, 1.4 Hz, 1H), 6.59 (d, *J =* 8.8 Hz, 1H), 6.40 (dd, *J =* 16.0, 6.7 Hz, 1H), 6.06 (ddd, *J =* 56.7, 54.8, 2.1 Hz, 1H), 5.31 (dq, *J =* 13.0, 8.7 Hz, 1H), 3.91 (s, 3H) ppm. ^13^C NMR (126 MHz, CDCl3) δ 167.04, 166.86, 145.19, 140.19, 139.88, 134.75, 132.04, 130.13, 129.93, 129.11, 128.33, 127.84, 127.54, 127.36, 126.76, 122.91, 114.44 (t, *J =* 246.0 Hz), 53.41 (dd, *J =* 24.6, 21.8 Hz), 52.32 ppm. ^19^F NMR (471 MHz, Chloroform*-d*) δ -125.00 (ddd, *J =* 283.8, 54.8, 10.5 Hz), -128.63 (ddd, *J =* 283.7, 56.5, 18.7 Hz) ppm. HRMS (ESI) (*m/z*): [M+H]^+^ calcd. for C_25_H_21_F_2_NO_3_: 422.1562, found: 422.1585. **[α]_D_^20^** = -17 (*c* = 1.0, CHCl_3_), HPLC chiralcel AD-H column (25% isopropanol in hexanes, 0.5 mL/min, λ = 254 nm), t_R_ = 28.1 min (minor), 44.7 min (major), 97% *ee*.

**methyl (R,E)-4-(3-(4-(tert-butyl)benzamido)-4,4*-d*ifluorobut-1-en-1-yl)benzoate (3y)**

The product **3y** was purified with silica gel chromatography (PE: EA = 8:1) as a white solid (59% yield, 47.3 mg). ^1^H NMR (400 MHz, Chloroform*-d*) δ 8.08 – 7.85 (m, 2H), 7.85 – 7.74 (m, 2H), 7.56 – 7.43 (m, 2H), 7.40 (d, *J =* 8.1 Hz, 2H), 6.95 – 6.64 (m, 2H), 6.37 (dd, *J =* 16.0, 6.6 Hz, 1H), 6.01 (ddd, *J =* 56.9, 54.9, 2.2 Hz, 1H), 5.53 – 5.11 (m, 1H), 3.89 (s, 3H), 1.32 (s, 9H) ppm. ^13^C NMR (101 MHz, Chloroform*-d*) δ 167.29, 166.87, 155.94, 140.25, 134.42, 130.55, 130.06, 129.78, 127.19, 126.69, 125.78, 123.09, 114.48 (t, *J =* 246.0 Hz), 53.30 (dd, *J =* 24.7, 21.7 Hz), 52.27, 35.10, 31.21 ppm. ^19^F NMR (376 MHz, Chloroform*-d*) δ -124.17 – -125.35 (m), -127.43 – -130.50 (m) ppm. HRMS (ESI) (*m/z*): [M+H]^+^ calcd. for C_23_H_26_F_2_NO_3_: 402.1875, found: 402.1842. **[α]_D_^20^** = -31 (*c* = 1.0, CHCl_3_), HPLC chiralcel AD-H column (40% isopropanol in hexanes, 0.5 mL/min, λ = 254 nm), t_R_ = 12.2 min (minor), 15.6 min (major), 97% *ee.*

**methyl (R,E)-4-(4,4*-d*ifluoro-3-(4-methoxybenzamido)but-1-en-1-yl)benzoate (3z)**

The product **3z** was purified with silica gel chromatography (PE: EA = 8:1) as a white solid (52% yield, 39.0 mg).^1^ H NMR (500 MHz, Chloroform*-d*) δ 7.98 (d, *J =* 8.4 Hz, 2H), 7.81 (d, *J =* 8.8 Hz, 2H), 7.43 (d, *J =* 8.4 Hz, 2H), 6.94 (d, *J =* 8.8 Hz, 2H), 6.79 (dd, *J =* 16.1, 1.5 Hz, 1H), 6.46 (d, *J =* 8.7 Hz, 1H), 6.37 (dd, *J =* 16.0, 6.7 Hz, 1H), 6.02 (ddd, *J =* 56.8, 54.9, 2.1 Hz, 1H), 5.27 (ddd, *J =* 16.9, 12.8, 8.5 Hz, 1H), 3.91 (s, 3H), 3.85 (s, 3H) ppm. ^13^C NMR (126 MHz, Chloroform*-d*) δ 166.87, 166.78, 162.85, 140.24, 134.55, 130.09, 129.85, 129.19, 126.73, 125.63, 123.08, 114.50 (t, *J =* 245.9 Hz), 114.05, 55.60, 53.29 (dd, *J =* 24.8, 21.6 Hz), 52.31 ppm. ^19^F NMR (471 MHz, Chloroform*-d*) δ -124.81 (ddd, *J =* 283.7, 55.4, 11.2 Hz), -128.88 (ddd, *J =* 283.6, 56.9, 19.5 Hz) ppm. HRMS (ESI) (*m/z*): [M+Na]^+^ calcd. for C_20_H_19_F_2_NNaO_4_: 398.1174, found: 398.1173. **[α]_D_^20^** =-37(*c* = 1.0, CHCl_3_), HPLC chiralcel AD-H column (25% isopropanol in hexanes, 0.5 mL/min, λ = 269 nm), t_R_ = 21.8 min (minor), 31.4 min (major), 98% *ee*.

**methyl (R,E)-4-(4,4*-d*ifluoro-3-(3-methoxybenzamido)but-1-en-1-yl)benzoate (3aa)**

The product **3aa** was purified with silica gel chromatography (PE: EA = 8:1) as a white solid (59% yield, 44.2 mg). ^1^H NMR (500 MHz, Chloroform*-d*) δ 8.00 – 7.84 (m, 2H), 7.41 (dd, *J =* 2.7, 1.6 Hz, 1H), 7.38 (dd, *J =* 8.4, 1.8 Hz, 3H), 7.33 (t, *J =* 7.9 Hz, 1H), 7.06 (ddd, *J =* 8.1, 2.6, 1.1 Hz, 1H), 6.83 (d, *J =* 8.8 Hz, 1H), 6.76 (dd, *J =* 16.0, 1.4 Hz, 1H), 6.34 (dd, *J =* 16.0, 6.8 Hz, 1H), 6.00 (ddd, *J =* 56.9, 54.9, 2.3 Hz, 1H), 5.34 – 5.14 (m, 1H), 3.89 (s, 3H), 3.81 (s, 3H) ppm. ^13^C NMR (126 MHz, Chloroform*-d*) δ 167.30, 166.86, 159.96, 140.17, 134.89, 134.57, 130.03, 129.82, 126.68, 122.89, 122.86, 119.04, 118.33, 114.40 (t, *J =* 246.1 Hz), 112.75, 55.52, 53.47 (dd, *J =* 24.8, 21.6 Hz), 52.27 ppm. ^19^F NMR (471 MHz, Chloroform*-d*) δ -124.81 (ddd, *J =* 283.6, 55.2, 10.6 Hz), -128.37 (ddd, *J =* 283.8, 56.6, 18.7 Hz) ppm. HRMS (ESI) (*m/z*): [M+H]^+^ calcd. for C_20_H_19_F_2_NNaO_4_: 398.1174, found: 398.1190. **[α]_D_^20^** = -21 (*c* = 1.0, CHCl_3_), HPLC chiralcel AD-H column (40% isopropanol in hexanes, 0.5 mL/min, λ = 254 nm), t_R_ = 11.0 min (major), 12.7 min (minor), 98% *ee*.

**methyl (R,E)-4-(4,4*-d*ifluoro-3-(4-(trifluoromethoxy)benzamido)but-1-en-1-yl)benzoate (3ab)**

The product **3ab** was purified with silica gel chromatography (PE: EA = 8:1) as a white solid (62% yield, 53.2 mg). ^1^H NMR (400 MHz, Chloroform*-d*) δ 7.89 (d, *J =* 8.4 Hz, 2H), 7.83 (d, *J =* 8.8 Hz, 2H), 7.33 (d, *J =* 8.4 Hz, 2H), 7.25 – 7.15 (m, 2H), 6.71 (dd, *J =* 16.1, 1.4 Hz, 1H), 6.67 (d, *J =* 8.8 Hz, 1H), 6.27 (dd, *J =* 16.0, 6.8 Hz, 1H), 5.95 (ddd, *J =* 56.6, 54.9, 2.2 Hz, 1H), 5.19 (dddd, *J =* 17.5, 10.5, 8.9, 6.9 Hz, 1H), 3.82 (s, 3H) ppm. ^13^C NMR (101 MHz, Chloroform*-d*) δ 166.89, 166.15, 152.07, 140.07, 134.87, 131.89, 130.11, 129.95, 129.32, 126.73, 122.69, 120.85, 120.39 (q, *J =* 258.6 Hz), 114.34 (t, *J =* 246.1 Hz), 53.57 (dd, *J =* 24.2, 21.8 Hz), 52.33 ppm. ^19^F NMR (376 MHz, Chloroform*-d*) δ -57.73, -125.31 (ddd, *J =* 283.7, 54.9, 11.4 Hz), -128.20 (ddd, *J =* 283.8, 56.4, 17.7 Hz) ppm. HRMS (ESI) (m/z): [M+H] ^+^ calcd. for C_20_H_17_F_5_NO_4_: 430.1072, found: 430.1074. [**[α]_D_^20^** = -14 (*c* = 1.0, CHCl_3_), HPLC chiralcel AD-H column (40% isopropanol in hexanes, 0.5 mL/min, λ = 254 nm), t_R_ = 8.9 min (minor), 10.9 min (major), 95% *ee*.

**methyl (R,E)-4-(3-(2-naphthamido)-4,4*-d*ifluorobut-1-en-1-yl)benzoate (3ac)**

The product **3ac** was purified with silica gel chromatography (PE: EA = 8:1) as a colorless oil (54% yield, 42.7 mg). ^1^H NMR (500 MHz, Chloroform*-d*) δ 8.36 (d, *J =* 1.7 Hz, 1H), 7.97 (d, *J =* 8.4 Hz, 2H), 7.93 – 7.82 (m, 4H), 7.56 (dddd, *J =* 21.2, 8.1, 6.8, 1.3 Hz, 2H), 7.43 (d, *J =* 8.3 Hz, 2H), 6.94 – 6.64 (m, 2H), 6.40 (dd, *J =* 16.0, 6.9 Hz, 1H), 6.07 (ddd, *J =* 56.8, 54.8, 2.2 Hz, 1H), 5.48 – 5.17 (m, 1H), 3.90 (s, 3H) ppm. ^13^C NMR (126 MHz, Chloroform*-d*) δ 167.49, 166.87, 140.19, 135.12, 134.76, 132.63, 130.64, 130.09, 129.88, 129.11, 128.84, 128.19, 128.03, 127.92, 127.12, 126.74, 123.59, 122.92, 114.38 (t, *J =* 246.1 Hz), 53.44 (dd, *J =* 24.4, 21.7 Hz), 52.30 ppm. ^19^F NMR (471 MHz, Chloroform*-d*) δ -124.88 (ddd, *J =* 283.8, 55.1, 11.4 Hz), -128.49 (ddd, *J =* 283.9, 56.8, 19.1 Hz) ppm. HRMS (ESI) (*m/z*): [M+H]^+^ calcd. for C_23_H_20_F_2_NO_3_: 396.1406, found: 396.1414. **[α]_D_^20^** = -20 (*c* = 1.0, CHCl_3_), HPLC chiralcel AD-H column (40% isopropanol in hexanes, 0.5 mL/min, λ = 238 nm), t_R_ = 15.0 min (minor), 24.0 min (major), 93% *ee*.

**methyl (R,E)-4-(3-(1-naphthamido)-4,4*-d*ifluorobut-1-en-1-yl)benzoate (3ad)**

The product **3ad** was purified with silica gel chromatography (PE: EA = 8:1) as a colorless oil (53% yield, 41.9 mg). ^1^H NMR (400 MHz, Chloroform*-d*) δ 8.36 – 8.24 (m, 1H), 7.97 (dd, *J =* 15.0, 8.3 Hz, 3H), 7.89 (dd, *J =* 7.0, 2.4 Hz, 1H), 7.68 (dd, *J =* 7.1, 1.2 Hz, 1H), 7.55 (ddt, *J =* 10.0, 6.9, 3.5 Hz, 2H), 7.51 – 7.40 (m, 3H), 6.86 (d, *J =* 16.4 Hz, 1H), 6.46 (d, *J =* 8.9 Hz, 1H), 6.37 (dd, *J =* 16.0, 6.8 Hz, 1H), 6.10 (ddd, *J =* 56.5, 54.9, 2.2 Hz, 1H), 5.37 (tt, *J =* 17.5, 8.5 Hz, 1H), 3.91 (s, 3H) ppm. ^13^C NMR (101 MHz, Chloroform*-d*) δ 169.41, 166.84, 140.13, 134.79, 133.83, 133.32, 131.47, 130.14, 129.98, 128.58, 127.62, 126.80, 126.77, 125.40, 125.22, 124.79, 122.83, 122.78, 114.41 (t, *J =* 246.2 Hz), 53.44 (dd, *J =* 23.9, 21.7 Hz), 52.32 ppm. ^19^F NMR (376 MHz, Chloroform*-d*) δ -125.51 (ddd, *J =* 283.6, 55.2, 12.4 Hz), -127.84 (ddd, *J =* 283.4, 56.5, 17.4 Hz) ppm. HRMS (ESI) (*m/z*): [M+H]^+^ calcd. for C_23_H_20_F_2_NO_3_: 396.1406, found: 396.1416. **[α]_D_^20^** = -11 (*c* = 1.0, CHCl_3_), HPLC chiralcel AD-H column (40% isopropanol in hexanes, 0.5 mL/min, λ = 254 nm), t_R_ = 11.3 min (major), 13.7 min (minor), 90% *ee*.

**methyl (R,E)-4-(4,4*-d*ifluoro-3-(1-oxoisoindolin-2-yl)but-1-en-1-yl)benzoate (3ae)**

The product **3ae** was purified with silica gel chromatography (PE: EA = 8:1) as a colorless oil (70% yield, 49.9 mg). ^1^H NMR (400 MHz, Chloroform*-d*) δ 8.05 – 7.97 (m, 2H), 7.89 (d, *J =* 7.5 Hz, 1H), 7.57 (td, *J =* 7.5, 1.2 Hz, 1H), 7.50 (d, *J =* 7.6 Hz, 1H), 7.46 (dd, *J =* 7.5, 4.5 Hz, 3H), 6.77 (d, *J =* 16.0 Hz, 1H), 6.51 (dd, *J =* 16.0, 7.4 Hz, 1H), 6.13 (ddd, *J =* 56.1, 54.7, 2.8 Hz, 1H), 5.39 (dddd, *J =* 19.0, 11.1, 7.8, 2.4 Hz, 1H), 4.56 (d, *J =* 2.9 Hz, 2H), 3.90 (s, 3H) ppm. ^13^C NMR (101 MHz, Chloroform*-d*) δ 169.03, 166.73, 141.79, 140.01, 136.48, 132.13, 131.52, 130.10, 130.07, 128.38, 126.75, 124.16, 123.04, 121.07, 114.87 (t, *J =* 247.2 Hz), 55.39 (dd, *J =* 25.1, 22.0 Hz), 52.27, 48.20 ppm. ^19^F NMR (376 MHz, Chloroform*-d*) δ -121.49 (ddd, *J =* 286.1, 54.7, 11.8 Hz), -125.66 (ddd, *J =* 286.0, 56.1, 18.3 Hz) ppm. HRMS (ESI) (*m/z*): [M+H]^+^ calcd. for C_20_H_18_F_2_NO_3_: 358.1249, found: 358.1237. **[α]_D_^20^** = -29 (*c* = 1.0, CHCl_3_), HPLC chiralcel AD-H column (40% isopropanol in hexanes, 0.5 mL/min, λ = 275 nm), t_R_ = 22.2 min (minor), 26.2 min (major), 87% *ee.*

**(R,E)-4-(3-benzamido-4,4*-d*ifluorobut-1-en-1-yl)phenyl 2-(11-oxo-6,11*-d*ihydrodibenzo[b,e]oxepin-3-yl)acetate (3af)**

The product **3af** was purified with silica gel chromatography (PE: EA = 3:1) as a white solid (64% yield, 70.8 mg). ^1^H NMR (500 MHz, Chloroform*-d*) δ 8.21 (d, *J =* 2.4 Hz, 1H), 7.89 (dd, *J =* 7.8, 1.3 Hz, 1H), 7.86 – 7.78 (m, 2H), 7.57 – 7.44 (m, 6H), 7.37 (d, *J =* 8.4 Hz, 3H), 7.06 (t, *J =* 8.8 Hz, 3H), 6.80 – 6.70 (m, 1H), 6.55 (d, *J =* 8.8 Hz, 1H), 6.20 (dd, *J =* 16.0, 6.9 Hz, 1H), 6.01 (ddd, *J =* 56.8, 54.9, 2.1 Hz, 1H), 5.25 (dd, *J =* 18.1, 9.0 Hz, 1H), 5.19 (s, 2H), 3.87 (s, 2H) ppm. ^13^C NMR (126 MHz, Chloroform*-d*) δ 190.93, 169.83, 167.23, 160.70, 150.66, 140.41, 136.35, 135.51, 134.66, 133.59, 133.47, 132.91, 132.64, 132.17, 129.51, 129.35, 128.77, 127.89, 127.71, 127.17, 127.07, 125.23, 121.75, 121.33, 120.33, 114.39 (t, *J =* 245.8 Hz), 73.65, 53.40 (dd, *J =* 24.7, 21.4 Hz), 40.26 ppm. ^19^F NMR (471 MHz, Chloroform*-d*) δ -125.28 (ddd, *J =* 283.5, 55.5, 11.5 Hz), -128.64 (ddd, *J =* 283.3, 56.9, 18.8 Hz) ppm. HRMS (ESI) (*m/z*): [M+Na]^+^ calcd. for C_33_H_25_F_2_NNaO_5_: 576.1593, found: 576.1595. **[α]_D_^20^** = -32(*c* = 1.0, CHCl_3_), HPLC chiralcel AD-H column (40% isopropanol in hexanes, 0.5 mL/min, λ = 281 nm), t_R_ = 62.3 min (minor), 76.4 min (major), 95% *ee*.

**(R,E)-4-(3-benzamido-4,4*-d*ifluorobut-1-en-1-yl)phenyl 2-(4-(4-chlorobenzoyl)phenoxy)-2-methylpropanoate (3ag)**

The product **3ag** was purified with silica gel chromatography (PE: EA = 5:1) as a white solid (62% yield, 74.8 mg). ^1^H NMR (500 MHz, Chloroform*-d*) δ 7.91 – 7.78 (m, 2H), 7.61 – 7.52 (m, 1H), 7.47 (dd, *J =* 8.4, 7.0 Hz, 2H), 7.43 – 7.35 (m, 2H), 7.26 – 7.22 (m, 2H), 7.02 – 6.95 (m, 2H), 6.94 – 6.86 (m, 2H), 6.76 (dd, *J =* 16.1, 1.4 Hz, 1H), 6.45 (d, *J =* 8.7 Hz, 1H), 6.22 (dd, *J =* 16.0, 6.9 Hz, 1H), 6.02 (ddd, *J =* 56.8, 54.9, 2.1 Hz, 1H), 5.37 – 5.13 (m, 1H), 1.73 (s, 6H) ppm. ^13^C NMR (126 MHz, Chloroform*-d*) δ 172.71, 167.31, 154.08, 150.61, 134.69, 134.01, 133.58, 132.32, 129.45, 128.92, 127.94, 127.74, 127.26, 121.64, 120.66, 115.76, 114.48 (t, *J =* 245.9 Hz), 79.72, 53.44 (dd, *J =* 24.7, 21.6 Hz), 25.46 ppm. ^19^F NMR (471 MHz, Chloroform*-d*) δ -125.35 (ddd, *J =* 283.6, 54.9, 11.5 Hz), -128.69 (ddd, *J =* 283.7, 57.1, 19.0 Hz) ppm. HRMS (ESI) (*m/z*): [M+Na]^+^ calcd. for C_34_H_28_ClF_2_NNaO_5_: 626.1516, found: 626.1505. **[α]_D_^20^** = -27 (*c* = 1.0, CHCl_3_), HPLC chiralcel AD-H column (40% isopropanol in hexanes, 0.5 mL/min, λ = 239 nm), t_R_ = 22.7 min (minor), 35.8 min (major), 97% *ee*.

**4-((R,E)-3-benzamido-4,4*-d*ifluorobut-1-en-1-yl)phenyl (S)-2-(naphthalen-2-yl)propanoate (3ah)**

The product **3ah** was purified with silica gel chromatography (PE: EA = 7:1) as a white solid (65% yield, 63.0 mg). ^1^H NMR (500 MHz, Chloroform*-d*) δ 7.86 – 7.80 (m, 2H), 7.80 – 7.67 (m, 3H), 7.56 – 7.48 (m, 2H), 7.45 (t, *J =* 7.6 Hz, 2H), 7.38 – 7.30 (m, 2H), 7.22 – 7.11 (m, 2H), 6.95 (d, *J =* 8.6 Hz, 2H), 6.72 (d, *J =* 16.0 Hz, 1H), 6.49 (d, *J =* 8.7 Hz, 1H), 6.17 (dd, *J =* 16.0, 6.9 Hz, 1H), 6.00 (ddd, *J =* 56.8, 54.9, 2.1 Hz, 1H), 5.22 (dq, *J =* 17.9, 8.8 Hz, 1H), 4.09 (q, *J =* 7.1 Hz, 1H), 3.92 (s, 3H), 1.69 (d, *J =* 7.1 Hz, 3H) ppm. ^13^C NMR (126 MHz, Chloroform*-d*) δ 173.20, 167.29, 157.91, 150.98, 135.13, 134.81, 133.97, 133.61, 133.55, 132.24, 129.45, 129.12, 128.86, 127.73, 127.54, 127.25, 126.29, 126.20, 121.81, 120.32, 120.28, 119.27, 114.49 (t, *J =* 245.9 Hz), 105.76, 55.45, 53.44 (dd, *J =* 24.6, 21.5 Hz), 45.70, 18.61 ppm. ^19^F NMR (471 MHz, Chloroform*-d*) δ -125.35 (ddd, *J =* 283.4, 55.1, 11.0 Hz), -128.61 (ddd, *J =* 283.2, 56.7, 18.3 Hz) ppm. HRMS (ESI) (*m/z*): [M+H]^+^ calcd. for C_30_H_26_F_2_NO_3_: 486.1875, found:486.1902. **[α]_D_^20^** = -41 (*c* = 1.0, CHCl_3_), HPLC chiralcel AD-H column (40% isopropanol in hexanes, 0.5 mL/min, λ = 254 nm), t_R_ = 29.8 min (minor), 42.2 min (major), 98% *de.*

**(R,E)-4-(3-benzamido-4,4*-d*ifluorobut-1-en-1-yl)phenyl 5-(2,5*-d*imethylphenoxy)-2,2*-d*imethylpentanoate (3ai)**

The product **3ai** was purified with silica gel chromatography (PE: EA = 5:1) as a white solid (58% yield, 62.1 mg). ^1^H NMR (500 MHz, Chloroform*-d*) δ 7.93 – 7.76 (m, 2H), 7.59 – 7.51 (m, 1H), 7.47 (dd, *J =* 8.4, 7.0 Hz, 2H), 7.42 – 7.36 (m, 2H), 7.07 – 6.95 (m, 3H), 6.78 – 6.75 (m, 1H), 6.68 (dd, *J =* 7.5, 1.6 Hz, 1H), 6.64 (d, *J =* 1.6 Hz, 1H), 6.52 (d, *J =* 8.7 Hz, 1H), 6.21 (dd, *J =* 16.0, 6.9 Hz, 1H), 6.02 (ddd, *J =* 56.8, 54.9, 2.1 Hz, 1H), 5.33 – 5.17 (m, 1H), 4.06 – 3.94 (m, 2H), 2.31 (s, 3H), 2.18 (s, 3H), 1.97 – 1.85 (m, 4H), 1.38 (s, 6H) ppm. ^13^C NMR (126 MHz, Chloroform*-d*) δ 176.38, 167.30, 156.97, 151.18, 136.61, 134.85, 133.63, 133.46, 132.25, 130.48, 128.87, 127.78, 127.27, 123.72, 121.94, 120.89, 120.27, 114.51 (t, *J =* 245.8 Hz), 112.07, 67.86, 53.46 (dd, *J =* 24.6, 21.3 Hz), 42.59, 37.24, 25.38, 25.24, 21.52, 15.91 ppm. ^19^F NMR (471 MHz, Chloroform*-d*) δ -125.34 (ddd, *J =* 283.2, 55.0, 11.5 Hz), -128.58 (ddd, *J =* 283.1, 56.6, 18.4 Hz) ppm. HRMS (ESI) (*m/z*): [M+H]^+^ calcd. for C_32_H_36_F_2_NO_4_: 536.2607, found: 536.2620. **[α]_D_^20^** = -33 (*c* = 1.0, CHCl_3_), HPLC chiralcel AD-H column (40% isopropanol in hexanes, 0.5 mL/min, λ = 254 nm), t_R_ = 11.7 min (minor), 15.7 min (major), 97% ee.

**(R,E)-4-(3-benzamido-4,4*-d*ifluorobut-1-en-1-yl)phenyl 2-(4-chlorophenoxy)-2-methylpropanoate (3aj)**

The product **3aj** was purified with silica gel chromatography (PE: EA = 5:1) as a white solid (63% yield, 62.9 mg). ^1^H NMR (500 MHz, Chloroform*-d*) δ 7.91 – 7.78 (m, 2H), 7.61 – 7.52 (m, 1H), 7.47 (dd, *J =* 8.4, 7.0 Hz, 2H), 7.43 – 7.35 (m, 2H), 7.26 – 7.22 (m, 2H), 7.02 – 6.95 (m, 2H), 6.94 – 6.86 (m, 2H), 6.76 (dd, *J =* 16.1, 1.4 Hz, 1H), 6.45 (d, *J =* 8.7 Hz, 1H), 6.22 (dd, *J =* 16.0, 6.9 Hz, 1H), 6.02 (ddd, *J =* 56.8, 54.9, 2.1 Hz, 1H), 5.37 – 5.13 (m, 1H), 1.73 (s, 6H) ppm. ^13^C NMR (126 MHz, Chloroform*-d*) δ 172.71, 167.31, 154.08, 150.61, 134.69, 134.01, 133.58, 132.32, 129.45, 128.92, 127.94, 127.74, 127.26, 121.64, 120.66, 115.76, 114.48 (t, *J =* 245.9 Hz), 79.72, 53.44 (dd, *J =* 24.7, 21.6 Hz), 25.46 ppm. ^19^F NMR (471 MHz, Chloroform*-d*) δ -125.35 (ddd, *J =* 283.6, 54.9, 11.5 Hz), -128.69 (ddd, *J =* 283.7, 57.1, 19.0 Hz) ppm. HRMS (ESI) (*m/z*): [M+H]^+^ calcd. for C_27_H_25_ClF_2_NO_4_: 500.1435, found: 500.1448. **[α]_D_^20^** = -42 (*c* = 1.0, CHCl_3_), HPLC chiralcel AD-H column (15% isopropanol in hexanes, 0.5 mL/min, λ = 254 nm), t_R_ = 74.2 min (minor), 78.5 min (major), 97% ee.

**4-((R,E)-3-benzamido-4,4*-d*ifluorobut-1-en-1-yl)phenyl (R)-2-(4-isobutylphenyl)propanoate (3ak)**

The product **3ak** was purified with silica gel chromatography (PE: EA = 8:1) as a white solid (62% yield, 60.9 mg). ^1^H NMR (500 MHz, Chloroform*-d*) δ 7.82 (d, *J =* 7.6 Hz, 2H), 7.54 (t, *J =* 7.4 Hz, 1H), 7.45 (t, *J =* 7.6 Hz, 2H), 7.34 (d, *J =* 8.2 Hz, 2H), 7.30 (d, *J =* 7.7 Hz, 2H), 7.15 (d, *J =* 7.7 Hz, 2H), 6.96 (d, *J =* 8.2 Hz, 2H), 6.73 (d, *J =* 16.0 Hz, 1H), 6.54 (d, *J =* 8.8 Hz, 1H), 6.18 (dd, *J =* 16.0, 6.9 Hz, 1H), 6.00 (t, *J =* 55.8 Hz, 1H), 5.23 (dq, *J =* 17.7, 8.7 Hz, 1H), 3.93 (q, *J =* 7.1 Hz, 1H), 2.47 (d, *J =* 7.1 Hz, 2H), 1.87 (dt, *J =* 13.5, 6.7 Hz, 1H), 1.60 (d, *J =* 7.1 Hz, 3H), 0.91 (d, *J =* 6.6 Hz, 6H) ppm. ^13^C NMR (126 MHz, Chloroform*-d*) δ 173.28, 167.30, 150.97, 141.01, 137.20, 134.79, 133.56, 133.48, 132.24, 129.66, 128.85, 127.71, 127.31, 127.26, 121.81, 120.24, 113.32 (t, *J =* 245.8 Hz), 52.26 (dd, *J =* 24.7, 21.4 Hz), 45.36, 45.14, 30.32, 22.51, 18.63 ppm. ^19^F NMR (471 MHz, Chloroform*-d*) δ -125.31 (ddd, *J =* 283.2, 55.1, 11.1 Hz), -128.60 (ddd, *J =* 283.2, 56.5, 18.2 Hz) ppm. HRMS (ESI) (*m/z*): [M+Na]^+^ calcd. for C_30_H_31_F_2_NNaO_3_: 514.2164, found: 514.2161. **[α]_D_^20^** = -38(*c* = 1.0, CHCl_3_), HPLC chiralcel AD-H column (25% isopropanol in hexanes, 0.5 mL/min, λ = 273 nm), t_R_ = 21.9 min (minor), 30.8min (major), 98% *de*.

**methyl (R,E)-4-(3-benzamido-4,4,4-trifluorobut-1-en-1-yl)benzoate (5a)**

The product **5a** was purified with silica gel chromatography (PE: EA = 8:1) as a white solid (70% yield, 50.8 mg). ^1^H NMR (500 MHz, Chloroform*-d*) δ 8.00 (d, *J =* 8.3 Hz, 2H), 7.91 – 7.81 (m, 2H), 7.60 – 7.53 (m, 1H), 7.52 – 7.41 (m, 4H), 6.94 – 6.76 (m, 1H), 6.54 (d, *J =* 9.6 Hz, 1H), 6.32 (dd, *J =* 16.0, 6.4 Hz, 1H), 5.64 (hept, *J =* 7.4 Hz, 1H), 3.91 (s, 3H) ppm. ^13^C NMR (126 MHz, Chloroform*-d*) δ 167.05, 166.79, 139.72, 135.33, 133.23, 132.56, 130.26, 130.17, 128.99, 127.35, 126.88, 124.58 (d, *J =* 281.3 Hz), 121.82, 52.94 (q, *J =* 31.6 Hz), 52.34 ppm. ^19^F NMR (471 MHz, Chloroform*-d*) δ -74.94 (d, *J =* 8.5 Hz) ppm. HRMS (ESI) (*m/z*): [M+H]^+^ calcd. for C_19_H_17_F_3_NO_3_: 364.1155, found: 364.1151. **[α]_D_^20^** = -38 (*c* = 1.0, CH_3_CN), HPLC chiralcel AD-H column (10% isopropanol in hexanes, 0.5 mL/min, λ = 275 nm), t_R_ = 38.5 min (major), 42.3 min (major), 93% *ee.*

**methyl (R,E)-3-(3-benzamido-4,4,4-trifluorobut-1-en-1-yl)benzoate (5b)**

The product **5b** was purified with silica gel chromatography (PE: EA = 8:1) as a white solid (65% yield, 47.2 mg). ^1^H NMR (500 MHz, Chloroform*-d*) δ 8.05 (d, *J =* 1.9 Hz, 1H), 7.94 (dt, *J =* 7.6, 1.5 Hz, 1H), 7.88 – 7.83 (m, 2H), 7.61 – 7.51 (m, 2H), 7.46 (t, *J =* 7.7 Hz, 2H), 7.39 (t, *J =* 7.7 Hz, 1H), 6.83 (dd, *J =* 15.9, 1.3 Hz, 1H), 6.72 – 6.60 (m, 1H), 6.29 (dd, *J =* 15.9, 6.6 Hz, 1H), 5.76 – 5.50 (m, 1H), 3.92 (s, 3H) ppm. ^13^C NMR (126 MHz, Chloroform*-d*) δ 167.13, 166.87, 135.73, 135.30, 133.27, 132.49, 131.33, 130.79, 129.76, 128.96, 128.93, 127.83, 127.37, 124.51 (q, *J =* 281.5 Hz), 120.55, 52.84 (q, *J =* 31.7 Hz), 52.39 ppm. ^19^F NMR (471 MHz, Chloroform*-d*) δ -74.98 (d, *J =* 8.1 Hz) ppm. HRMS (ESI) (*m/z*): [M+H]^+^ calcd. for C_19_H_17_F_3_NO_3_: 364.1155, found: 364.1151. **[α]_D_^20^** = -34 (*c* = 1.0, CH_3_CN), HPLC chiralcel AD-H column (10% isopropanol in hexanes, 0.5 mL/min, λ = 228 nm), t_R_ = 29.3 min (major), 66.3 min (minor), 91% *ee*.

**(R,E)-N-(4-(4-cyanophenyl)-1,1,1-trifluorobut-3-en-2-yl)benzamide (5c)**

The product **5c** was purified with silica gel chromatography (PE: EA = 6:1) as a colorless oil (50% yield, 33.0 mg). ^1^H NMR (500 MHz, DMSO*-d_6_*) δ 9.23 (d, *J =* 8.8 Hz, 1H), 7.90 (d, *J =* 7.6 Hz, 2H), 7.80 (d, *J =* 8.1 Hz, 2H), 7.66 (d, *J =* 8.2 Hz, 2H), 7.47 (t, *J =* 7.6 Hz, 2H), 7.01 (d, *J =* 16.0 Hz, 1H), 6.55 (dd, *J =* 16.0, 7.2 Hz, 1H), 5.61 (h, *J =* 8.1 Hz, 1H) ppm. ^13^C NMR (126 MHz, DMSO*-d_6_*) δ 166.65, 140.05, 134.26, 133.23, 132.80, 132.03, 128.47, 127.78, 127.54, 124.85 (q, *J =* 281.9 Hz) 123.47, 118.78, 110.77, 52.36 (q, *J =* 30.9 Hz) ppm. ^19^F NMR (471 MHz, DMSO*-d_6_*) δ -73.23 (d, *J =* 8.7 Hz) ppm. HRMS (ESI) (*m/z*): [M+H]^+^ calcd. for C_18_H_14_F_3_N_2_O: 331.1053, found: 331.1055. **[α]_D_^20^** = -18 (*c* = 1.0, CH_3_CN), HPLC chiralcel AD-H column (15% isopropanol in hexanes, 0.5 mL/min, λ = 254 nm), t_R_ = 52.6 min (major), 62.3min (minor), 91% *ee*.

**(R,E)-N-(4-(3-cyanophenyl)-1,1,1-trifluorobut-3-en-2-yl)benzamide (5d)**

The product **5d** was purified with silica gel chromatography (PE: EA = 8:1) as a white solid (51% yield, 36.3 mg). ^1^H NMR (500 MHz, DMSO*-d_6_*) δ 9.23 (d, *J =* 8.8 Hz, 1H), 8.00 (d, *J =* 1.8 Hz, 1H), 7.94 – 7.90 (m, 2H), 7.83 (d, *J =* 7.9 Hz, 1H), 7.75 (dd, *J =* 7.4, 4.1 Hz, 1H), 7.56 (dt, *J =* 7.8, 3.9 Hz, 2H), 7.49 (t, *J =* 7.6 Hz, 2H), 6.97 (d, *J =* 16.0 Hz, 1H), 6.55 (dd, *J =* 16.0, 7.1 Hz, 1H), 5.60 (q, *J =* 8.1 Hz, 1H) ppm. ^13^C NMR (126 MHz, DMSO*-d_6_*) δ 166.66, 136.74, 133.63, 133.25, 132.02, 131.95, 131.28, 130.35, 130.08, 128.45, 127.79, 124.87 (q, *J =* 283.0 Hz), 122.32, 118.58, 112.01, 52.31 (dd, *J =* 63.1, 31.7 Hz) ppm. ^19^F NMR (471 MHz, DMSO*-d_6_*) δ -73.23 (d, *J =* 8.7 Hz) ppm. HRMS (ESI) (*m/z*): [M+Na]^+^ calcd. for C_18_H_13_F_3_N_2_NaO: 353.0872, found: 353.0891. **[α]_D_^20^** = -11(*c* = 1.0, CH_3_CN), HPLC chiralcel AD-H column (15% isopropanol in hexanes, 0.5 mL/min, λ = 235 nm), t_R_ = 20.3 min (minor), 32.3 min (major), 91% *ee*.

**(R,E)-3-methyl-N-(1,1,1-trifluoro-4-(4-(trifluoromethyl)phenyl)but-3-en-2-yl)benzamide (5e)**

The product **5e** was purified with silica gel chromatography (PE: EA = 10:1) as a white solid (53% yield, 41.0 mg). ^1^H NMR (500 MHz, DMSO*-d_6_*) δ 9.14 (d, *J =* 8.8 Hz, 1H), 7.68 (m, 1H), 7.65 (m, 1H), 7.62 (m, 4H), 7.29 (d, *J =* 4.7 Hz, 2H), 6.96 (d, *J =* 16.0 Hz, 1H), 6.47 (dd, *J =* 16.0, 7.4 Hz, 1H), 5.56 (h, *J =* 8.1 Hz, 1H), 2.28 (s, 3H) ppm. ^13^C NMR (126 MHz, DMSO*-d_6_*) δ 166.75, 139.52, 137.84, 134.40, 133.30, 132.57, 128.79, 128.37, 128.23, 127.42, 125.74 (q, *J =* 3.8 Hz), 124.99, 124.94 (q, *J =* 282.3 Hz), 124.09 (q, *J =* 237.9 Hz), 122.68, 52.43 (q, *J =* 31.2 Hz), 20.91 ppm. ^19^F NMR (471 MHz, DMSO*-d_6_*) δ -61.19, -73.40 (d, *J =* 9.0 Hz) ppm. HRMS (ESI) (*m/z*): [M+H]^+^ calcd. for C_19_H_16_F_6_NO: 388.1131, found: 388.1141. **[α]_D_^20^** = -10(*c* = 1.0, CH_3_CN), HPLC chiralcel AD-H column (10% isopropanol in hexanes, 0.5 mL/min, λ = 248 nm), t_R_ = 24.7 min (minor), 26.9 min (major), 86% *ee*.

**methyl (R,E)-4-(3-benzamido-4,4,4-trifluorobut-1-en-1-yl)benzoate (5f)**

The product **5f** was purified with silica gel chromatography (PE: EA = 10:1) as a white solid (50% yield, 32.3 mg). ^1^H NMR (400 MHz, Chloroform*-d*) δ 7.91 – 7.74 (m, 2H), 7.56 (t, *J =* 7.4 Hz, 1H), 7.48 (t, *J =* 7.6 Hz, 2H), 7.36 (dd, *J =* 8.5, 5.4 Hz, 2H), 7.02 (t, *J =* 8.6 Hz, 2H), 6.77 (d, *J =* 15.9 Hz, 1H), 6.49 (d, *J =* 9.6 Hz, 1H), 6.12 (dd, *J =* 15.9, 6.7 Hz, 1H), 5.59 (q, *J =* 7.9 Hz, 1H) ppm. ^13^C NMR (101 MHz, Chloroform*-d*) δ 167.02, 163.10 (d, *J =* 248.7 Hz), 135.27, 133.35, 132.49, 131.62 (d, *J =* 3.5 Hz), 128.97, 128.62 (d, *J =* 8.3 Hz), 127.33, 124.68 (q, *J =* 281.4 Hz), 118.92, 115.88 (d, *J =* 21.8 Hz), 53.00 (q, *J =* 31.4 Hz) ppm. ^19^F NMR (376 MHz, Chloroform*-d*) δ -75.13 (d, *J =* 7.6 Hz), -112.46 ppm. HRMS (ESI) (*m/z*): [M+H]^+^ calcd. for C_17_H_14_F_4_NO: 324.1006, found: 324.1010. **[α]_D_^20^** = -8 (*c* = 1.0, CH_3_CN), HPLC chiralcel AD-H column (10% isopropanol in hexanes, 0.5 mL/min, λ = 263 nm), t_R_ = 20.2 min (major), 27.0 min (major), 86% *ee.*

**ethyl (R,E)-4-(3-benzamido-4,4,4-trifluorobut-1-en-1-yl)benzoate (5g)**

The product **5g** was purified with silica gel chromatography (PE: EA = 8:1) as a white solid (64% yield, 48.3 mg). ^1^H NMR (600 MHz, Chloroform*-d*) δ 7.98 (dd, *J =* 8.3, 3.1 Hz, 2H), 7.86 (dd, *J =* 8.0, 3.0 Hz, 2H), 7.65 – 7.53 (m, 1H), 7.47 (dt, *J =* 8.5, 4.2 Hz, 2H), 7.41 (dd, *J =* 8.3, 3.1 Hz, 2H), 6.83 (dd, *J =* 15.8, 3.1 Hz, 1H), 6.69 (dd, *J =* 9.7, 3.0 Hz, 1H), 6.31 (ddd, *J =* 15.9, 6.6, 3.0 Hz, 1H), 5.64 (q, *J =* 8.0 Hz, 1H), 4.36 (dd, *J =* 7.3, 3.1 Hz, 2H), 1.38 (td, *J =* 7.1, 2.8 Hz, 3H) ppm. ^13^C NMR (151 MHz, Chloroform*-d*) δ 167.14, 166.36, 139.60, 135.31, 133.19, 132.54, 130.51, 130.09, 128.94, 127.38, 126.80, 124.57 (q, *J =* 281.5 Hz), 121.67, 61.26, 52.91 (q, *J =* 31.6 Hz), 14.42 ppm. ^19^F NMR (565 MHz, Chloroform*-d*) δ -74.81 (d, *J =* 10.0 Hz) ppm. HRMS (ESI) (*m/z*): [M+H]^+^ calcd. for C_20_H_19_F_3_NO_3_: 378.1312, found: 378.1312. **[α]_D_^20^** = -11 (*c* = 1.0, CH_3_CN), HPLC chiralcel AD-H column (10% isopropanol in hexanes, 0.5 mL/min, λ = 281 nm), t_R_ = 33.4 min (major), 36.2 min (major), 90% *ee.*

**tert-butyl (R,E)-4-(3-benzamido-4,4,4-trifluorobut-1-en-1-yl)benzoate (5h)**

The product **5h** was purified with silica gel chromatography (PE: EA = 6:1) as a colorless oil (60% yield, 48.6 mg). ^1^H NMR (600 MHz, Chloroform*-d*) δ 7.89 (dd, *J =* 30.5, 7.8 Hz, 4H), 7.54 (t, *J =* 7.7 Hz, 1H), 7.44 (t, *J =* 7.6 Hz, 2H), 7.37 (d, *J =* 8.1 Hz, 2H), 6.96 (d, *J =* 9.8 Hz, 2H), 6.81 (d, *J =* 15.9 Hz, 1H), 6.29 (dd, *J =* 15.9, 6.5 Hz, 1H), 5.62 (h, *J =* 7.3 Hz, 1H), 1.58 (s, 9H) ppm. ^13^C NMR (126 MHz, Chloroform*-d*) δ 167.19, 165.49, 139.23, 135.42, 133.25, 132.49, 132.09, 129.95, 128.91, 127.40, 126.67, 124.60 (q, *J =* 281.6 Hz), 121.41, 81.40, 52.96 (q, *J =* 31.7 Hz), 28.28 ppm. ^19^F NMR (565 MHz, Chloroform*-d*) δ -74.75 (d, *J =* 11.1 Hz) ppm. HRMS (ESI) (*m/z*): [M+H]^+^ calcd. for C_22_H_23_F_3_NO_3_: 406.1625, found: 406.1628. **[α]_D_^20^** = -14 (*c* = 1.0, CH_3_CN), HPLC chiralcel AD-H column (10% isopropanol in hexanes, 0.5 mL/min, λ = 272 nm), t_R_ = 25.5 min (major), 32.8min (minor), 90% *ee*.

**methyl (R,E)-4-(3-benzamido-4,4,4-trifluorobut-1-en-1-yl)-2-methylbenzoate (5i)**

The product **5i** was purified with silica gel chromatography (PE: EA = 6:1) as a white solid (61% yield, 46.0 mg). ^1^H NMR (600 MHz, Chloroform*-d*) δ 7.86 (t, *J =* 8.2 Hz, 3H), 7.56 (t, *J =* 7.4 Hz, 1H), 7.46 (t, *J =* 7.6 Hz, 2H), 7.23 (d, *J =* 8.7 Hz, 2H), 6.77 (d, *J =* 15.9 Hz, 1H), 6.71 (d, *J =* 9.5 Hz, 1H), 6.29 (dd, *J =* 15.9, 6.4 Hz, 1H), 5.62 (h, *J =* 7.5 Hz, 1H), 3.87 (s, 3H), 2.57 (s, 3H) ppm. ^13^C NMR (151 MHz, Chloroform*-d*) δ 167.74, 167.14, 140.90, 138.67, 135.24, 133.19, 132.51, 131.27, 130.27, 129.48, 128.92, 127.37, 124.58 (q, *J =* 281.8 Hz), 124.00, 121.37, 52.88 (q, *J =* 31.5 Hz), 52.03, 21.92 ppm. ^19^F NMR (565 MHz, Chloroform*-d*) δ -74.83 (d, *J =* 12.7 Hz) ppm. HRMS (ESI) (*m/z*): [M+H]^+^ calcd. for C_20_H_19_F_3_NO_3_: 378.1312, found: 378.1315. **[α]_D_^20^** = -15 (*c* = 1.0, CH_3_CN), HPLC chiralcel AD-H column (15% isopropanol in hexanes, 0.5 mL/min, λ = 271 nm), t_R_ = 35.2 min (major), 59.6 min (minor), 93% *ee*.

**methyl (R,E)-4-(4,4,4-trifluoro-3-(3-methylbenzamido)but-1-en-1-yl)benzoate (5j)**

The product **5j** was purified with silica gel chromatography (PE: EA = 8:1) as a white solid (69% yield, 52.0 mg). ^1^H NMR (500 MHz, Chloroform*-d*) δ 8.01 – 7.87 (m, 2H), 7.72 – 7.53 (m, 2H), 7.39 (d, *J =* 8.4 Hz, 2H), 7.36 – 7.30 (m, 2H), 6.89 – 6.84 (m, 1H), 6.81 (dd, *J =* 16.0, 1.5 Hz, 1H), 6.30 (dd, *J =* 15.9, 6.5 Hz, 1H), 5.70 – 5.57 (m, 1H), 3.89 (s, 3H), 2.37 (s, 3H) ppm. ^13^C NMR (126 MHz, Chloroform*-d*) δ 167.43, 166.82, 139.78, 138.84, 135.17, 133.21, 133.17, 130.09, 128.74, 128.11, 126.81, 124.59 (q, *J =* 281.6 Hz), 124.36, 121.87, 52.91 (q, *J =* 31.7 Hz), 52.30, 21.39 ppm. ^19^F NMR (471 MHz, Chloroform*-d*) δ -74.86 (d, *J =* 7.9 Hz) ppm. [M+H]^+^ calcd. for C_20_H_19_F_3_NO_3_: 378.1312, found: 378.1313. **[α]_D_^20^** = -13 (*c* = 1.0, CH_3_CN), HPLC chiralcel AD-H column (10% isopropanol in hexanes, 0.5 mL/min, λ = 273 nm), t_R_ = 32.1 min (major), 34.6 min (minor), 94% *ee*.

**methyl (R,E)-4-(4,4,4-trifluoro-3-(4-methylbenzamido)but-1-en-1-yl)benzoate (5k)**

The product **5k** was purified with silica gel chromatography (PE: EA = 8:1) as a white solid (62% yield, 46.7 mg). ^1^H NMR (500 MHz, Chloroform*-d)* δ 7.98 (d, *J =* 8.1 Hz, 2H), 7.75 (d, *J =* 8.0 Hz, 2H), 7.42 (d, *J =* 8.1 Hz, 2H), 7.26 (d, *J =* 8.0 Hz, 2H), 6.82 (d, *J =* 15.9 Hz, 1H), 6.61 (d, *J =* 9.5 Hz, 1H), 6.31 (dd, *J =* 15.9, 6.4 Hz, 1H), 5.63 (q, *J =* 7.9 Hz, 1H), 3.91 (s, 3H), 2.41 (s, 3H) ppm. ^13^C NMR (126 MHz, Chloroform*-d*) δ 167.03, 166.80, 143.17, 139.78, 135.17, 130.35, 130.17, 130.13, 129.58, 127.38, 126.84, 124.61 (q, *J =* 281.6 Hz), 121.96, 52.87 (q, *J =* 31.5 Hz), 52.32, 21.64 ppm. ^19^F NMR (471 MHz, Chloroform*-d*) δ -74.92 (d, *J =* 7.8 Hz) ppm. HRMS (ESI) (*m/z*): [M+H]^+^ calcd. for C_20_H_19_F_3_NO_3_: 378.1312, found: 378.1306. **[α]_D_^20^** = -11 (*c* = 1.0, CH_3_CN), HPLC chiralcel AD-H column (8% isopropanol in hexanes, 0.5 mL/min, λ = 272 nm), t_R_ = 65.8 min (major), 70.8 min (minor), 91% *ee*.

**methyl (R,E)-4-(3-(3,5*-d*imethylbenzamido)-4,4,4-trifluorobut-1-en-1-yl)benzoate (5l)**

The product **5l** was purified with silica gel chromatography (PE: EA = 8:1) as a white solid (55% yield, 43.0 mg). ^1^H NMR (500 MHz, DMSO*-d_6_*) δ 9.09 (d, *J =* 8.8 Hz, 1H), 7.89 (d, *J =* 8.1 Hz, 2H), 7.58 (d, *J =* 8.1 Hz, 2H), 7.48 (m, 2H), 7.14 (s, 1H), 6.97 (d, *J =* 15.9 Hz, 1H), 6.48 (dd, *J =* 16.0, 7.5 Hz, 1H), 5.65 – 5.50 (m, 1H), 3.78 (s, 3H), 2.26 (s, 6H) ppm. ^13^C NMR (126 MHz, DMSO*-d_6_*) δ 166.79, 165.94, 140.09, 137.70, 134.80, 133.26, 129.78, 129.32, 127.02, 125.44, 125.31, 124.94 (d, *J =* 283.0 Hz), 122.54, 52.55 (q, *J =* 31.2 Hz), 52.25, 20.86 ppm. ^19^F NMR (471 MHz, DMSO*-d_6_*) δ -73.23 (d, *J =* 8.7 Hz) ppm. HRMS (ESI) (*m/z*): [M+H]^+^ calcd. for C_21_H_21_F_3_NO_3_: 392.1468, found: 392.1473. **[α]_D_^20^** = -10 (*c* = 1.0, CH_3_CN), HPLC chiralcel AD-H column (8% isopropanol in hexanes, 0.5 mL/min, λ = 254 nm), t_R_ = 27.0 min (minor), 33.5 min (major), 91% *ee*.

**methyl (R,E)-4-(3-(4-ethylbenzamido)-4,4,4-trifluorobut-1-en-1-yl)benzoate (5m)**

The product **5m** was purified with silica gel chromatography (PE: EA = 8:1) as a white solid (55% yield, 43.0 mg). ^1^H NMR (500 MHz, Chloroform*-d*) δ 7.90 (d, *J =* 8.0 Hz, 2H), 7.71 (d, *J =* 7.8 Hz, 2H), 7.33 (d, *J =* 8.0 Hz, 2H), 7.21 (d, *J =* 7.9 Hz, 2H), 6.74 (d, *J =* 16.0 Hz, 1H), 6.66 – 6.57 (m, 1H), 6.24 (dd, *J =* 15.8, 6.3 Hz, 1H), 5.68 – 5.50 (m, 1H), 3.83 (s, 3H), 2.62 (q, *J =* 7.5 Hz, 2H), 1.17 (t, *J =* 7.5 Hz, 3H) ppm. ^13^C NMR (126 MHz, Chloroform*-d*) δ 167.10, 166.82, 149.38, 139.77, 135.11, 130.53, 130.11, 128.40, 127.51, 126.83, 124.60 (q, *J =* 281.6 Hz), 121.94, 52.84 (q, *J =* 31.5 Hz), 52.33, 28.95, 15.42 ppm. ^19^F NMR (471 MHz, Chloroform*-d*) δ -74.91 (d, *J =* 9.8 Hz) ppm. HRMS (ESI) (*m/z*): [M+H]^+^ calcd. for C_21_H_21_F_3_NO_3_: 392.1468, found: 392.1476. **[α]_D_^20^** = -11 (*c* = 1.0, CH_3_CN), HPLC chiralcel AD-H column (8% isopropanol in hexanes, 0.5 mL/min, λ = 254 nm), t_R_ = 65.3 min (minor), 70.1 min (major), 94% *ee*.

**methyl (R,E)-4-(4,4,4-trifluoro-3-(4-isopropylbenzamido)but-1-en-1-yl)benzoate (5n)**

The product **5n** was purified with silica gel chromatography (PE: EA = 8:1) as a white solid (65% yield, 52.0 mg). ^1^H NMR (500 MHz, DMSO*-d_6_*) δ 9.12 (d, *J =* 8.8 Hz, 1H), 7.92 (d, *J =* 8.5 Hz, 2H), 7.84 (d, *J =* 8.4 Hz, 2H), 7.60 (d, *J =* 8.5 Hz, 2H), 7.33 (d, *J =* 8.3 Hz, 2H), 7.00 (d, *J =* 16.0 Hz, 1H), 6.51 (dd, *J =* 16.0, 7.4 Hz, 1H), 5.59 (d, *J =* 8.1 Hz, 1H), 3.81 (s, 3H), 2.97 – 2.87 (m, 1H), 1.17 (d, *J =* 7.0 Hz, 6H) ppm. ^13^C NMR (126 MHz, DMSO*-d_6_*) δ 166.45, 165.90, 152.73, 140.07, 134.68, 130.89, 129.74, 129.28, 127.91, 126.99, 126.37, 124.92 (q, *J =* 282.6 Hz), 122.54, 52.84 (q, *J =* 31.5 Hz), 52.21, 33.45, 23.65 ppm. ^19^F NMR (471 MHz, DMSO*-d_6_*) δ -73.33 (d, *J =* 9.1 Hz) ppm. HRMS (ESI) (*m/z*): [M+H]^+^ calcd. for C_22_H_23_F_3_NO_3_: 406.1625, found: 406.1632. **[α]_D_^20^** = -16 (*c* = 1.0, CH_3_CN), HPLC chiralcel AD-H column (8% isopropanol in hexanes, 0.5 mL/min, λ = 269 nm), t_R_ = 60.7 min (minor), 77.5 min (major), 95% *ee*.

**Methyl (R,E)-4-(4,4,4-trifluoro-3-(4-isobutylbenzamido)but-1-en-1-yl)benzoate (5o)**

The product **5o** was purified with silica gel chromatography (PE: EA = 8:1) as a white solid (70% yield, 58.7 mg). ^1^H NMR (500 MHz, DMSO*-d_6_*) δ 9.12 (d, *J =* 8.7 Hz, 1H), 7.91 (d, *J =* 8.1 Hz, 2H), 7.82 (d, *J =* 7.9 Hz, 2H), 7.59 (d, *J =* 8.2 Hz, 2H), 7.23 (d, *J =* 7.9 Hz, 2H), 6.99 (d, *J =* 16.0 Hz, 1H), 6.50 (dd, *J =* 16.0, 7.4 Hz, 1H), 5.59 (h, *J =* 8.1 Hz, 1H), 3.79 (s, 3H), 2.45 (s, 2H), 1.79 (dh, *J =* 11.8, 6.0, 5.4 Hz, 1H), 0.79 (d, *J =* 6.6 Hz, 6H) ppm. ^13^C NMR (126 MHz, DMSO*-d_6_*) δ 166.47, 165.89, 145.58, 140.07, 134.72, 130.79, 129.73, 129.29, 128.96, 127.69, 126.96, 124.93 (q, *J =* 282.1 Hz), 122.54, 52.40 (q, *J =* 30.9 Hz), 52.16, 44.37, 29.61, 22.07 ppm. ^19^F NMR (471 MHz, DMSO*-d*6) δ -73.36 (d, *J =* 9.2 Hz) ppm. HRMS (ESI) (*m/z*): [M+H]^+^ calcd. for C_23_H_25_F_3_NO_3_: 420.1781, found: 420.1790. **[α]_D_^20^** = -12 (*c* = 1.0, CH_3_CN), HPLC chiralcel AD-H column (8% isopropanol in hexanes, 0.5 mL/min, λ = 272 nm), t_R_ = 67.2 min (minor), 81.4 min (major), 94% *ee*.

**methyl (R,E)-4-(3-(4-(tert-butyl)benzamido)-4,4,4-trifluorobut-1-en-1-yl)benzoate (5p)**

The product **5p** was purified with silica gel chromatography (PE: EA = 8:1) as a white solid (70% yield, 56.5 mg). ^1^H NMR (500 MHz, DMSO*-d_6_*) δ 9.12 (d, *J =* 8.9 Hz, 1H), 7.91 (d, *J =* 8.0 Hz, 2H), 7.84 (d, *J =* 8.3 Hz, 2H), 7.59 (d, *J =* 8.1 Hz, 2H), 7.46 (d, *J =* 8.4 Hz, 2H), 6.98 (d, *J =* 16.0 Hz, 1H), 6.51 (dd, *J =* 16.0, 7.3 Hz, 1H), 5.60 (h, *J =* 8.5 Hz, 1H), 3.79 (s, 3H), 1.23 (s, 9H) ppm. ^13^C NMR (126 MHz, DMSO*-d_6_*) δ 166.44, 165.88, 154.87, 140.07, 134.65, 130.52, 129.73, 129.28, 127.65, 126.96, 126.05, 124.93 (q, *J =* 282.6 Hz), 122.54, 52.36 (q, *J =* 30.9 Hz), 52.16, 34.69, 30.90 ppm. ^19^F NMR (471 MHz, DMSO*-d*_6_) δ -73.36 (d, *J =* 8.8 Hz) ppm. HRMS (ESI) (*m/z*): [M+H]^+^ calcd. for C_23_H_25_F_3_NO_3_: 420.1781, found: 420.1786. **[α]_D_^20^** = -9 (*c* = 1.0, CH_3_CN), HPLC chiralcel AD-H column (8% isopropanol in hexanes, 0.5 mL/min, λ = 254 nm), t_R_ = 55.0 min (minor), 67.2 min (major), 95% *ee*.

**methyl (R,E)-4-(4,4,4-trifluoro-3-(4-methoxybenzamido)but-1-en-1-yl)benzoate (5q)**

The product **5p** was purified with silica gel chromatography (PE: EA = 6:1) as a white solid (65% yield, 51.0 mg). ^1^H NMR (500 MHz, DMSO*-d_6_*) δ 9.02 (d, *J =* 8.7 Hz, 1H), 7.89 (d, *J =* 8.2 Hz, 4H), 7.58 (s, 1H), 7.57 (s, 1H), 7.03 – 6.92 (m, 3H), 6.50 (dd, *J =* 16.0, 7.4 Hz, 1H), 5.58 (h, *J =* 8.2 Hz, 1H), 3.78 (s, 3H), 3.76 (s, 3H) ppm. ^13^C NMR (126 MHz, DMSO*-d_6_*)) δ 165.94, 165.89, 162.19, 140.07, 134.65, 129.72, 129.29, 126.94, 125.35, 124.96 (q, *J =* 282.7 Hz), 122.64, 113.64, 55.42, 52.37 (q, *J =* 30.8 Hz), 52.15 ppm. ^19^F NMR (471 MHz, DMSO*-d_6_*)) δ -73.34 (d, *J =* 9.5 Hz) ppm. HRMS (ESI) (*m/z*): [M+H]^+^ calcd. for C_20_H_19_F_3_NO_4_: 394.1621, found: 394.1620. **[α]_D_^20^** = -11 (*c* = 1.0, CH_3_CN), HPLC chiralcel AD-H column (10% isopropanol in hexanes, 0.5 mL/min, λ = 273 nm), t_R_ = 79.9 min (major), 85.0 min (minor), 91% *ee*.

**methyl (R,E)-4-(4,4,4-trifluoro-3-(3-methoxybenzamido)but-1-en-1-yl)benzoate (5r)**

The product **5r** was purified with silica gel chromatography (PE: EA = 6:1) as a white solid (58% yield, 45.6 mg). ^1^H NMR (500 MHz, DMSO*-d_6_*) δ 9.20 (d, *J =* 8.8 Hz, 1H), 7.94 (d, *J =* 8.4 Hz, 2H), 7.63 (d, *J =* 8.4 Hz, 2H), 7.51 (dt, *J =* 7.7, 1.3 Hz, 1H), 7.46 (dd, *J =* 2.6, 1.6 Hz, 1H), 7.40 (t, *J =* 7.9 Hz, 1H), 7.13 (ddd, *J =* 8.2, 2.7, 1.0 Hz, 1H), 7.02 (d, *J =* 15.9 Hz, 1H), 6.53 (dd, *J =* 16.0, 7.4 Hz, 1H), 5.61 (h, *J =* 8.1 Hz, 1H), 3.83 (s, 3H), 3.80 (s, 3H) ppm. ^13^C NMR (126 MHz, DMSO*-d_6_*) δ 166.27, 165.87, 159.23, 140.01, 134.78, 134.57, 129.72, 129.61, 129.29, 124.86 (q, *J =* 282.2 Hz), 123.74, 122.39, 119.97, 117.87, 112.79, 55.37, 52.44 (q, *J =* 31.2 Hz), 52.18 ppm. ^19^F NMR (471 MHz, DMSO*-d_6_*) δ -73.27 (d, *J =* 8.8 Hz) ppm. HRMS (ESI) (*m/z*): [M+Na]^+^ calcd. for C_20_H_18_F_3_NnaO_4_: 416.1080, found: 416.1089. **[α]_D_^20^** = -11 (*c* = 1.0, CH_3_CN), HPLC chiralcel AD-H column (8% isopropanol in hexanes, 0.5 mL/min, λ = 254 nm), t_R_ = 65.9 min (major), 70.3 min (minor), 90% *ee*.

**(1S,2R,5S)-2-isopropyl-5-methylcyclohexyl 4-((R,E)-3-benzamido-4,4,4-trifluorobut-1-en-1-yl)benzoate (5s)**

The product **5s** was purified with silica gel chromatography (PE: EA = 6:1) as a white solid (58% yield, 56.5 mg). ^1^H NMR (400 MHz, Chloroform*-d*) δ 7.99 (d, *J =* 8.2 Hz, 2H), 7.91 – 7.83 (m, 2H), 7.56 (t, *J =* 7.3 Hz, 1H), 7.48 (d, *J =* 7.6 Hz, 2H), 7.42 (d, *J =* 8.2 Hz, 2H), 6.84 (d, *J =* 15.9 Hz, 1H), 6.62 (d, *J =* 9.6 Hz, 1H), 6.31 (dd, *J =* 16.0, 6.5 Hz, 1H), 5.64 (q, *J =* 7.9 Hz, 1H), 4.91 (tt, *J =* 10.6, 5.3 Hz, 1H), 2.10 (dd, *J =* 10.3, 5.9 Hz, 1H), 1.93 (ddt, *J =* 9.9, 7.0, 2.9 Hz, 1H), 1.73 – 1.66 (m, 2H), 1.62 – 1.53 (m, 2H), 1.15 – 1.07 (m, 2H), 0.92 (dd, *J =* 6.8, 4.4 Hz, 7H), 0.78 (d, *J =* 7.0 Hz, 3H) ppm. ^13^C NMR (101 MHz, Chloroform*-d*) δ 167.09, 165.81, 139.54, 135.39, 133.24, 132.53, 130.12, 128.96, 127.37, 126.79, 124.69 (q, *J =* 261.0 Hz), 121.63, 75.19, 52.95 (q, *J =* 31.9 Hz), 47.37, 41.07, 34.42, 31.56, 26.67, 23.78, 22.17, 20.88, 16.68 ppm. ^19^F NMR (376 MHz, Chloroform*-d*) δ -74.94 (d, *J =* 8.2 Hz) ppm. HRMS (ESI) (*m/z*): [M+H]^+^ calcd. for C_28_H_33_F_3_NO_3_: 488.2407, found: 488.2405. **[α]_D_^20^** = -17 (*c* = 1.0, CH_3_CN), HPLC chiralcel AD-H column (10% isopropanol in hexanes, 0.5 mL/min, λ = 254 nm), t_R_ = 51.3 min (minor), 56.5 min (major), 91% *de*.

**2,2*-d*imethyl-3-(m-tolyl)propyl (R,E)-4-(3-benzamido-4,4,4-trifluorobut-1-en-1-yl)benzoate (5t)**

The product **5t** was purified with silica gel chromatography (PE: EA = 6:1) as a white solid (58% yield, 65.2 mg). ^1^H NMR (500 MHz, DMSO*-d_6_*) δ 9.15 (d, *J =* 8.6 Hz, 1H), 7.82 (dd, *J =* 17.4, 7.6 Hz, 4H), 7.52 (d, *J =* 7.9 Hz, 2H), 7.44 (d, *J =* 7.0 Hz, 1H), 7.37 (t, *J =* 7.5 Hz, 2H), 7.00 (t, *J =* 7.4 Hz, 1H), 6.91 (d, *J =* 16.0 Hz, 1H), 6.85 (d, *J =* 7.3 Hz, 1H), 6.82 – 6.75 (m, 2H), 6.42 (dd, *J =* 15.9, 7.3 Hz, 1H), 5.51 (q, *J =* 8.1 Hz, 1H), 3.79 (s, 2H), 2.36 (s, 3H), 2.08 (s, 3H), 0.80 (s, 6H) ppm. ^13^C NMR (126 MHz, DMSO*-d_6_*) δ 166.56, 165.22, 140.03, 137.68, 136.76, 134.76, 133.17, 131.92, 130.94, 129.64, 129.38, 128.38, 127.68, 127.29, 126.97, 126.68, 124.82 (q, *J =* 282.5 Hz), 122.33, 78.91, 71.61, 52.40 (q, *J =* 31.1 Hz), 44.35, 34.76, 24.05, 20.9 ppm. ^19^F NMR (471 MHz, DMSO*-d_6_*) δ -73.31 (d, *J =* 9.6 Hz) ppm. HRMS (ESI) (*m/z*): [M+H]^+^ calcd. for C_30_H_31_F_3_NO_3_: 510.2251, found: 510.2249. **[α]_D_^20^** = -15 (*c* = 1.0, CH_3_CN), HPLC chiralcel AD-H column (10% isopropanol in hexanes, 0.5 mL/min, λ = 254 nm), t_R_ = 28.1 min (major), 37.7 min (minor), 91% *ee*.

**(E)-3,7*-d*imethylocta-2,6*-d*ien-1-yl 4-((R,E)-3-benzamido-4,4,4-trifluorobut-1-en-1-yl)benzoate (5u)**

The product **5u** was purified with silica gel chromatography (PE: EA = 7:1) as a white solid (54% yield, 52.4 mg). ^1^H NMR (500 MHz, DMSO*-d_6_*) δ 9.18 (dd, *J =* 8.9, 3.7 Hz, 1H), 7.86 (dd, *J =* 8.4, 4.5 Hz, 4H), 7.54 (dd, *J =* 8.5, 3.5 Hz, 2H), 7.48 (dd, *J =* 7.2, 3.6 Hz, 1H), 7.41 (t, *J =* 7.4 Hz, 2H), 6.95 (dd, *J =* 16.0, 3.6 Hz, 1H), 6.47 (ddd, *J =* 16.0, 7.5, 3.7 Hz, 1H), 5.56 (q, *J =* 8.4 Hz, 1H), 5.31 (d, *J =* 7.3 Hz, 1H), 4.94 (t, *J =* 6.3 Hz, 1H), 4.69 (dd, *J =* 7.4, 3.7 Hz, 2H), 1.94 (dt, *J =* 9.3, 4.9 Hz, 4H), 1.62 (d, *J =* 3.7 Hz, 3H), 1.49 (s, 3H), 1.43 (d, *J =* 3.6 Hz, 3H) ppm. ^13^C NMR (126 MHz, DMSO*-d_6_*) δ 166.60, 165.31, 141.91, 140.00, 134.82, 133.29, 131.98, 131.08, 129.72, 129.59, 128.43, 127.78, 126.94, 124.92 (d, *J =* 282.2 Hz), 123.70, 122.39, 118.55, 118.47, 61.43, 52.49 (q, *J =* 30.9 Hz), 25.79, 25.44, 17.51, 16.21 ppm. ^19^F NMR (471 MHz, DMSO*-d_6_*) δ -73.38 (d, *J =* 8.8 Hz) ppm. HRMS (ESI) (*m/z*): [M+Na]^+^ calcd. for C_28_H_30_F_3_NNaO_3_: 508.2070, found: 508.2090. **[α]_D_^20^** = -12 (*c* = 1.0, CH_3_CN), HPLC chiralcel AD-H column (8% isopropanol in hexanes, 0.5 mL/min, λ = 210 nm), t_R_ = 32.9 min (major), 47.7 min (minor), 90% *ee*.

**(Z)-hex-3-en-1-yl 4-((R,E)-3-benzamido-4,4,4-trifluorobut-1-en-1-yl)benzoate (5v)**

The product **5v** was purified with silica gel chromatography (PE: EA = 7:1) as a white solid (63% yield, 54.3 mg). ^1^H NMR (400 MHz, Chloroform*-d*) δ 7.98 (d, *J =* 8.4 Hz, 2H), 7.90 – 7.82 (m, 2H), 7.60 – 7.53 (m, 1H), 7.47 (t, *J =* 7.7 Hz, 2H), 7.44 – 7.40 (m, 2H), 6.84 (dd, *J =* 15.9, 1.4 Hz, 1H), 6.64 (d, *J =* 9.5 Hz, 1H), 6.31 (dd, *J =* 16.0, 6.5 Hz, 1H), 5.69 – 5.60 (m, 1H), 5.53 (dtt, *J =* 10.5, 7.2, 1.6 Hz, 1H), 5.39 (dtt, *J =* 10.6, 7.3, 1.6 Hz, 1H), 4.30 (t, *J =* 6.9 Hz, 2H), 2.51 (qd, *J =* 7.0, 1.5 Hz, 2H), 2.09 (pd, *J =* 7.5, 1.5 Hz, 2H), 0.96 (t, *J =* 7.5 Hz, 3H) ppm. ^13^C NMR (101 MHz, Chloroform*-d*) δ 166.98, 166.20, 139.55, 135.21, 134.73, 133.10, 132.42, 130.34, 130.01, 128.83, 127.25, 126.71, 124.46 (q, *J =* 281.5 Hz), 123.64, 121.63, 64.63, 52.82 (q, *J =* 31.8 Hz), 26.82, 20.65, 14.25 ppm. ^19^F NMR (376 MHz, Chloroform*-d*) δ -74.92 (d, *J =* 8.0 Hz) ppm. HRMS (ESI) (*m/z*): [M+H]^+^ calcd. for C_24_H_25_F_3_NO_3_: 432.1781, found: 432.1783. **[α]_D_^20^** = -10 (*c* = 1.0, CH_3_CN), HPLC chiralcel AD-H column (8% isopropanol in hexanes, 0.5 mL/min, λ = 210 nm), t_R_ = 32.9 min (major), 47.7 min (minor), 91% *ee*.

**(S)-3,7*-d*imethyloct-6-en-1-yl 4-((R,E)-3-benzamido-4,4,4-trifluorobut-1-en-1-yl)benzoate (5w)**

The product **5w** was purified with silica gel chromatography (PE: EA = 7:1) as a white solid (60% yield, 58.4 mg). ^1^H NMR (500 MHz, DMSO*-d*_6_) δ 9.20 (d, *J* = 8.9 Hz, 1H), 7.88 (t, *J* = 8.1 Hz, 4H), 7.60 – 7.55 (m, 2H), 7.50 (dd, *J* = 7.1, 1.8 Hz, 1H), 7.44 (t, *J* = 7.7 Hz, 2H), 6.98 (d, *J* = 15.9 Hz, 1H), 6.49 (dd, *J* = 16.0, 7.3 Hz, 1H), 5.59 (h, *J* = 8.2 Hz, 1H), 4.97 (t, *J* = 7.1 Hz, 1H), 4.22 (qt, *J* = 11.2, 6.6 Hz, 2H), 1.86 (qt, *J* = 16.4, 8.3 Hz, 3H), 1.72 – 1.60 (m, 1H), 1.52 (s, 3H), 1.45 (s, 3H), 1.25 (ddt, *J* = 12.3, 9.2, 6.0 Hz, 1H), 1.15 – 1.03 (m, 2H), 0.83 (d, *J* = 6.4 Hz, 3H) ppm. ^13^C NMR (126 MHz, DMSO*-d*_6_) δ 166.57, 165.35, 140.01, 134.79, 133.28, 131.96, 130.62, 129.65, 129.57, 128.41, 127.77, 126.92, 124.90 (q, *J* = 282.5 Hz), 124.46, 122.38, 63.01, 52.47 (q, *J* = 31.0 Hz), 36.42, 34.92, 28.88, 25.45, 24.86, 19.28, 17.45 ppm. ^19^F NMR (471 MHz, DMSO*-d*_6_) δ -73.38 (d, *J* = 9.2 Hz) ppm. HRMS (ESI) (*m/z*): [M+H]^+^ calcd. for C_28_H_33_F_3_NO_3_: 488.2407, found: 488.2406. **[α]_D_^20^** = -15 (*c* = 1.0, CH_3_CN), HPLC chiralcel AD-H column (8% isopropanol in hexanes, 0.5 mL/min, λ = 210 nm), t_R_ = 30.5 min (major), 41.7 min (minor), 91% *ee*.

**Synthetic utility**

Scale-up reactions

NiBr_2_•DME (10 mol%, 0.1 mmol), **L9** (13 mol%, 0.13 mmol) were firstly combined in a 50 mL oven*-d*ried sealing tube. The vessel was evacuated and backfilled with Ar (repeated for 3 times), 10 mL THF was added via syringe and the complex was allowed to pre-stir at 25 ˚C for 30 min. Then CF_2_H-substituted secondary alkyl chloride **1a** (1.2 equiv, 1.2 mmol), vinyl iodides **2a** (1.0 equiv, 1.0 mmol), Mn podwer (2.0 equiv, 2.0 mmol ) and TMSCl (0.8 equiv, 0.8 mmol) were added and the tube was cooled to -20 ˚C. The tube was sealed with a Teflon lined cap and stired at -20 ˚C for 48 h. The reaction mixture was then diluted with EtOAc (~50 mL) and filtered through a pad of celite. The filtrate was added brine (50 mL) and extracted with EtOAc (3×50 mL), the combined organic layer was dried over Na_2_SO_4_, filtrated and concentrated under vacuum. The residue was then purified by flash column chromatography to give desired products **3a** (255.3 mg, 74% yield, 99% *ee*).

NiBr_2_•DME (10 mol%, 0.1 mmol), **L9** (13 mol%, 0.13 mmol) were firstly combined in a 50 mL oven*-d*ried sealing tube. The vessel was evacuated and backfilled with Ar (repeated for 3 times), 6 mLTHF was added via syringe and the complex was allowed to pre-stir at 25 ˚C for 30 min. Then CF_3_-substituted secondary alkyl chloride **4a** (1.4 equiv, 1.4 mmol), vinyl iodides **2a** (1.0 equiv, 1.0 mmol), Mn podwer (2.0 equiv, 2.0 mmol ) and TMSCl (0.8 equiv, 0.8 mmol) were added and the tube was cooled to -40 ˚C. The tube was sealed with a Teflon lined cap and stired at -40 ˚C for 48 h. The reaction mixture was then diluted with EtOAc (~50 mL) and filtered through a pad of celite. The filtrate was added brine (50 mL) and extracted with EtOAc (3×50 mL), the combined organic layer was dried over Na_2_SO_4_, filtrated and concentrated under vacuum. The residue was then purified by flash column chromatography to give desired products **5a**. (235.9 mg, 65% yield, 91% *ee*)

Products Derivatization

The chiral α-CF_2_H-substituted olefin **3a** (34.5 mg, 0.1 mmol, 1.0 equiv.), Pd/C (10 mg) was added to a 25 mL oven*-d*ried sealing tube. After that, the system was evacuated and a hydrogen-filled balloon was attached. The reaction was stirred at room temperature for 12 hours. The reaction mixture was then passed through a short pad of silica gel, with ethyl acetate as the eluent (~35 mL). The resulting mixture was concentrated, and the residue was purified by chromatography on silica gel to give the desired product **6a** (73% yield, 97% *ee*).

**methyl (R)-4-(3-benzamido-4,4*-d*ifluorobutyl)benzoate (6a)**

The product **6a** was purified with silica gel chromatography (PE: EA = 7:1) as a white solid (73% yield, 25.7 mg). ^1^H NMR (400 MHz, Chloroform*-d*) δ 7.96 – 7.87 (m, 2H), 7.77 – 7.64 (m, 2H), 7.56 – 7.49 (m, 1H), 7.43 (dd, *J* = 8.3, 6.9 Hz, 2H), 7.25 (d, *J* = 8.1 Hz, 2H), 6.23 (d, *J* = 9.2 Hz, 1H), 5.89 (ddd, *J* = 56.8, 55.1, 2.0 Hz, 1H), 4.69 – 4.38 (m, 1H), 3.88 (s, 3H), 2.90 – 2.71 (m, 2H), 2.24 – 2.09 (m, 1H), 1.95 (dddd, *J* = 14.5, 10.3, 8.6, 6.4 Hz, 1H) ppm. ^13^C NMR (126 MHz, CDCl_3_) δ 167.76, 167.09, 146.28, 133.56, 132.18, 130.06, 128.79, 128.50, 128.37, 127.15, 114.94 (t, *J* = 245.0 Hz), 52.16, 51.04 (dd, *J* = 24.7, 21.0 Hz), 32.03, 28.54 (d, *J* = 4.1 Hz) ppm. ^19^F NMR (376 MHz, Chloroform*-d*) δ -125.90 (ddd, *J* = 282.8, 55.4, 10.9 Hz), -130.32 (ddd, *J* = 283.0, 56.8, 19.1 Hz) ppm. HRMS (ESI) (*m/z*): [M+Na]^+^ calcd. for C_19_H_19_F_2_NNaO_3_: 370.1255, found: 370.1237. **[α]_D_^20^** = + 5 (*c* = 1.0, CHCl_3_), HPLC chiralcel OD-H column (20% isopropanol in hexanes, 0.5 mL/min, λ = 254 nm), t_R_ = 28.7 min (major), 35.5 min (minor), 97% *ee*.

The chiral α-CF_3_-substituted olefin **5a** (36.3 mg, 0.1 mmol, 1.0 equiv.), Pd/C (10 mg) was added to a 25 mL oven*-d*ried sealing tube. After that, the system was evacuated and a hydrogen-filled balloon was attached. The reaction was stirred at room temperature for 12 hours. The reaction mixture was then passed through a short pad of silica gel, with ethyl acetate as the eluent (~35 mL). The resulting mixture was concentrated, and the residue was purified by chromatography on silica gel to give the desired product **7a** (68% yield, 91% *ee*).

**methyl (R)-4-(3-benzamido-4,4,4-trifluorobutyl)benzoate (7a)**

The product **7a** was purified with silica gel chromatography (PE: EA = 7:1) as a white solid (68% yield, 26.0 mg). ^1^H NMR (400 MHz, DMSO*-d*_6_) δ 8.84 (d, *J* = 8.9 Hz, 1H), 7.83 (ddd, *J* = 14.6, 7.4, 1.5 Hz, 4H), 7.56 – 7.48 (m, 1H), 7.44 (dd, *J* = 8.2, 6.7 Hz, 2H), 7.30 (d, *J* = 8.1 Hz, 2H), 4.63 (dtt, *J* = 16.2, 8.2, 3.3 Hz, 1H), 3.76 (s, 3H), 2.73 (ddd, *J* = 14.3, 9.4, 5.2 Hz, 1H), 2.67 – 2.57 (m, 1H), 2.16 – 1.92 (m, 2H) ppm. ^13^C NMR (126 MHz, DMSO*-d*_6_) δ 167.18, 166.20, 146.46, 133.43, 131.88, 129.37, 128.85, 128.45, 127.66, 127.63, 125.68 (q, *J* = 282.5 Hz), 52.04, 49.73 (q, *J* = 29.7 Hz), 30.79, 27.91 ppm. ^19^F NMR (376 MHz, DMSO*-d*_6_) δ -74.47 (d, *J* = 8.4 Hz) ppm. HRMS (ESI) (*m/z*): [M+Na]^+^ calcd. for C_19_H_18_F_3_NNaO_3_: 388.1131, found: 388.1140. **[α]_D_^20^** = -3 (*c* = 1.0, CH_3_CN), HPLC chiralcel AD-H column (8% isopropanol in hexanes, 0.5 mL/min, λ = 254 nm), t_R_ = 37.6 min (minor), 82.6 min (major), 91% *ee*.

The chiral α-CF_2_H-substituted olefin **3e** (71.0 mg, 0.2 mmol, 1.0 equiv.), Pd/C (20 mg) was added to a 25 mL oven*-d*ried sealing tube. After that, the system was evacuated and a hydrogen-filled balloon was attached. The reaction was stirred at room temperature for 12 hours. The reaction mixture was then passed through a short pad of silica gel, with ethyl acetate as the eluent (~35 mL). The resulting mixture was concentrated to get crude **6b** without furthure purification to use in next step.

Borane-methyl sulfide complex (2 M in THF, 0.25 mL, 0.5 mmol, 2.5 equiv) was added to a solution of crude **6b** (1.0 equiv) in THF (2.0 mL) at 0 °C in a 4-mL vial under an argon atmosphere. The mixture was stirred at 80 °C for 12 h. After completion, 3.0 ml MeOH was added, and the mixture was concentrated, 5 ml 10% NaOH solution was added, the reaction mixture was extracted with Et_2_O (3 × 15 mL). The combined organic layers were dried over Na_2_SO_4_ and concentrated under reduced pressure. The residue was purified by flash column chromatography to provide **6e** (58% yield totally in two steps, 98% *ee*).

**(R)-N-benzyl-1,1*-d*ifluoro-4-(4-(trifluoromethyl)phenyl)butan-2-amine (6e)**

The product **6e** was purified with silica gel chromatography (PE: EA = 10:1) as a colorless oil (58% yield, 39.7 mg). ^1^H NMR (400 MHz, Chloroform*-d*) δ 7.52 (d, *J* = 8.0 Hz, 2H), 7.43 – 7.33 (m, 4H), 7.33 – 7.28 (m, 1H), 7.23 (d, *J* = 8.0 Hz, 2H), 5.84 (td, *J* = 56.1, 3.5 Hz, 1H), 3.99 (d, *J* = 13.2 Hz, 1H), 3.86 (d, *J* = 13.2 Hz, 1H), 2.87 (ddd, *J* = 14.5, 9.2, 5.6 Hz, 2H), 2.76 (ddd, *J* = 13.9, 9.1, 7.0 Hz, 1H), 1.96 (dddd, *J* = 13.9, 9.4, 7.0, 4.4 Hz, 1H), 1.79 (dtd, *J* = 14.4, 8.8, 5.7 Hz, 1H) ppm. ^13^C NMR (101 MHz, CDCl_3_) δ 145.49, 138.96, 128.88, 128.76, 128.63, 128.44, 127.72, 125.54 (q, *J* = 3.8 Hz), 124.41 (q, *J* = 271.9 Hz), 117.21 (t, *J* = 245.1 Hz), 57.29 (t, *J* = 21.2 Hz), 51.51, 31.61, 29.82 ppm. ^19^F NMR (376 MHz, Chloroform*-d*) δ -62.33, -125.41 (d, *J* = 58.1 Hz), -125.67 (dd, *J* = 56.7, 13.1 Hz) ppm. HRMS (ESI) (*m/z*): [M+H]^+^ calcd. for C_18_H_19_F_5_N: 344.1432, found: 344.1435. **[α]_D_^20^** = +1 (*c* = 1.0, CH_3_CN), HPLC chiralcel OD-H column (8% isopropanol in hexanes, 0.5 mL/min, λ = 214 nm), t_R_ = 12.8 min (major), 13.9 min (minor), 98% *ee*.

Mechanism Investigation

**1. Radical trapping experiment with TEMPO**

NiBr_2_•DME (10 mol%, 0.02 mmol), **L9** (13 mol%, 0.026 mmol) were firstly combined in a 25 mL oven*-d*ried sealing tube. The vessel was evacuated and backfilled with Ar (repeated for 3 times), 2 mLTHF was added via syringe and the complex was allowed to pre-stir at 25 ˚C for 30 min. Then CF_2_H-substituted secondary alkyl chloride **1** (1.2 equiv, 0.24 mmol), vinyl iodides **2** (1.0 equiv, 0.20 mmol), Mn podwer (2.0 equiv, 0.4 mmol ) , TMSCl (0.8 equiv, 0.16 mmol) and TEMPO (2.0 equiv, 0.4 mmol) were added and the tube was cooled to -20 ˚C. The tube was sealed with a Teflon lined cap and stired at -20 ˚C for 48 h. The reaction mixture was then diluted with EtOAc (~10 mL) and filtered through a pad of celite. The filtrate was added brine (20 mL) and extracted with EtOAc (3×15 mL), the combined organic layer was dried over Na_2_SO_4_, filtrated and concentrated under vacuum. The residue was then purified by flash column chromatography to give desired products. TEMPO-captured product **5n** was detected by HRMS (ESI) analysis.

NiBr_2_•DME (10 mol%, 0.02 mmol), **L9** (13 mol%, 0.026 mmol) were firstly combined in a 25 mL oven*-d*ried sealing tube. The vessel was evacuated and backfilled with Ar (repeated for 3 times), 1.2 mLTHF was added via syringe and the complex was allowed to pre-stir at 25 ˚C for 30 min. Then CF_3_-substituted secondary alkyl chloride **4** (1.4 equiv, 0.28 mmol), vinyl iodides **2** (1.0 equiv, 0.20 mmol), Mn podwer (2.0 equiv, 0.4 mmol ) , TMSCl (0.8 equiv, 0.16 mmol) and TEMPO (2.0 equiv, 0.4 mmol)were added and the tube was cooled to -40 ˚C. The tube was sealed with a Teflon lined cap and stired at -40 ˚C for 48 h. The reaction mixture was then diluted with EtOAc (~10 mL) and filtered through a pad of celite. The filtrate was added brine (20 mL) and extracted with EtOAc (3×15 mL), the combined organic layer was dried over Na_2_SO_4_, filtrated and concentrated under vacuum. The residue was then purified by flash column chromatography to give desired products. TEMPO-captured product **5n** was detected by HRMS (ESI) analysis.

**2. Control experiment**

The substrate **8** was synthesized according to the reported literature.^[3][4]^

NiBr_2_•DME (10 mol%, 0.02 mmol), **L9** (13 mol%, 0.026 mmol) were firstly combined in a 25 mL oven*-d*ried sealing tube. The vessel was evacuated and backfilled with Ar (repeated for 3 times), 1.2 mLTHF was added via syringe and the complex was allowed to pre-stir at 25 ˚C for 30 min. Then CF_3_-substituted secondary alkyl chloride **8** (1.4 equiv, 0.28 mmol), vinyl iodides **2a** (1.0 equiv, 0.20 mmol), Mn podwer (2.0 equiv, 0.4 mmol ) , TMSCl (0.8 equiv, 0.16 mmol) and TEMPO (2.0 equiv, 0.4 mmol)were added and the tube was cooled to -40 ˚C. The tube was sealed with a Teflon lined cap and stired at -40 ˚C for 48 h. The reaction mixture was then diluted with EtOAc (~10 mL) and filtered through a pad of celite. The filtrate was added brine (20 mL) and extracted with EtOAc (3×15 mL), the combined organic layer was dried over Na_2_SO_4_, filtrated and concentrated under vacuum.

Ni(COD)_2_ (10 mol%, 0.02 mmol), **L9** (13 mol%, 0.026 mmol) were firstly combined in a 25 mL oven*-d*ried sealing tube. The vessel was evacuated and backfilled with Ar (repeated for 3 times), 2 mLTHF was added via syringe and the complex was allowed to pre-stir at 25 ˚C for 30 min. Then CF_2_-substituted secondary alkyl chloride **1a** (1.2 equiv, 0.24 mmol), vinyl iodides **2a** (1.0 equiv, 0.20 mmol), Mn podwer (2.0 equiv, 0.4 mmol ) and TMSCl (0.8 equiv, 0.16 mmol) were added and the tube was cooled to -20 ˚C. The tube was sealed with a Teflon lined cap and stired at -20 ˚C for 48 h. The reaction mixture was then diluted with EtOAc (~10 mL) and filtered through a pad of celite. The filtrate was added brine (20 mL) and extracted with EtOAc (3×15 mL), the combined organic layer was dried over Na_2_SO_4_, filtrated and concentrated under vacuum. The residue was then purified by flash column chromatography to give desired products **3a** (36.5 mg, 53% yield, 97% *ee*).

Computational Details

**General computational methods.** All the calculations were performed using Gaussian 16 software packages.^[5]^ The geometry optimizations were performed with the (U)B3LYP^[6]^ hybrid functional. A mixed basis set of SDD^[7]^ for Ni and I, while 6-31G(d) ^[8]^for all the other atoms was used. Vibrational frequency analysis was calculated at the same level of theory to validate each structure as either minima (no imaginary frequencies) or transition states (one and only one imaginary frequency) on the potential energy surface. Intrinsic reaction coordinate (IRC) calculations^[9]^ were conducted to ensure the relevance of transition states to the corresponding intermediates. Exhaustive conformational searches were performed for all intermediates in order to map out the lowest energy profile. To obtain more accurate energies, solution-phase single point energy calculations were performed in in THF (using SMD solvation model at the (U)B3LYP-D3(BJ)/6-311+G(d,p)-SDD level. Molecular geometries were rendered with CYLview. ^[10]^

**L9Ni(II)RI**

C	-0.35041500	1.15517000	-0.11805900

C	-1.65187400	0.87800000	-0.83898300

C	-2.11886800	-0.55040300	-0.65642900

O	-0.08654200	2.46166400	0.07663000

C	1.35366400	2.54272400	0.35165500

C	1.66288900	1.10183800	0.87204100

N	0.56184600	0.32015200	0.24315000

N	-1.47595200	-1.56890500	-0.20810200

C	-2.33976900	-2.76539900	-0.43139700

C	-3.73859600	-2.10192700	-0.65092600

O	-3.36498000	-0.76632900	-1.12246300

C	-1.37200400	1.00299300	-2.41436800

H	-0.84517600	0.08683700	-2.69537800

H	-2.34890700	0.96159500	-2.90340300

C	-2.78072300	1.87637600	-0.39139600

H	-3.54197400	1.84924200	-1.16698600

H	-2.34942900	2.87953000	-0.42021900

C	-6.95141100	0.53001000	0.13529400

C	-5.63752200	0.93278000	-0.00710200

C	-4.80471200	1.19471100	1.11127700

C	-5.36960200	1.03663900	2.42048100

C	-6.71850000	0.62157800	2.52989000

C	-7.49932800	0.37168600	1.41849900

C	-3.42396200	1.64098100	0.97262400

C	-4.56574400	1.31277000	3.60314600

C	-3.22987800	1.76709800	3.42616200

C	-2.70832700	1.92436900	2.10260100

C	-2.43151900	2.06605900	4.55627900

H	-1.40984800	2.40209400	4.40191300

C	-2.92819300	1.91525900	5.83565700

C	-4.24727600	1.45830800	6.01836700

C	-5.04487200	1.16509700	4.92704400

H	-7.55492000	0.33465300	-0.74657400

H	-5.23936900	1.02488800	-1.00865800

H	-7.16210500	0.49897800	3.51119700

H	-8.53192900	0.05624300	1.53951900

H	-1.69795200	2.30901700	2.00833400

H	-2.30570900	2.14424200	6.69609000

H	-4.64339000	1.33350600	7.02238800

H	-6.05480000	0.81394200	5.10601300

C	-3.00989600	4.91850700	-3.65173800

C	-2.48211300	3.69534200	-3.28437600

C	-1.08766700	3.44880100	-3.29359200

C	-0.21420400	4.49895400	-3.72600200

C	-0.78604100	5.74117000	-4.08774800

C	-2.15089600	5.95590000	-4.04892900

C	-0.53116700	2.16146100	-2.91719300

C	1.22269600	4.26727000	-3.80300500

C	1.72175900	2.97111700	-3.49134200

C	0.81252900	1.95174900	-3.06347800

C	3.10946600	2.70600500	-3.58424800

H	3.46837400	1.70659800	-3.34912700

C	3.99538800	3.69512200	-3.96466600

C	3.50971000	4.98215700	-4.26543800

C	2.15602300	5.25888500	-4.18729600

H	-4.08514700	5.07255300	-3.63653800

H	-3.16115200	2.90240200	-2.99673400

H	-0.14464500	6.55084000	-4.41665400

H	-2.55598800	6.92192400	-4.33666700

H	1.23018400	0.97379300	-2.83088400

H	5.05841100	3.48241600	-4.03265800

H	4.20138800	5.76533700	-4.56318700

H	1.81889800	6.26161400	-4.42488300

Ni	0.50428000	-1.66477500	0.30922100

C	2.35884900	-1.87923900	0.07690600

C	3.44041400	-1.76906200	0.87058400

H	2.51462000	-2.14377700	-0.98042800

H	3.29111700	-1.55811700	1.92966000

C	4.85346400	-1.91082600	0.48242400

C	5.85236400	-1.67911600	1.45097300

C	5.28050100	-2.25742400	-0.81806200

C	7.20465800	-1.77711700	1.14508100

H	5.54874600	-1.41919800	2.46245700

C	6.62774300	-2.35489100	-1.13007100

H	4.54197400	-2.46130300	-1.58785700

C	7.61043000	-2.11309100	-0.15498700

H	7.95243400	-1.59517200	1.90945300

H	6.94916900	-2.62388200	-2.13143100

I	0.40854400	-3.93797100	1.53259900

C	9.03349700	-2.22803800	-0.55014400

O	9.42834800	-2.50051700	-1.66996000

O	9.88414600	-1.99187500	0.48352500

C	11.27498100	-2.09600700	0.16085100

H	11.54953200	-1.37306900	-0.61291900

H	11.80859800	-1.88385400	1.08860100

H	11.51573200	-3.10083000	-0.19799000

H	1.82111500	2.64919000	-0.63208300

H	2.60220900	0.75585000	0.43833400

C	1.74774700	0.91439200	2.37806500

C	2.94890600	1.25430500	3.01906700

C	0.69087400	0.41801100	3.14733200

C	3.08537800	1.11535000	4.39857700

H	3.78419400	1.62735800	2.43149800

C	0.82989400	0.26853800	4.53083200

H	-0.23650800	0.11696300	2.67206400

C	2.02343500	0.61989200	5.16021600

H	4.02348100	1.38343000	4.87702900

H	0.00196100	-0.13184200	5.10860200

H	2.13197200	0.49775000	6.23448300

C	1.69354100	3.74751700	1.18287300

C	2.80344400	4.51777500	0.81247700

C	0.95372900	4.12170600	2.31203400

C	3.18127900	5.63009000	1.56620400

H	3.37121700	4.24906700	-0.07547300

C	1.32056900	5.24197600	3.05575200

H	0.08785300	3.54021600	2.60821300

C	2.43836900	5.99580100	2.68909500

H	4.04638500	6.21545700	1.26737800

H	0.73403500	5.52519400	3.92536800

H	2.72389600	6.86655800	3.27295400

H	-4.20287500	-1.94349300	0.32927200

H	-2.34526800	-3.35277900	0.48772400

C	-4.73173400	-2.74808500	-1.57644100

C	-5.71994500	-3.57509800	-1.02839300

C	-4.69064500	-2.56247200	-2.96385700

C	-6.64084700	-4.22305200	-1.85245500

H	-5.77115600	-3.71040100	0.04966200

C	-5.61964900	-3.19920000	-3.78631200

H	-3.93478400	-1.91697600	-3.39800200

C	-6.59353900	-4.03471500	-3.23460900

H	-7.39964000	-4.86474700	-1.41318100

H	-5.58010400	-3.04488100	-4.86119500

H	-7.31397800	-4.53202300	-3.87827200

C	-1.84233300	-3.64888900	-1.56366700

C	-2.05842700	-5.03005500	-1.47935600

C	-1.21903300	-3.13694700	-2.70704500

C	-1.66880500	-5.87804000	-2.51473900

H	-2.52120500	-5.44543300	-0.58841500

C	-0.82043200	-3.98380400	-3.74347600

H	-1.02730500	-2.07191200	-2.79041700

C	-1.04628800	-5.35730200	-3.65121500

H	-1.83847100	-6.94754700	-2.42674000

H	-0.33145100	-3.56813300	-4.62071000

H	-0.73261400	-6.01823000	-4.45458400

**TS1-S**

C	-1.31391700	1.41374500	-0.10912000

C	-2.46280000	0.59424400	-0.65251200

C	-2.26867600	-0.89423400	-0.43748700

O	-1.60734600	2.71334000	0.08903500

C	-0.32280400	3.40675200	0.19052100

C	0.63893900	2.24395300	0.61148000

N	-0.09806100	1.05118200	0.10615000

N	-1.18553800	-1.57058100	-0.27875200

C	-1.57309500	-3.01899400	-0.34915800

C	-3.09704100	-2.93635500	-0.04609100

O	-3.41780400	-1.59328200	-0.53025900

C	-2.46425700	0.76634800	-2.25083600

H	-1.62930400	0.16311700	-2.61465200

H	-3.38255100	0.28765500	-2.60435900

C	-3.83523000	1.05719500	-0.04516600

H	-4.60304000	0.68366700	-0.71591600

H	-3.86728500	2.14632300	-0.12378800

C	-7.22236000	-1.56581100	0.99247600

C	-6.13491500	-0.76975500	0.68992800

C	-5.30464300	-0.21259600	1.69600800

C	-5.62518700	-0.50096800	3.06414300

C	-6.74656700	-1.31986900	3.33899700

C	-7.53407400	-1.84374400	2.33291400

C	-4.16652000	0.64550700	1.38780200

C	-4.81110100	0.04926000	4.13919900

C	-3.71273700	0.88530100	3.79920700

C	-3.43559100	1.16006300	2.42183900

C	-2.91411800	1.45169000	4.82162000

H	-2.07378800	2.08108900	4.54192900

C	-3.18101000	1.19754500	6.15222600

C	-4.26261300	0.36468300	6.49626200

C	-5.05730000	-0.19441100	5.51179600

H	-7.82830600	-1.97971400	0.19148200

H	-5.90993600	-0.59394900	-0.35369800

H	-7.00367300	-1.54617300	4.36733000

H	-8.38741000	-2.46913300	2.58009400

H	-2.61243400	1.83391600	2.21058800

H	-2.55964200	1.63437800	6.92904700

H	-4.47630500	0.15752500	7.54128900

H	-5.88110000	-0.83117000	5.81388300

C	-5.79719400	3.54720600	-3.12259900

C	-4.74647900	2.68315100	-2.87947900

C	-3.39798900	3.06914800	-3.07455900

C	-3.13532600	4.38699400	-3.57179600

C	-4.23224000	5.25035400	-3.80061900

C	-5.53607800	4.84972900	-3.57747200

C	-2.29211400	2.15922000	-2.83006400

C	-1.76644200	4.80015900	-3.85297500

C	-0.72087300	3.84983300	-3.68244100

C	-1.02628900	2.54510300	-3.17823400

C	0.61451800	4.20770000	-3.98787300

H	1.39562800	3.46121600	-3.86315600

C	0.92429500	5.47669100	-4.43664700

C	-0.10370600	6.42615200	-4.59280000

C	-1.41658800	6.09313000	-4.30905700

H	-6.81986500	3.21585000	-2.96593900

H	-4.96888500	1.67700700	-2.54715200

H	-4.05625000	6.25246800	-4.17483100

H	-6.35496100	5.53778600	-3.76759600

H	-0.20434700	1.84152800	-3.07149500

H	1.95201100	5.74161900	-4.66858300

H	0.13303300	7.42768700	-4.94119300

H	-2.18176000	6.85002300	-4.44048000

C	3.49228100	-3.98402800	-1.35617000

O	2.71635500	-4.87059700	-1.68726100

C	4.78765600	-4.26505700	-0.65966400

C	4.93826200	-5.53867900	-0.09133500

C	5.84725100	-3.34903800	-0.57270700

C	6.11436800	-5.88250800	0.56970700

H	4.11541600	-6.23983300	-0.17796900

C	7.02690800	-3.69683400	0.08523200

H	5.77975800	-2.36645400	-1.03014500

C	7.16157900	-4.96141000	0.66137500

H	6.21508400	-6.86833600	1.01540900

H	7.83876600	-2.97747400	0.14463500

H	8.08009300	-5.22928400	1.17685600

C	2.00366100	-2.21117700	-2.10768700

N	3.22453400	-2.64353700	-1.63214300

H	3.78225200	-1.94695500	-1.14589300

Ni	0.72609300	-0.85867500	0.10516300

C	2.48806600	-0.10025000	0.02115100

C	3.62284100	-0.26308500	0.73309600

H	2.52683500	0.65309400	-0.78033100

H	3.62629800	-0.98926700	1.54440000

C	4.90273500	0.45096400	0.55422400

C	5.98748700	0.11156800	1.38934300

C	5.11938600	1.45811700	-0.41115300

C	7.22294400	0.73952000	1.27352900

H	5.84593400	-0.66078100	2.14105100

C	6.34925500	2.08821400	-0.53218500

H	4.30923400	1.75215800	-1.07306500

C	7.41842900	1.73792000	0.30816800

H	8.04013000	0.46181900	1.93036800

H	6.50920800	2.86279700	-1.27565300

I	1.26679400	-2.79062200	1.88278100

C	8.70891300	2.44890900	0.13172500

O	8.91185100	3.32734400	-0.68685200

O	9.66462200	2.01491400	0.99120000

C	10.93516900	2.66644400	0.86942400

H	10.83861900	3.73991300	1.05503900

H	11.57510900	2.20397200	1.62205700

H	11.35108300	2.51885300	-0.13124100

H	-0.07347200	3.69223700	-0.83666800

H	1.56150300	2.34419500	0.03771900

C	1.01476300	2.15223600	2.08123500

C	2.04181900	2.98255200	2.55354300

C	0.38867100	1.27903700	2.97523200

C	2.42302800	2.95339600	3.89294800

H	2.55049500	3.65444600	1.86639000

C	0.77783500	1.24047500	4.31753000

H	-0.37711900	0.59456600	2.62729900

C	1.78980200	2.08010600	4.78128200

H	3.22245000	3.60261400	4.23956500

H	0.29309600	0.53972100	4.99092000

H	2.09486800	2.04507800	5.82366500

C	-0.42228100	4.64834600	1.03162300

C	0.28215300	5.78663800	0.61868000

C	-1.17549300	4.70584900	2.21076700

C	0.25604800	6.95501500	1.38124600

H	0.84841100	5.76065800	-0.30978600

C	-1.21478300	5.87803700	2.96460400

H	-1.74367100	3.84089500	2.53387600

C	-0.49417500	7.00308100	2.55684000

H	0.80985900	7.82862800	1.04880400

H	-1.80904100	5.91162500	3.87353800

H	-0.52437900	7.91395600	3.14843600

H	-3.22817100	-2.89196000	1.04214300

H	-1.05815100	-3.54036900	0.45729300

C	-4.02165500	-3.97348200	-0.62131800

C	-4.21985700	-5.15777600	0.10015100

C	-4.67619500	-3.80426400	-1.84634400

C	-5.04355700	-6.16466500	-0.40242300

H	-3.72899700	-5.29146700	1.06165200

C	-5.51052800	-4.80669400	-2.34278700

H	-4.53857800	-2.88584100	-2.40644800

C	-5.69308200	-5.99064300	-1.62614000

H	-5.18471700	-7.07936000	0.16669300

H	-6.01631900	-4.66207800	-3.29372400

H	-6.34102700	-6.77083500	-2.01620400

C	-1.20742900	-3.66360300	-1.67335300

C	-0.60162500	-4.92508200	-1.66288600

C	-1.49337800	-3.06404400	-2.90909300

C	-0.29123700	-5.57749200	-2.85742800

H	-0.34204700	-5.38647300	-0.71468100

C	-1.18150000	-3.71222000	-4.10360100

H	-1.95370900	-2.08216300	-2.94591300

C	-0.58183200	-4.97420600	-4.08037200

H	0.20420500	-6.54281900	-2.82654100

H	-1.40145100	-3.22942100	-5.05205800

H	-0.32969900	-5.47523200	-5.01096900

C	1.99619500	-1.02121200	-2.99813700

H	2.82319900	-0.32904900	-2.82001700

H	1.28366500	-2.99326100	-2.31071400

F	0.81191100	-0.30766300	-2.85908000

F	2.04073600	-1.39468500	-4.32114500

**INT-S**

C	0.84717100	-1.54605000	-0.13047300

C	2.15172500	-1.01362600	-0.69138500

C	2.26433300	0.49557200	-0.54072600

O	0.83616500	-2.87925300	0.06091600

C	-0.57311300	-3.26467000	0.14678100

C	-1.25780800	-1.91283200	0.55536300

N	-0.25215800	-0.91475800	0.08897500

N	1.34153700	1.37459100	-0.72032100

C	2.00910900	2.71447800	-0.72574500

C	3.33985700	2.37957400	0.01231100

O	3.50409800	0.95446000	-0.31277300

C	2.11293600	-1.24657500	-2.28418900

H	1.47114600	-0.45762900	-2.68091000

H	3.12708800	-1.04040200	-2.64247100

C	3.39088000	-1.74787400	-0.07139500

H	4.22609400	-1.52352200	-0.73026200

H	3.20129100	-2.81868900	-0.17898700

C	7.28241600	-0.01255600	1.04727700

C	6.03926200	-0.51812900	0.72118900

C	5.09981300	-0.90804600	1.70903500

C	5.47539800	-0.75839100	3.08493300

C	6.75693800	-0.23769100	3.38477700

C	7.64800900	0.12791600	2.39538700

C	3.79327100	-1.46228400	1.37458800

C	4.55175100	-1.14386800	4.14298300

C	3.28855800	-1.68311600	3.77725400

C	2.95900700	-1.83053800	2.39187300

C	2.37688900	-2.08854300	4.78146000

H	1.41442900	-2.49453200	4.48227300

C	2.69250000	-1.95909700	6.11945700

C	3.93814900	-1.41750000	6.48926900

C	4.84455900	-1.02108100	5.52249200

H	7.96916300	0.28348600	0.25934400

H	5.78155400	-0.59063200	-0.32742700

H	7.05701400	-0.11994800	4.41962800

H	8.62346700	0.52547500	2.66158000

H	2.00095300	-2.28233800	2.16112500

H	1.98428500	-2.27039200	6.88234800

H	4.19165200	-1.30884400	7.54030100

H	5.79400800	-0.60817700	5.84414200

C	4.57299500	-4.86296800	-2.98496500

C	3.79483700	-3.73771200	-2.78964100

C	2.39869200	-3.74272400	-3.02605600

C	1.79674500	-4.94799800	-3.51325400

C	2.61925000	-6.08468300	-3.69300600

C	3.97603100	-6.05300000	-3.43116200

C	1.58071700	-2.55833400	-2.83183800

C	0.37525000	-4.97490300	-3.83392500

C	-0.37383100	-3.77127200	-3.70748700

C	0.26651100	-2.59044000	-3.21197400

C	-1.74742900	-3.75537900	-4.04969700

H	-2.29719100	-2.82152700	-3.95644400

C	-2.38122400	-4.89930100	-4.49378200

C	-1.64923700	-6.09666000	-4.60786400

C	-0.30356800	-6.13085700	-4.28680800

H	5.64220600	-4.82282400	-2.79702900

H	4.27529100	-2.82506600	-2.46057100

H	2.18471000	-7.00825700	-4.05821200

H	4.57856300	-6.94398400	-3.58356100

H	-0.33315100	-1.68539600	-3.14200900

H	-3.43582200	-4.87733000	-4.75350800

H	-2.14223900	-7.00146200	-4.95244500

H	0.22779500	-7.07072400	-4.38623400

C	-1.86981900	4.81626100	-0.77410000

O	-0.73292200	5.26573300	-0.87795500

C	-2.98698200	5.62217900	-0.17950500

C	-2.64043500	6.65946200	0.69711100

C	-4.33835200	5.40441800	-0.48454700

C	-3.62951800	7.44808800	1.28004200

H	-1.58980100	6.82442000	0.91034500

C	-5.32755000	6.20178200	0.09215700

H	-4.62379600	4.63597200	-1.19784700

C	-4.97555300	7.22019800	0.97993300

H	-3.35202400	8.24138500	1.96885500

H	-6.37128200	6.03083900	-0.15723200

H	-5.74699600	7.83796500	1.43214600

C	-1.22725800	2.62303000	-1.65515000

N	-2.21357600	3.55160900	-1.21104800

H	-3.06749900	3.15271600	-0.84112700

Ni	-0.70554400	1.08066400	-0.48518400

C	-2.57072400	0.63532300	-0.62814900

C	-3.57226800	0.90782600	0.22486500

H	-2.76015400	-0.01631000	-1.48973500

H	-3.35657500	1.53330000	1.09117400

C	-4.95423800	0.39186600	0.16075200

C	-5.80218300	0.59308200	1.26712200

C	-5.47969500	-0.29757100	-0.95157300

C	-7.11037900	0.12026500	1.27409500

H	-5.41686700	1.12343800	2.13409300

C	-6.78333200	-0.77265200	-0.94980900

H	-4.86084500	-0.45282600	-1.83111600

C	-7.61450300	-0.57131900	0.16362800

H	-7.74537500	0.28389500	2.13776900

H	-7.18688400	-1.30093700	-1.80774500

I	-0.56131800	2.41797500	1.96844400

C	-9.00047300	-1.10310700	0.10570500

O	-9.46930100	-1.71365900	-0.83736700

O	-9.70710600	-0.83725100	1.23137900

C	-11.05499700	-1.32537900	1.23546300

H	-11.07191500	-2.41361400	1.12846200

H	-11.47104600	-1.02855800	2.19901100

H	-11.62844500	-0.88221200	0.41653300

H	-0.86703900	-3.49342500	-0.88266800

H	-2.15649100	-1.78811000	-0.05034000

C	-1.65984100	-1.76922400	2.01119100

C	-2.91742300	-2.25544300	2.39764400

C	-0.82595600	-1.20577900	2.98002500

C	-3.32437900	-2.20030800	3.72922700

H	-3.58462500	-2.67807300	1.65017100

C	-1.23572000	-1.14286700	4.31465700

H	0.12339900	-0.76867900	2.69413000

C	-2.48013300	-1.64507900	4.69416600

H	-4.30283600	-2.58057800	4.00999800

H	-0.58239300	-0.68121300	5.04926300

H	-2.79833900	-1.59046900	5.73190300

C	-0.77102700	-4.48971500	0.99505300

C	-1.78731200	-5.38430300	0.63469600

C	0.00361500	-4.76204400	2.12864400

C	-2.04426500	-6.51728800	1.40696000

H	-2.37707700	-5.19617400	-0.25990600

C	-0.24320400	-5.90278500	2.89231400

H	0.81315500	-4.09624200	2.40495900

C	-1.27141500	-6.77919800	2.53925600

H	-2.83792300	-7.19980600	1.11577200

H	0.37050400	-6.10616600	3.76553400

H	-1.46283000	-7.66539900	3.13810100

H	3.14168800	2.40930600	1.08942700

H	1.41524500	3.39527800	-0.11331100

C	4.58118200	3.16405900	-0.30431600

C	4.89797300	4.26206600	0.50509200

C	5.41385600	2.85083100	-1.38549900

C	6.01854600	5.04629200	0.22946000

H	4.26523500	4.50278100	1.35636600

C	6.54111900	3.62721200	-1.65295100

H	5.18099200	1.99793200	-2.01367400

C	6.84444500	4.72904200	-0.85004200

H	6.25020900	5.89692700	0.86445600

H	7.18177000	3.37288500	-2.49317000

H	7.72194000	5.33384400	-1.06234900

C	2.16894400	3.29476700	-2.12346400

C	2.05491500	4.68245400	-2.28804300

C	2.47830500	2.50910000	-3.24173400

C	2.25655100	5.26879600	-3.53752400

H	1.77274400	5.29978900	-1.44223700

C	2.67333400	3.09405500	-4.49359100

H	2.56075600	1.43201000	-3.14528000

C	2.56709600	4.47746400	-4.64437800

H	2.15496600	6.34502000	-3.64628400

H	2.90445300	2.46692200	-5.35073800

H	2.71503300	4.93362200	-5.61959600

C	-1.56824300	2.02240700	-3.00289200

H	-2.62336500	1.76644400	-3.12944200

H	-0.28654600	3.16331900	-1.74139700

F	-0.83576800	0.84180100	-3.17645100

F	-1.20853000	2.84567100	-4.02704700

**TS2-S**

C	0.82987600	-1.56241400	-0.14898900

C	2.15100700	-1.07796200	-0.72147400

C	2.36956900	0.41411000	-0.51953700

O	0.74172000	-2.90236400	-0.03629400

C	-0.68461100	-3.21131500	0.08241700

C	-1.28001400	-1.84062600	0.57088900

N	-0.22258300	-0.88477200	0.14512600

N	1.49326000	1.35128000	-0.56446800

C	2.22604000	2.65053000	-0.56254100

C	3.63196000	2.20496800	-0.04287900

O	3.65860900	0.78670400	-0.41758400

C	2.06214900	-1.23518800	-2.32185500

H	1.48293300	-0.37647300	-2.66794900

H	3.08274000	-1.09857800	-2.69445600

C	3.35624400	-1.90862200	-0.15808500

H	4.18865500	-1.72122100	-0.83195300

H	3.09604900	-2.96244800	-0.28358800

C	7.35669000	-0.40335100	0.91787600

C	6.08409100	-0.83971300	0.60561300

C	5.14255500	-1.19691700	1.60401300

C	5.54838100	-1.09042800	2.97542000

C	6.85967900	-0.64025900	3.26075100

C	7.75132300	-0.30370500	2.26155800

C	3.80375900	-1.67737500	1.28402900

C	4.62440100	-1.44754300	4.04310700

C	3.32918400	-1.91564100	3.69121900

C	2.96886800	-2.02064100	2.30980300

C	2.41515800	-2.29199500	4.70452400

H	1.42807500	-2.64274100	4.41582200

C	2.75985200	-2.20250900	6.03854400

C	4.03772000	-1.73154200	6.39496800

C	4.94658300	-1.36436600	5.41901900

H	8.04473400	-0.13064700	0.12265300

H	5.80511800	-0.88211000	-0.43908500

H	7.18283600	-0.55527300	4.29185700

H	8.75015900	0.03930900	2.51676100

H	1.98426400	-2.41764400	2.08869200

H	2.04975500	-2.49066500	6.80871500

H	4.31429000	-1.65456000	7.44296800

H	5.92122900	-1.00602600	5.73057100

C	4.18180800	-5.01752700	-3.21263800

C	3.50833800	-3.83791800	-2.95786000

C	2.11464300	-3.71060400	-3.17285900

C	1.40255200	-4.83632500	-3.69962000

C	2.11944000	-6.03165800	-3.94066700

C	3.47691500	-6.13003600	-3.69960000

C	1.40737600	-2.46854600	-2.91594700

C	-0.02018200	-4.72493000	-3.99585600

C	-0.65881300	-3.46712500	-3.80535900

C	0.08992600	-2.36904000	-3.27240200

C	-2.03057900	-3.31737500	-4.12134900

H	-2.49516100	-2.34437500	-3.97839800

C	-2.76885100	-4.38146300	-4.60101100

C	-2.14649200	-5.63192100	-4.77843100

C	-0.80440600	-5.79678000	-4.48394500

H	5.25250500	-5.07961900	-3.03970800

H	4.07076800	-2.98596400	-2.59742400

H	1.60082700	-6.89701600	-4.33742000

H	3.99672700	-7.06280700	-3.89925800

H	-0.42742100	-1.41925000	-3.15290600

H	-3.82136200	-4.25685500	-4.83925100

H	-2.72210600	-6.47471500	-5.15098600

H	-0.35896000	-6.77415400	-4.63196300

C	-1.99344600	4.95298100	-0.61463700

O	-0.82576500	5.30448200	-0.72888000

C	-3.04584100	5.86469000	-0.05441700

C	-2.62166700	6.89064000	0.80115700

C	-4.40755200	5.75257200	-0.37016600

C	-3.54710500	7.77291300	1.35370700

H	-1.56300300	6.97247100	1.02294300

C	-5.33197500	6.64417800	0.17534800

H	-4.74930400	4.99350600	-1.06875000

C	-4.90444300	7.65097000	1.04297600

H	-3.21096500	8.55674400	2.02698900

H	-6.38382800	6.55548400	-0.08252300

H	-5.62576400	8.34196600	1.47122100

C	-1.53207900	2.67753500	-1.40615300

N	-2.43631700	3.70032900	-0.99826600

H	-3.33534400	3.39539300	-0.64577600

Ni	-0.57638400	1.16058700	-0.20300200

C	-2.44197200	1.01243300	-0.59814300

C	-3.48374200	1.19871800	0.25509200

H	-2.58659700	0.35749100	-1.46101700

H	-3.32564200	1.83471100	1.12538800

C	-4.78899500	0.53804600	0.18629400

C	-5.65991800	0.63898700	1.29326100

C	-5.23572600	-0.21033100	-0.92699100

C	-6.90388700	0.02043100	1.29839700

H	-5.33831000	1.20763700	2.16188600

C	-6.47546300	-0.83102500	-0.92431900

H	-4.60669000	-0.29348200	-1.80919500

C	-7.32657400	-0.72666100	0.18826600

H	-7.55376300	0.11115800	2.16179100

H	-6.81553600	-1.40163500	-1.78275100

I	-0.42795500	2.49555700	2.18387200

C	-8.64022500	-1.41359900	0.13001200

O	-9.03528500	-2.07705800	-0.81184200

O	-9.37520100	-1.22783100	1.25455900

C	-10.65767600	-1.86763600	1.25788900

H	-10.54949800	-2.95105200	1.15477100

H	-11.10738800	-1.61756600	2.21964100

H	-11.27682400	-1.49631300	0.43645100

H	-1.02439100	-3.38003000	-0.94430200

H	-2.17624500	-1.62789800	-0.01628700

C	-1.65657100	-1.74123200	2.03763800

C	-2.92321300	-2.20113600	2.42697400

C	-0.79262000	-1.23214400	3.01045300

C	-3.30885600	-2.17591000	3.76568700

H	-3.61407100	-2.57829900	1.67677100

C	-1.18183800	-1.19836500	4.35235700

H	0.16537800	-0.81436300	2.72355300

C	-2.43485200	-1.67626100	4.73463300

H	-4.29434300	-2.53534400	4.04929600

H	-0.50550100	-0.77769600	5.09070700

H	-2.73692800	-1.64453100	5.77812400

C	-0.92072800	-4.45869900	0.88745100

C	-1.97831800	-5.29759100	0.51278800

C	-0.14012900	-4.80337700	1.99717500

C	-2.26858500	-6.44745700	1.24761300

H	-2.57508800	-5.05218000	-0.36305900

C	-0.42063300	-5.96076900	2.72294100

H	0.69908400	-4.18002200	2.28416500

C	-1.48887300	-6.78196900	2.35573600

H	-3.09413200	-7.08616000	0.94588700

H	0.19769000	-6.22053500	3.57776600

H	-1.70648300	-7.68142600	2.92516700

H	3.60382800	2.21469700	1.05254900

H	1.75659100	3.29900500	0.18009600

C	4.85901900	2.92656600	-0.52624600

C	5.31630100	4.02735400	0.20906500

C	5.54418900	2.55110800	-1.68749700

C	6.42903400	4.75245000	-0.21744400

H	4.80025900	4.31659500	1.12185000

C	6.66467100	3.26857700	-2.10700900

H	5.20473200	1.69294400	-2.25733800

C	7.10769500	4.37328800	-1.37696800

H	6.77033100	5.60583200	0.36201100

H	7.19052500	2.96500900	-3.00835900

H	7.97917400	4.93211400	-1.70712800

C	2.19327900	3.33361900	-1.91944200

C	1.98116100	4.71667700	-1.97599300

C	2.40514000	2.63593000	-3.11723700

C	1.98876200	5.38813500	-3.19932600

H	1.77406800	5.26537000	-1.06359300

C	2.40857700	3.30445600	-4.34091800

H	2.56376800	1.56236600	-3.10128100

C	2.20334700	4.68506400	-4.38469000

H	1.80907800	6.45921500	-3.22285800

H	2.56830300	2.74639100	-5.25986300

H	2.20086000	5.20621300	-5.33836200

C	-1.69485100	2.26711900	-2.85755300

H	-2.72115500	2.02606800	-3.15073300

H	-0.52516600	3.10095100	-1.30609800

F	-0.91449800	1.13712200	-3.08990700

F	-1.23782900	3.23705800	-3.69483600

**TS1-R**

C	-1.10624500	1.09412300	0.22361100

C	-2.42446100	0.61724700	-0.35424500

C	-2.44035000	-0.86519400	-0.65942200

O	-1.13479000	2.38484000	0.60224800

C	0.26652400	2.81359400	0.68210000

C	1.00375700	1.45049300	0.89898000

N	0.02333800	0.48037800	0.33198800

N	-1.47119400	-1.70817300	-0.69243600

C	-2.00474800	-2.97553200	-1.27922500

C	-3.53982700	-2.77321300	-1.09832800

O	-3.64579900	-1.31164400	-1.06906900

C	-2.60952600	1.26759700	-1.81557500

H	-1.85402000	0.78664200	-2.44392900

H	-3.57997900	0.91177700	-2.17021800

C	-3.62478800	1.03391800	0.57692500

H	-4.53616400	0.82415200	0.01907500

H	-3.56678000	2.11999800	0.67380200

C	-6.32725000	-2.29863200	1.64990900

C	-5.48547100	-1.24712000	1.33784300

C	-4.59283400	-0.69117500	2.28895900

C	-4.61198900	-1.22395100	3.61990700

C	-5.47600400	-2.30844500	3.90184000

C	-6.31409300	-2.84436800	2.94348900

C	-3.71005400	0.42384600	1.97201500

C	-3.78603300	-0.62494400	4.65866400

C	-3.00809100	0.51965000	4.33139300

C	-2.99486000	1.00304700	2.98382800

C	-2.25073100	1.16483700	5.33785000

H	-1.67583400	2.04795700	5.07305700

C	-2.23469300	0.68682400	6.63302100

C	-2.98074900	-0.46238700	6.95644800

C	-3.73932000	-1.09964800	5.99115300

H	-6.99666000	-2.69704600	0.89231300

H	-5.50972600	-0.85252500	0.33153600

H	-5.49674100	-2.72983300	4.90014300

H	-6.96730100	-3.67490800	3.19637200

H	-2.39077100	1.88018600	2.77076600

H	-1.64697300	1.18925700	7.39622500

H	-2.96422500	-0.85181300	7.97066700

H	-4.30233000	-1.98115400	6.27589000

C	-5.94642600	4.15525300	-1.55203900

C	-4.89336500	3.26542400	-1.64654400

C	-3.56887600	3.69556400	-1.90713000

C	-3.33865200	5.09588400	-2.10801700

C	-4.43655400	5.98084800	-1.99491300

C	-5.71329500	5.53014700	-1.71754000

C	-2.46104100	2.76268000	-2.01988400

C	-2.00369700	5.57326800	-2.44363500

C	-0.96377800	4.61794400	-2.61603000

C	-1.23596900	3.22887600	-2.40872000

C	0.33982100	5.04217800	-2.97197300

H	1.10934900	4.28403800	-3.09216000

C	0.62023800	6.38368000	-3.14653300

C	-0.40182400	7.33674700	-2.97028400

C	-1.68217900	6.93916800	-2.62751300

H	-6.95003900	3.78913200	-1.35439600

H	-5.09717500	2.20805600	-1.53128100

H	-4.28368500	7.04434300	-2.13998500

H	-6.53392500	6.23794500	-1.64083100

H	-0.41154600	2.53408900	-2.55484700

H	1.62212500	6.70476600	-3.41878700

H	-0.18600000	8.39333000	-3.10437200

H	-2.44276800	7.70103900	-2.49716300

C	2.97892200	1.27171200	-2.43862300

O	1.88798800	1.80150200	-2.67222400

C	4.24289500	2.04744200	-2.25738100

C	4.33193300	3.30542400	-2.87366400

C	5.32764000	1.58119500	-1.49577000

C	5.48917300	4.07021600	-2.75035100

H	3.49121400	3.65994900	-3.46064100

C	6.48247900	2.35296800	-1.36750400

H	5.27485000	0.63740600	-0.95917700

C	6.56797300	3.59575700	-1.99834200

H	5.55233800	5.03664200	-3.24296300

H	7.30979000	1.98087400	-0.76958600

H	7.46958300	4.19412700	-1.90095400

C	2.06805400	-0.99862500	-2.48043400

N	3.10437800	-0.10270200	-2.36190800

H	4.02639300	-0.47598000	-2.16978500

Ni	0.39817200	-1.55041600	0.13431400

C	2.28031600	-1.47426300	0.48300800

C	3.18507100	-2.47332300	0.57768300

H	2.69745000	-0.46310800	0.51228300

H	2.85166200	-3.50884100	0.52560800

C	4.64267000	-2.29372500	0.68223100

C	5.50716200	-3.32892100	0.26613100

C	5.23735100	-1.10945700	1.17492700

C	6.88967500	-3.17726100	0.28624800

H	5.07523400	-4.26291400	-0.08605000

C	6.61641500	-0.95377600	1.19556600

H	4.60289500	-0.32358400	1.57401100

C	7.46182800	-1.97812600	0.73761900

H	7.53234200	-3.98301900	-0.05175600

H	7.06837900	-0.04457600	1.58035100

I	-0.03814700	-3.68411700	1.69113400

C	8.92430900	-1.73573300	0.75620300

O	9.44952700	-0.68309700	1.07458600

O	9.63919100	-2.82095800	0.36786000

C	11.06116600	-2.64463400	0.36038300

H	11.42485200	-2.39565200	1.36123200

H	11.47315800	-3.59911200	0.03014000

H	11.34859200	-1.84413200	-0.32732600

H	0.51264700	3.15796400	-0.32626500

H	1.88086100	1.45171200	0.24893500

C	1.45824400	1.11678000	2.30971100

C	2.65436100	1.68913800	2.76999900

C	0.73882500	0.27456200	3.16233300

C	3.11581600	1.43354900	4.06010000

H	3.22412800	2.34501800	2.11479500

C	1.20477300	0.01224800	4.45397700

H	-0.17012600	-0.20650200	2.81912500

C	2.38975500	0.59133800	4.90687600

H	4.04353300	1.88539100	4.40101200

H	0.63824500	-0.65484300	5.09635300

H	2.75301800	0.38095200	5.90917500

C	0.42858400	3.96257200	1.63791400

C	0.97163600	5.15984900	1.15418300

C	0.04267200	3.88834700	2.98394000

C	1.13858600	6.25907400	1.99859700

H	1.25222200	5.23743400	0.10669900

C	0.19776200	4.98893400	3.82432400

H	-0.36406700	2.96361100	3.37683300

C	0.74940700	6.17630100	3.33539800

H	1.56149400	7.18014300	1.60718000

H	-0.10708500	4.91848000	4.86491500

H	0.87164700	7.03191200	3.99412000

H	-3.80944100	-3.10384200	-0.08942100

H	-1.67345200	-3.79677600	-0.64257800

C	-4.49279800	-3.36906500	-2.09647900

C	-5.04948200	-4.62450600	-1.81990400

C	-4.83068700	-2.72196300	-3.29135700

C	-5.91402900	-5.23346100	-2.73018200

H	-4.80571800	-5.12648100	-0.88622300

C	-5.70566200	-3.32506000	-4.19485300

H	-4.41468900	-1.74441900	-3.51026100

C	-6.24574300	-4.58334000	-3.92008900

H	-6.33524400	-6.20913400	-2.50387000

H	-5.96414000	-2.81106200	-5.11669900

H	-6.92534300	-5.05158800	-4.62689900

C	-1.52715900	-3.22411100	-2.69791700

C	-1.26984000	-4.54262300	-3.09251200

C	-1.36804400	-2.19936600	-3.63851600

C	-0.86157200	-4.83159700	-4.39432600

H	-1.37659400	-5.34699300	-2.36912500

C	-0.94985000	-2.48350300	-4.93937700

H	-1.55923200	-1.16864000	-3.35590100

C	-0.69548600	-3.80166300	-5.32124000

H	-0.65898700	-5.86044900	-4.67917800

H	-0.82045000	-1.67406500	-5.65305300

H	-0.36418300	-4.02299100	-6.33203400

C	2.43029600	-2.41356600	-2.75216000

H	3.38773100	-2.70130500	-2.30461500

H	1.11374900	-0.60178400	-2.79502000

F	2.52382800	-2.63812300	-4.11277900

F	1.46645100	-3.25908400	-2.28471200

**INT-R**

C	-0.73915200	1.03588900	0.26754600

C	-2.05116300	0.37184200	-0.10581600

C	-1.90513000	-1.10910200	-0.38104000

O	-0.87943700	2.34143600	0.58315800

C	0.44888000	2.93532700	0.47259000

C	1.38221300	1.67843300	0.60494000

N	0.45829000	0.56827400	0.25073300

N	-0.87921100	-1.77389800	-0.78264000

C	-1.34391000	-3.15026500	-1.12741600

C	-2.71294000	-3.20461000	-0.37782600

O	-3.06805800	-1.78359300	-0.31356700

C	-2.49539600	0.97029400	-1.54084600

H	-1.57533800	1.12683100	-2.11118900

H	-3.04288900	0.17155600	-2.04566900

C	-3.15139300	0.66899600	0.97885100

H	-4.11172500	0.43414300	0.53037800

H	-3.14517100	1.75379200	1.10954700

C	-5.84430900	-2.52861800	2.40716900

C	-4.98915900	-1.53201600	1.97797800

C	-3.93385500	-1.04501900	2.79075200

C	-3.79023500	-1.59669900	4.10684500

C	-4.67742700	-2.62286600	4.51094000

C	-5.68209100	-3.08753900	3.68512800

C	-3.03087600	0.00987000	2.34906500

C	-2.76974200	-1.08188500	5.00909300

C	-1.94552000	-0.01295300	4.56245700

C	-2.10789500	0.49304300	3.23405000

C	-0.98261100	0.54682400	5.43591800

H	-0.36374800	1.36466100	5.07703400

C	-0.81605500	0.05714100	6.71615100

C	-1.61482600	-1.01467100	7.15813100

C	-2.56896900	-1.56775600	6.32341700

H	-6.63803600	-2.87780300	1.75269900

H	-5.13422300	-1.12611700	0.98641400

H	-4.57797700	-3.05707400	5.49909300

H	-6.34685800	-3.87550400	4.02852900

H	-1.46622600	1.31651800	2.93572100

H	-0.07253400	0.49308300	7.37776900

H	-1.48427200	-1.41028700	8.16169100

H	-3.16824400	-2.38972400	6.69835300

C	-6.89025800	0.98687900	-1.56482300

C	-5.50817500	1.00485700	-1.55196500

C	-4.77383300	2.21821400	-1.58167000

C	-5.50331800	3.44931000	-1.64625000

C	-6.91745100	3.39477600	-1.65252100

C	-7.60339800	2.19596900	-1.60930700

C	-3.31940500	2.24177400	-1.58381200

C	-4.78678900	4.71573800	-1.72244100

C	-3.36479500	4.69121100	-1.77855200

C	-2.67558100	3.43948400	-1.71955800

C	-2.64280800	5.90390500	-1.88261700

H	-1.55708500	5.86050000	-1.92227000

C	-3.29709200	7.11888400	-1.92104200

C	-4.70327700	7.15094600	-1.85585000

C	-5.42842000	5.97657400	-1.75924400

H	-7.41989900	0.03845500	-1.54321700

H	-4.98120100	0.05752200	-1.52010400

H	-7.48822000	4.31525900	-1.70111200

H	-8.68976300	2.19152600	-1.61770900

H	-1.59277100	3.45316100	-1.80093900

H	-2.73342500	8.04461100	-1.99663300

H	-5.22499200	8.10381500	-1.88079100

H	-6.50977000	6.03968300	-1.70858500

C	1.71258500	1.75880500	-2.77602900

O	0.49756200	1.98502400	-2.84377200

C	2.71383200	2.87786700	-2.87229300

C	2.26658100	4.08450900	-3.43201200

C	4.03840700	2.79095000	-2.41248500

C	3.12716500	5.17425100	-3.54714200

H	1.23927400	4.14155700	-3.77590300

C	4.89657100	3.88592800	-2.52134000

H	4.41479700	1.88667200	-1.94056400

C	4.44506900	5.07762600	-3.09203200

H	2.77109500	6.09876400	-3.99332100

H	5.91636600	3.80646300	-2.15579700

H	5.11651500	5.92738800	-3.17901700

C	1.41940100	-0.66925400	-2.39426300

N	2.21614600	0.50399300	-2.61027700

H	3.21392700	0.39554700	-2.48590300

Ni	1.12536700	-1.33650800	-0.50799700

C	3.02771000	-1.14315000	-0.28407100

C	4.05209000	-1.89276300	-0.72488600

H	3.22879000	-0.26895200	0.33644600

H	3.84705000	-2.78396300	-1.31796100

C	5.48444900	-1.66210900	-0.46019400

C	6.43466500	-2.46777800	-1.11849200

C	5.96593400	-0.66259700	0.41243900

C	7.80044600	-2.28098800	-0.93575700

H	6.08694200	-3.25393700	-1.78480500

C	7.32758500	-0.47107700	0.59552500

H	5.26616200	-0.04399300	0.96655300

C	8.26325200	-1.27274300	-0.07861700

H	8.51261800	-2.91304800	-1.45477400

H	7.69512400	0.29629200	1.26940500

I	1.13349300	-3.01507200	1.62220200

C	9.70551200	-1.01348900	0.15533000

O	10.14615600	-0.13549300	0.87485000

O	10.50640600	-1.86844600	-0.52928100

C	11.91268600	-1.66922200	-0.34039300

H	12.18221400	-1.79025200	0.71256600

H	12.40121600	-2.43030500	-0.95032200

H	12.20899300	-0.66800600	-0.66608000

H	0.52220200	3.28959500	-0.56012400

H	2.13374500	1.74671800	-0.18365800

C	2.12072400	1.49462200	1.91953900

C	3.30662800	2.22136700	2.11624200

C	1.68033000	0.63261700	2.92673900

C	4.02757400	2.10063800	3.30279700

H	3.66508300	2.88859300	1.33530400

C	2.40971700	0.50301600	4.11278300

H	0.79590900	0.02489100	2.77526100

C	3.57868400	1.23763300	4.30677100

H	4.94168500	2.67208500	3.44010100

H	2.06036300	-0.18629200	4.87554600

H	4.14566100	1.13092200	5.22760100

C	0.62256100	4.09812700	1.40950400

C	1.39765000	5.18431600	0.98188800

C	0.05264600	4.13104600	2.68749500

C	1.62047200	6.27372500	1.82481000

H	1.82789400	5.17749900	-0.01757300

C	0.26322800	5.22644400	3.52466500

H	-0.57219700	3.31052900	3.02229300

C	1.05239000	6.29734000	3.09943200

H	2.22626700	7.10743600	1.48033700

H	-0.19153300	5.24308600	4.51130400

H	1.21627800	7.14830500	3.75485700

H	-2.51726600	-3.50212000	0.65766600

H	-0.65930200	-3.86067100	-0.66048800

C	-3.84073300	-4.02525000	-0.93753400

C	-4.08012200	-5.29206300	-0.39050800

C	-4.64751900	-3.57504600	-1.99068200

C	-5.09510600	-6.10474200	-0.89733000

H	-3.47169800	-5.64149100	0.44045400

C	-5.67072600	-4.38149300	-2.48800600

H	-4.47476900	-2.59485600	-2.42204100

C	-5.89476100	-5.64969100	-1.94672500

H	-5.26686900	-7.08617900	-0.46395500

H	-6.29167600	-4.01977800	-3.30313900

H	-6.69111500	-6.27692800	-2.33813600

C	-1.39981200	-3.41368200	-2.62227400

C	-1.17242800	-4.71976800	-3.07602300

C	-1.71671000	-2.42414300	-3.55954100

C	-1.26850600	-5.03189700	-4.43120900

H	-0.91370900	-5.49682000	-2.36083400

C	-1.80466200	-2.73140600	-4.91868600

H	-1.87506400	-1.39994300	-3.23701600

C	-1.58537200	-4.03678100	-5.35842800

H	-1.08533900	-6.05009700	-4.76370000

H	-2.03899600	-1.94639500	-5.63264000

H	-1.65178000	-4.27592400	-6.41630200

C	1.96408100	-1.82415200	-3.20860700

H	3.05286400	-1.81575900	-3.30433200

H	0.39901100	-0.44426200	-2.69800100

F	1.43613700	-1.84261700	-4.47048700

F	1.61903900	-3.02141400	-2.60839200

**TS2-R**

C	-0.77781300	1.00269200	0.41793500

C	-2.09221500	0.39218000	-0.03674100

C	-1.97005000	-1.06029300	-0.44342000

O	-0.92340800	2.25842600	0.89281300

C	0.40018900	2.86622200	0.88902400

C	1.34338400	1.60923500	0.82603400

N	0.42252200	0.54598200	0.34611900

N	-0.94504500	-1.69921600	-0.87896200

C	-1.41424400	-3.03100900	-1.35006100

C	-2.82297100	-3.12515700	-0.67467500

O	-3.15015900	-1.71223300	-0.46679800

C	-2.49927600	1.12129600	-1.41870800

H	-1.57019000	1.25643200	-1.97965800

H	-3.09616700	0.39565200	-1.97560600

C	-3.20208000	0.59637800	1.05696500

H	-4.16032500	0.45074500	0.56950700

H	-3.16693900	1.65444600	1.32759100

C	-6.12607700	-2.54142700	2.09518800

C	-5.19327000	-1.57111900	1.78435800

C	-4.10301800	-1.27381400	2.64150800

C	-3.99817400	-2.00222000	3.87258300

C	-4.96728000	-2.99412100	4.15642300

C	-6.00974200	-3.26600200	3.29244300

C	-3.11999300	-0.24541900	2.32665900

C	-2.92617100	-1.70282900	4.81161000

C	-2.00926400	-0.66594300	4.48715400

C	-2.14402600	0.02869000	3.24362000

C	-0.98205300	-0.32213500	5.39822200

H	-0.29071900	0.47272700	5.13182800

C	-0.84504000	-0.99143200	6.59795000

C	-1.73966100	-2.02998800	6.91902500

C	-2.75562600	-2.37434900	6.04599100

H	-6.94503800	-2.74255400	1.41019600

H	-5.30430700	-1.03692800	0.85053900

H	-4.90031500	-3.55811300	5.07963400

H	-6.73693100	-4.03363900	3.54277000

H	-1.43084500	0.82216400	3.04304700

H	-0.05096000	-0.72187900	7.28884300

H	-1.63369200	-2.56571400	7.85842100

H	-3.42676600	-3.17810100	6.32692900

C	-6.88445900	1.44450800	-1.41986500

C	-5.50456000	1.36495200	-1.41929300

C	-4.68710500	2.52247800	-1.35366600

C	-5.32856000	3.80270100	-1.31284600

C	-6.74304300	3.84745900	-1.30699700

C	-7.51111300	2.69975600	-1.35453000

C	-3.23480300	2.44523000	-1.36710000

C	-4.52541300	5.01838200	-1.30191800

C	-3.10935100	4.89966700	-1.38299500

C	-2.50958500	3.60212900	-1.42408900

C	-2.30543400	6.06405500	-1.41135600

H	-1.22594500	5.94889000	-1.47514400

C	-2.87298500	7.32093600	-1.34901200

C	-4.27245900	7.44523800	-1.25579200

C	-5.07702700	6.31986300	-1.23365100

H	-7.47944900	0.53705800	-1.47417500

H	-5.04514700	0.38425300	-1.47712500

H	-7.24804800	4.80628800	-1.27469700

H	-8.59513500	2.77151400	-1.35120500

H	-1.42883700	3.54609000	-1.51946300

H	-2.24673400	8.20860200	-1.36695000

H	-4.72611900	8.43107800	-1.20064200

H	-6.15054800	6.45360900	-1.15946600

C	1.84903800	2.01894300	-2.64092600

O	0.62394600	2.13237600	-2.53384200

C	2.73355100	3.21753000	-2.82395300

C	2.14829400	4.37061000	-3.36823400

C	4.08756100	3.24647700	-2.45332500

C	2.90762800	5.52194700	-3.56317500

H	1.09796000	4.33948500	-3.63848100

C	4.84353600	4.40426200	-2.64042800

H	4.55938700	2.38548500	-1.98599600

C	4.25749300	5.54091800	-3.20083100

H	2.44783100	6.40502700	-3.99805400

H	5.88796700	4.41799800	-2.34237200

H	4.84934800	6.43978700	-3.34978100

C	1.81554900	-0.43686300	-2.33816100

N	2.47967900	0.80518800	-2.59759400

H	3.48723700	0.78178100	-2.68451500

Ni	1.08605900	-1.30225600	-0.47287400

C	2.96776400	-0.93660900	-0.71549100

C	4.00034400	-1.77883600	-0.98377400

H	3.20005300	0.03475100	-0.28235600

H	3.79248700	-2.72966700	-1.47105400

C	5.40349700	-1.56313100	-0.63076100

C	6.37067600	-2.48134800	-1.09511800

C	5.85978100	-0.47538100	0.14958000

C	7.72295100	-2.31431300	-0.82499000

H	6.04330800	-3.33725600	-1.68050300

C	7.20888400	-0.30551100	0.41876600

H	5.14610100	0.22380800	0.57535300

C	8.16085000	-1.21629100	-0.06924700

H	8.44624000	-3.03144200	-1.19730800

H	7.55436600	0.52851300	1.02137600

I	1.20517600	-3.34750700	1.28753400

C	9.58863700	-0.97429300	0.24892700

O	10.00787600	-0.02489000	0.88663200

O	10.40485400	-1.93483800	-0.25392100

C	11.79871000	-1.75769800	0.02590400

H	11.97873900	-1.75155300	1.10461500

H	12.30164800	-2.60567100	-0.44094200

H	12.16190500	-0.81628300	-0.39622500

H	0.48065400	3.38447100	-0.07178300

H	2.06939600	1.80000200	0.03248900

C	2.12235900	1.24751500	2.07852000

C	3.28643000	1.97752200	2.36882600

C	1.73955200	0.21474500	2.93794100

C	4.04165500	1.69231300	3.50480600

H	3.59943100	2.78040200	1.70448800

C	2.50411300	-0.07953400	4.07120600

H	0.87453300	-0.39608500	2.70719800

C	3.65035300	0.65913200	4.36105500

H	4.93833900	2.26869400	3.71623500

H	2.20187600	-0.90176200	4.71288500

H	4.24526600	0.42441500	5.23950400

C	0.55726800	3.86371200	2.00367700

C	1.31064100	5.01961600	1.76185300

C	-0.00811000	3.67399200	3.27003400

C	1.51769700	5.95870200	2.77282600

H	1.73677800	5.18634800	0.77455100

C	0.18672400	4.61903200	4.27696500

H	-0.61745600	2.79854900	3.46509900

C	0.95482700	5.75995100	4.03433400

H	2.10658100	6.84914900	2.57038200

H	-0.26375700	4.46316200	5.25329200

H	1.10637000	6.49322300	4.82165600

H	-2.68735300	-3.54302000	0.32858300

H	-0.76286500	-3.78769700	-0.90776400

C	-3.93998200	-3.84476100	-1.37754600

C	-4.24152300	-5.15619900	-0.99014200

C	-4.67513600	-3.25571000	-2.41445400

C	-5.24758000	-5.87504500	-1.63736600

H	-3.68856000	-5.61504400	-0.17373000

C	-5.68940800	-3.96898400	-3.05234100

H	-4.45387500	-2.23926200	-2.72244600

C	-5.97585500	-5.28158200	-2.66935900

H	-5.46819200	-6.89258600	-1.32662600

H	-6.25453500	-3.49968100	-3.85318500

H	-6.76504500	-5.83608400	-3.16993300

C	-1.38354200	-3.16763300	-2.86222900

C	-1.17440900	-4.43856400	-3.41419400

C	-1.58951200	-2.08617700	-3.72634500

C	-1.18307400	-4.62704700	-4.79551300

H	-1.00017700	-5.28551100	-2.75500800

C	-1.58913700	-2.27014600	-5.11040600

H	-1.72777600	-1.08783300	-3.32273800

C	-1.39118000	-3.54154100	-5.64925500

H	-1.01699700	-5.61993700	-5.20483300

H	-1.73785400	-1.41627300	-5.76598000

H	-1.38952700	-3.68475700	-6.72645100

C	2.17918100	-1.48612700	-3.36869600

H	3.25435600	-1.56645400	-3.55355300

H	0.74552800	-0.23297400	-2.41504100

F	1.56914900	-1.21131900	-4.56186500

F	1.72975700	-2.70985200	-2.94265800

**L9Ni(II)I**

C	1.12559100	0.19665600	-0.60972300

C	-0.22461900	0.79413700	-0.25604700

C	-1.29866000	-0.25676200	-0.06106500

O	2.13715800	1.10155300	-0.67354900

C	3.20263900	0.46630800	-1.44583700

C	2.83960800	-1.06199000	-1.33945800

N	1.40697400	-1.00441700	-0.96452700

N	-1.27524300	-1.51633000	-0.31298700

C	-2.63624400	-2.05882800	-0.06986700

C	-3.30432500	-0.89519800	0.74724700

O	-2.47672100	0.25131400	0.37761900

C	-0.68655400	1.64745000	-1.54719500

H	-0.19100300	1.19923700	-2.41385600

H	-1.75190100	1.46076100	-1.68949800

C	-0.11606300	1.72062600	1.01215400

H	-0.99598900	2.35853500	1.02158300

H	0.73202700	2.38418400	0.82764300

C	-3.27861200	1.45426300	4.08239800

C	-2.27439200	1.53205500	3.13599300

C	-0.99446600	0.95948000	3.35201400

C	-0.74758800	0.31468800	4.60938500

C	-1.80107100	0.24685600	5.55230100

C	-3.04289400	0.79726300	5.30127500

C	0.07119500	1.04050300	2.36243400

C	0.57024300	-0.23020600	4.90552000

C	1.59953000	-0.08542700	3.93459600

C	1.30543600	0.54980100	2.68640200

C	2.90258400	-0.56429400	4.21171300

H	3.67489000	-0.44625500	3.45648600

C	3.18947600	-1.18631500	5.41071000

C	2.17166900	-1.34675400	6.37029200

C	0.89417400	-0.87827900	6.12170500

H	-4.24660500	1.90426100	3.88036300

H	-2.48544400	2.04138200	2.20550500

H	-1.63510600	-0.24132600	6.50576700

H	-3.82786400	0.73012300	6.04959400

H	2.12018900	0.65107100	1.97527200

H	4.19267400	-1.55120100	5.61277300

H	2.38926200	-1.83961800	7.31384700

H	0.13652600	-1.01636400	6.88482700

C	-3.64832200	4.56857300	-0.16648600

C	-2.69005600	3.66481200	-0.58676400

C	-1.43763200	4.08976600	-1.09991800

C	-1.17569800	5.49589600	-1.17746500

C	-2.17931700	6.39181600	-0.73864900

C	-3.39031300	5.94695700	-0.24314100

C	-0.43244300	3.14344800	-1.55789000

C	0.09667500	5.97146000	-1.70480800

C	1.04859200	5.01397200	-2.15502500

C	0.73959100	3.61989600	-2.07343200

C	2.28977300	5.44564700	-2.68006100

H	3.00290500	4.69628100	-3.01515700

C	2.59720400	6.78903500	-2.76058700

C	1.66143200	7.74190400	-2.31456400

C	0.44154900	7.34069300	-1.79963700

H	-4.59819000	4.20812600	0.21862600

H	-2.91086700	2.60674300	-0.50755100

H	-2.00354900	7.46027900	-0.79145200

H	-4.13853500	6.66364700	0.08371600

H	1.48668000	2.92035200	-2.43695000

H	3.55376800	7.11045700	-3.16284700

H	1.89721200	8.80092300	-2.37361000

H	-0.25329300	8.10240500	-1.46397800

Ni	0.19825200	-2.58132300	-1.10843700

I	0.63803100	-4.85425500	-2.11855700

H	3.03571900	0.77469400	-2.48572600

H	2.89825800	-1.50339200	-2.33805700

C	3.67630200	-1.91919900	-0.40646800

C	4.87566000	-2.45827600	-0.89151300

C	3.29430800	-2.19628300	0.90969600

C	5.68591900	-3.24371800	-0.07402500

H	5.17258300	-2.27105000	-1.92067100

C	4.10122300	-2.99140700	1.72781000

H	2.35100800	-1.82007100	1.29080400

C	5.29990300	-3.51257800	1.24113600

H	6.60982100	-3.65767300	-0.46857100

H	3.78147900	-3.20705600	2.74329800

H	5.92317500	-4.13625400	1.87631000

C	4.56205500	0.94079900	-1.01049200

C	5.56749600	1.06919100	-1.97733300

C	4.86085300	1.24117300	0.32350000

C	6.85472700	1.46897800	-1.61699500

H	5.34230800	0.85495700	-3.02015300

C	6.14365800	1.65424400	0.68246700

H	4.08624200	1.17008300	1.07888500

C	7.14559800	1.76372400	-0.28417600

H	7.62428600	1.56045200	-2.37850200

H	6.36027500	1.89158700	1.72040800

H	8.14455900	2.08367400	-0.00108100

H	-3.10631600	-1.06693400	1.81079400

H	-2.54231800	-2.93434300	0.57734800

C	-4.76492400	-0.58999400	0.55922700

C	-5.67321200	-1.01770800	1.53532500

C	-5.24639000	0.09984300	-0.56155200

C	-7.04057800	-0.77851500	1.38911900

H	-5.30719000	-1.53669000	2.41832800

C	-6.61071900	0.35116100	-0.70139400

H	-4.55477700	0.43810600	-1.32568500

C	-7.51213700	-0.09080400	0.27030300

H	-7.73292100	-1.11976600	2.15383300

H	-6.97097400	0.88886300	-1.57416000

H	-8.57516300	0.10345500	0.15671500

C	-3.32981100	-2.48524800	-1.35170600

C	-4.25200000	-3.53777200	-1.30116400

C	-3.09900900	-1.84807700	-2.57666900

C	-4.93941300	-3.93856200	-2.44614800

H	-4.42738600	-4.05356500	-0.35994200

C	-3.78235700	-2.25068700	-3.72505700

H	-2.36229000	-1.05337600	-2.64381100

C	-4.70658200	-3.29430400	-3.66267000

H	-5.64597600	-4.76202000	-2.39015400

H	-3.58171200	-1.75558500	-4.67132900

H	-5.23236800	-3.61259000	-4.55864200

**(S)-3a**

C	3.45041400	-0.09123000	0.71303900

O	3.68329500	-0.53902600	1.82930000

C	4.14871500	1.12842200	0.18315200

C	4.71245000	2.00278800	1.12212500

C	4.28162600	1.41225000	-1.18354100

C	5.37231300	3.15543000	0.70356200

H	4.62218300	1.75580500	2.17473100

C	4.95045500	2.56264000	-1.60214000

H	3.90255400	0.72017700	-1.93116300

C	5.49013300	3.43947400	-0.65955400

H	5.79894400	3.83144400	1.43942900

H	5.05794200	2.76820100	-2.66358700

H	6.00912200	4.33644000	-0.98653900

C	1.77416400	-1.84753500	0.24288400

N	2.51406000	-0.65920100	-0.12335300

H	2.23964400	-0.16722400	-0.96177000

C	0.27476600	-1.68096200	0.17605900

C	-0.36948300	-0.50577000	0.13114500

H	-0.27725400	-2.61699200	0.19281500

H	0.22265200	0.40844800	0.15029600

C	-1.82319400	-0.29580300	0.08316400

C	-2.31548800	1.02119500	0.12735900

C	-2.75690200	-1.34691600	-0.00954100

C	-3.68024800	1.28712700	0.08724200

H	-1.61163800	1.84694300	0.19896600

C	-4.11881100	-1.08759700	-0.05089200

H	-2.41385800	-2.37614400	-0.05225200

C	-4.59589400	0.23140600	-0.00266300

H	-4.04014300	2.30909700	0.12506700

H	-4.83837100	-1.89655300	-0.12249600

C	-6.06815000	0.44434200	-0.05199400

O	-6.89060300	-0.44740800	-0.13488800

O	-6.40381600	1.75471000	0.00572500

C	-7.81261000	2.02688500	-0.03573700

H	-8.32035000	1.55436000	0.80935900

H	-7.90216800	3.11221400	0.02052900

H	-8.24937800	1.65210700	-0.96531100

C	2.22933200	-3.03595900	-0.61777600

H	2.04137500	-2.89414300	-1.68918400

H	2.08265700	-2.08949000	1.26864600

F	1.56185300	-4.16272700	-0.21358200

F	3.56094200	-3.24923000	-0.43980900

**(R)-3a**

C	-3.79404900	-0.19521500	0.68418900

O	-4.06114500	-0.89982400	1.64985900

C	-4.75059300	0.84035100	0.16586500

C	-6.10529000	0.67621300	0.48468300

C	-4.35393200	1.95533700	-0.58625000

C	-7.05058900	1.59526500	0.03615100

H	-6.39262900	-0.17972600	1.08620600

C	-5.30040400	2.88026000	-1.02718500

H	-3.30309300	2.13280000	-0.80063700

C	-6.65057300	2.69796600	-0.72302500

H	-8.09977200	1.45435300	0.28081300

H	-4.98188700	3.74660500	-1.60034200

H	-7.38749500	3.41746100	-1.06957900

C	-1.60859100	-1.31242500	0.35977500

N	-2.59119900	-0.29396300	0.01737500

H	-2.48044400	0.19144800	-0.86200800

C	-0.23136300	-0.69339600	0.42782700

C	0.91795300	-1.21261400	-0.03240200

H	-0.23659200	0.27985100	0.91477000

H	0.90646700	-2.19855500	-0.48986700

C	2.24808100	-0.58913900	0.03201400

C	3.34283700	-1.29029300	-0.50565400

C	2.49116300	0.67681900	0.60074700

C	4.62793300	-0.75842700	-0.48233500

H	3.17746900	-2.27011300	-0.94707200

C	3.77025700	1.21255500	0.62627800

H	1.67298500	1.24754900	1.02930200

C	4.85268600	0.50197500	0.08492200

H	5.45840000	-1.31527000	-0.90141900

H	3.95776900	2.18729600	1.06453300

C	6.20033400	1.13105300	0.14199200

O	6.43018200	2.22456200	0.62170500

O	7.16038500	0.34842300	-0.40444300

C	8.48571000	0.89916600	-0.37896700

H	8.80534800	1.08402300	0.65007500

H	9.12248500	0.14974000	-0.85012100

H	8.52190200	1.83981900	-0.93503400

C	-1.72625100	-2.49987900	-0.60266900

H	-1.40875200	-2.25896300	-1.62482300

H	-1.91283100	-1.68524400	1.34629900

F	-3.01514600	-2.93391800	-0.64721000

F	-0.95243400	-3.53764400	-0.15116900

NMR Spectra of Products (^1^H NMR, ^19^F NMR, ^13^C NMR)

^1^H NMR (400 MHz, CDCl_3_) Spectrum of Compound **1a**

^13^C NMR (101 MHz, CDCl_3_) Spectrum of Compound **1a**

^19^F NMR (376 MHz, CDCl_3_) Spectrum of Compound **1a**

^1^H NMR (400 MHz, CDCl_3_) Spectrum of Compound **1b**

^13^C NMR (101 MHz, CDCl_3_) Spectrum of Compound **1b**

^19^F NMR (376 MHz, CDCl_3_) Spectrum of Compound **1b**

^1^ H NMR (400 MHz, CDCl_3_) Spectrum of Compound **1c**

^13^ C NMR (101 MHz, CDCl_3_) Spectrum of Compound **1c**

^19^ F NMR (376 MHz, CDCl_3_) Spectrum of Compound **1c**

^1^ H NMR (400 MHz, CDCl_3_) Spectrum of Compound **1d**

^13^ C NMR (101 MHz, CDCl_3_) Spectrum of Compound **1d**

^19^ F NMR (376 MHz, CDCl_3_) Spectrum of Compound **1d**

^1^ H NMR (400 MHz, CDCl_3_) Spectrum of Compound **1e**

^13^ C NMR (101 MHz, CDCl_3_) Spectrum of Compound **1e**

^19^ F NMR (376 MHz, CDCl_3_) Spectrum of Compound **1e**

^1^ H NMR (400 MHz, CDCl_3_) Spectrum of Compound **1f**

^13^ C NMR (101 MHz, CDCl_3_) Spectrum of Compound **1f**

^19^ F NMR (376 MHz, CDCl_3_) Spectrum of Compound **1f**

^1^ H NMR (400 MHz, CDCl_3_) Spectrum of Compound **1g**

^13^ C NMR (101 MHz, CDCl_3_) Spectrum of Compound **1g**

^19^ F NMR (376 MHz, CDCl_3_) Spectrum of Compound **1g**

^1^ H NMR (400 MHz, CDCl_3_) Spectrum of Compound **1h**

^13^ C NMR (101 MHz, CDCl_3_) Spectrum of Compound **1h**

^19^ F NMR (376 MHz, CDCl_3_) Spectrum of Compound **1h**

^1^ H NMR (400 MHz, CDCl_3_) Spectrum of Compound **1i**

^13^ C NMR (101 MHz, CDCl_3_) Spectrum of Compound **1i**

^19^ F NMR (376 MHz, CDCl_3_) Spectrum of Compound **1i**

^1^ H NMR (400 MHz, CDCl_3_) Spectrum of Compound **1j**

^13^ C NMR (101 MHz, CDCl_3_) Spectrum of Compound **1j**

^19^ F NMR (376 MHz, CDCl_3_) Spectrum of Compound **1j**

^1^ H NMR (400 MHz, CDCl_3_) Spectrum of Compound **1k**

^13^ C NMR (101 MHz, CDCl_3_) Spectrum of Compound **1k**

^19^ F NMR (376 MHz, CDCl_3_) Spectrum of Compound **1k**

^1^ H NMR (400 MHz, CDCl_3_) Spectrum of Compound **1l**

^13^ C NMR (101 MHz, CDCl_3_) Spectrum of Compound **1l**

^19^ F NMR (376 MHz, CDCl_3_) Spectrum of Compound **1l**

^1^ H NMR (400 MHz, CDCl_3_) Spectrum of Compound **1m**

^13^ C NMR (101 MHz, CDCl_3_) Spectrum of Compound **1m**

^19^ F NMR (376 MHz, CDCl_3_) Spectrum of Compound **1m**

^1^ H NMR (400 MHz, CDCl_3_) Spectrum of Compound **1n**

^13^ C NMR (101 MHz, CDCl_3_) Spectrum of Compound **1n**

^19^ F NMR (376 MHz, CDCl_3_) Spectrum of Compound **1n**

^1^ H NMR (400 MHz, CDCl_3_) Spectrum of Compound **4a**

^13^ C NMR (101 MHz, CDCl_3_) Spectrum of Compound **4a**

^19^ F NMR (376 MHz, CDCl_3_) Spectrum of Compound **4a**

^1^ H NMR (500 MHz, CDCl_3_) Spectrum of Compound **4b**

^13^ C NMR (126 MHz, CDCl_3_) Spectrum of Compound **4b**

^19^ F NMR (471 MHz, CDCl_3_) Spectrum of Compound **4b**

^1^ H NMR (500 MHz, CDCl_3_) Spectrum of Compound **4c**

^13^ C NMR (126 MHz, CDCl_3_) Spectrum of Compound **4c**

^19^ F NMR (471 MHz, CDCl_3_) Spectrum of Compound **4c**

^1^ H NMR (500 MHz, CDCl_3_) Spectrum of Compound **4d**

^13^ C NMR (126 MHz, CDCl_3_) Spectrum of Compound **4d**

^19^ F NMR (471 MHz, CDCl_3_) Spectrum of Compound **4d**

^1^ H NMR (500 MHz, CDCl_3_) Spectrum of Compound **4e**

^13^ C NMR (126 MHz, CDCl_3_) Spectrum of Compound **4e**

^19^ F NMR (471 MHz, CDCl_3_) Spectrum of Compound **4e**

^1^ H NMR (500 MHz, CDCl_3_) Spectrum of Compound **4f**

^13^ C NMR (126 MHz, CDCl_3_) Spectrum of Compound **4f**

^19^ F NMR (471 MHz, CDCl_3_) Spectrum of Compound **4f**

^1^ H NMR (500 MHz, CDCl_3_) Spectrum of Compound **4g**

^13^ C NMR (126 MHz, CDCl_3_) Spectrum of Compound **4g**

^19^ F NMR (471 MHz, CDCl_3_) Spectrum of Compound **4g**

^1^ H NMR (500 MHz, CDCl_3_) Spectrum of Compound **4h**

^13^ C NMR (126 MHz, CDCl_3_) Spectrum of Compound **4h**

^19^ F NMR (471 MHz, CDCl_3_) Spectrum of Compound **4h**

^1^ H NMR (400 MHz, CDCl_3_) Spectrum of Compound **4i**

^13^ C NMR (101 MHz, CDCl_3_) Spectrum of Compound **4i**

^19^ F NMR (376 MHz, CDCl_3_) Spectrum of Compound **4i**

^1^ H NMR (500 MHz, CDCl_3_) Spectrum of Compound **4j**

^13^ C NMR (126 MHz, CDCl_3_) Spectrum of Compound **4j**

^19^ F NMR (471 MHz, CDCl_3_) Spectrum of Compound **4j**

^1^ H NMR (500 MHz, CDCl_3_) Spectrum of Compound **3a**

^13^ C NMR (126 MHz, CDCl_3_) Spectrum of Compound **3a**

^19^ F NMR (471 MHz, CDCl_3_) Spectrum of Compound **3a**

^1^ H NMR (600 MHz, CDCl_3_) Spectrum of Compound **3b**

^13^ C NMR (151 MHz, CDCl_3_) Spectrum of Compound **3b**

^19^ F NMR (565 MHz, CDCl_3_) Spectrum of Compound **3b**

^1^ H NMR (500 MHz, CDCl_3_) Spectrum of Compound **3c**

^13^ C NMR (126 MHz, CDCl_3_) Spectrum of Compound **3c**

^19^ F NMR (471 MHz, CDCl_3_) Spectrum of Compound **3c**

^1^ H NMR (500 MHz, CDCl_3_) Spectrum of Compound **3d**

^13^ C NMR (126 MHz, CDCl_3_) Spectrum of Compound **3d**

^19^ F NMR (471 MHz, CDCl_3_) Spectrum of Compound **3d**

^1^ H NMR (500 MHz, CDCl_3_) Spectrum of Compound **3e**

^13^ C NMR (126 MHz, CDCl_3_) Spectrum of Compound **3e**

^19^ F NMR (471 MHz, CDCl_3_) Spectrum of Compound **3e**

^1^ H NMR (500 MHz, CDCl_3_) Spectrum of Compound **3f**

^13^ C NMR (126 MHz, CDCl_3_) Spectrum of Compound **3f**

^19^ F NMR (471 MHz, CDCl_3_) Spectrum of Compound **3f**

^1^ H NMR (500 MHz, CDCl_3_) Spectrum of Compound **3g**

^13^ C NMR (126 MHz, CDCl_3_) Spectrum of Compound **3g**

^19^ F NMR (471 MHz, CDCl_3_) Spectrum of Compound **3g**

^1^ H NMR (500 MHz, CDCl_3_) Spectrum of Compound **3h**

^13^ C NMR (126 MHz, CDCl_3_) Spectrum of Compound **3h**

^19^ F NMR (471 MHz, CDCl_3_) Spectrum of Compound **3h**

^1^ H NMR (600 MHz, CDCl_3_) Spectrum of Compound **3i**

^13^ C NMR (151 MHz, CDCl_3_) Spectrum of Compound **3i**

^19^ F NMR (565 MHz, CDCl_3_) Spectrum of Compound **3i**

^1^ H NMR (500 MHz, CDCl_3_) Spectrum of Compound **3j**

^13^ C NMR (126 MHz, CDCl_3_) Spectrum of Compound **3j**

^19^ F NMR (471 MHz, CDCl_3_) Spectrum of Compound **3j**

^1^ H NMR (500 MHz, CDCl_3_) Spectrum of Compound **3k**

^13^ C NMR (126 MHz, CDCl_3_) Spectrum of Compound **3k**

^19^ F NMR (471 MHz, CDCl_3_) Spectrum of Compound **3k**

^1^ H NMR (600 MHz, CDCl_3_) Spectrum of Compound **3l**

^13^ C NMR (151 MHz, CDCl_3_) Spectrum of Compound **3l**

^19^ F NMR (565 MHz, CDCl_3_) Spectrum of Compound **3l**

^1^ H NMR (500 MHz, CDCl_3_) Spectrum of Compound **3m**

^13^ C NMR (126 MHz, CDCl_3_) Spectrum of Compound **3m**

^19^ F NMR (471 MHz, CDCl_3_) Spectrum of Compound **3m**

^1^ H NMR (500 MHz, CDCl_3_) Spectrum of Compound **3n**

^13^ C NMR (126 MHz, CDCl_3_) Spectrum of Compound **3n**

^19^ F NMR (471 MHz, CDCl_3_) Spectrum of Compound **3n**

^1^ H NMR (400 MHz, CDCl_3_) Spectrum of Compound **3o**

^13^ C NMR (101 MHz, CDCl_3_) Spectrum of Compound **3o**

^19^ F NMR (376 MHz, CDCl_3_) Spectrum of Compound **3o**

^1^ H NMR (400 MHz, CDCl_3_) Spectrum of Compound **3p**

^13^ C NMR (101 MHz, CDCl_3_) Spectrum of Compound **3p**

^19^ F NMR (376 MHz, CDCl_3_) Spectrum of Compound **3p**

^1^ H NMR (400 MHz, CDCl_3_) Spectrum of Compound **3q**

^13^ C NMR (101 MHz, CDCl_3_) Spectrum of Compound **3q**

^19^ F NMR (376 MHz, CDCl_3_) Spectrum of Compound **3q**

^1^ H NMR (500 MHz, CDCl_3_) Spectrum of Compound **3r**

^13^ C NMR (126 MHz, CDCl_3_) Spectrum of Compound **3r**

^19^ F NMR (471 MHz, CDCl_3_) Spectrum of Compound **3r**

^1^ H NMR (400 MHz, CDCl_3_) Spectrum of Compound **3s**

^13^ C NMR (101 MHz, CDCl_3_) Spectrum of Compound **3s**

^19^ F NMR (376 MHz, CDCl_3_) Spectrum of Compound **3s**

^1^ H NMR (500 MHz, CDCl_3_) Spectrum of Compound **3t**

^13^ C NMR (126 MHz, CDCl_3_) Spectrum of Compound **3t**

^19^ F NMR (471 MHz, CDCl_3_) Spectrum of Compound **3t**

^1^ H NMR (500 MHz, CDCl_3_) Spectrum of Compound **3u**

^13^ C NMR (126 MHz, CDCl_3_) Spectrum of Compound **3u**

^19^ F NMR (471 MHz, CDCl_3_) Spectrum of Compound **3u**

^1^ H NMR (500 MHz, CDCl_3_) Spectrum of Compound **3v**

^13^ C NMR (126 MHz, CDCl_3_) Spectrum of Compound **3v**

^19^ F NMR (471 MHz, CDCl_3_) Spectrum of Compound **3v**

^1^ H NMR (500 MHz, CDCl_3_) Spectrum of Compound **3w**

^13^ C NMR (126 MHz, CDCl_3_) Spectrum of Compound **3w**

^19^ F NMR (471 MHz, CDCl_3_) Spectrum of Compound **3w**

^1^ H NMR (500 MHz, CDCl_3_) Spectrum of Compound **3x**

^13^ C NMR (126 MHz, CDCl_3_) Spectrum of Compound **3x**

^19^ F NMR (471 MHz, CDCl_3_) Spectrum of Compound **3x**

^1^ H NMR (400 MHz, CDCl_3_) Spectrum of Compound **3y**

^13^ C NMR (101 MHz, CDCl_3_) Spectrum of Compound **3y**

^19^ F NMR (376 MHz, CDCl_3_) Spectrum of Compound **3y**

^1^ H NMR (500 MHz, CDCl_3_) Spectrum of Compound **3z**

^13^ C NMR (126 MHz, CDCl_3_) Spectrum of Compound **3z**

^19^ F NMR (471 MHz, CDCl_3_) Spectrum of Compound **3z**

^1^ H NMR (500 MHz, CDCl_3_) Spectrum of Compound **3aa**

^13^ C NMR (126 MHz, CDCl_3_) Spectrum of Compound **3aa**

^19^ F NMR (471 MHz, CDCl_3_) Spectrum of Compound **3aa**

^1^ H NMR (400 MHz, CDCl_3_) Spectrum of Compound **3ab**

^13^ C NMR (101 MHz, CDCl_3_) Spectrum of Compound **3ab**

^19^ F NMR (376 MHz, CDCl_3_) Spectrum of Compound **3ab**

^1^ H NMR (500 MHz, CDCl_3_) Spectrum of Compound **3ac**

^13^ C NMR (126 MHz, CDCl_3_) Spectrum of Compound **3ac**

^19^ F NMR (471 MHz, CDCl_3_) Spectrum of Compound **3ac**

^1^ H NMR (400 MHz, CDCl_3_) Spectrum of Compound **3ad**

^13^ C NMR (101 MHz, CDCl_3_) Spectrum of Compound **3ad**

^19^ F NMR (376 MHz, CDCl_3_) Spectrum of Compound **3ad**

^1^ H NMR (400 MHz, CDCl_3_) Spectrum of Compound **3ae**

^13^ C NMR (101 MHz, CDCl_3_) Spectrum of Compound **3ae**

^19^ F NMR (376 MHz, CDCl_3_) Spectrum of Compound **3ae**

^1^ H NMR (500 MHz, CDCl_3_) Spectrum of Compound **3af**

^13^ C NMR (126 MHz, CDCl_3_) Spectrum of Compound **3af**

^19^ F NMR (471 MHz, CDCl_3_) Spectrum of Compound **3af**

^1^ H NMR (500 MHz, CDCl_3_) Spectrum of Compound **3ag**

^13^ C NMR (126 MHz, CDCl_3_) Spectrum of Compound **3ag**

^19^ F NMR (471 MHz, CDCl_3_) Spectrum of Compound **3ag**

^1^ H NMR (500 MHz, CDCl_3_) Spectrum of Compound **3ah**

^13^ C NMR (126 MHz, CDCl_3_) Spectrum of Compound **3ah**

^19^ F NMR (471 MHz, CDCl_3_) Spectrum of Compound **3ah**

^1^ H NMR (500 MHz, CDCl_3_) Spectrum of Compound **3ai**

^13^ C NMR (126 MHz, CDCl_3_) Spectrum of Compound **3ai**

^19^ F NMR (471 MHz, CDCl_3_) Spectrum of Compound **3ai**

^1^ H NMR (500 MHz, CDCl_3_) Spectrum of Compound **3aj**

^13^ C NMR (126 MHz, CDCl_3_) Spectrum of Compound **3aj**

^\^

^19^ F NMR (471 MHz, CDCl_3_) Spectrum of Compound **3aj**

^1^ H NMR (500 MHz, CDCl_3_) Spectrum of Compound **3ak**

^13^ C NMR (126 MHz, CDCl_3_) Spectrum of Compound **3ak**

^19^ F NMR (471 MHz, CDCl_3_) Spectrum of Compound **3ak**

^1^ H NMR (500 MHz, CDCl_3_) Spectrum of Compound **5a**

^13^ C NMR (126 MHz, CDCl_3_) Spectrum of Compound **5a**

^19^ F NMR (471 MHz, CDCl_3_) Spectrum of Compound **5a**

^1^ H NMR (500 MHz, CDCl_3_) Spectrum of Compound **5b**

^13^ C NMR (126 MHz, CDCl_3_) Spectrum of Compound **5b**

^19^ F NMR (471 MHz, CDCl_3_) Spectrum of Compound **5b**

^1^ H NMR (500 MHz, DMSO*-d_6_*) Spectrum of Compound **5c**

^13^ C NMR (126 MHz, DMSO*-d_6_*) Spectrum of Compound **5c**

^19^ F NMR (471 MHz, DMSO*-d_6_*) Spectrum of Compound **5c**

^1^ H NMR (500 MHz, DMSO*-d_6_*) Spectrum of Compound **5d**

^13^ C NMR (126 MHz, DMSO*-d_6_*) Spectrum of Compound **5d**

^19^ F NMR (471 MHz, DMSO*-d_6_*) Spectrum of Compound **5d**

^1^ H NMR (500 MHz, DMSO*-d_6_*) Spectrum of Compound **5e**

^13^ C NMR (126 MHz, DMSO*-d_6_*) Spectrum of Compound **5e**

^19^ F NMR (471 MHz, DMSO*-d_6_*) Spectrum of Compound **5e**

^1^ H NMR (400 MHz, CDCl_3_) Spectrum of Compound **5f**

^13^ C NMR (101 MHz, CDCl_3_) Spectrum of Compound **5f**

^19^ F NMR (376 MHz, CDCl_3_) Spectrum of Compound **5f**

^1^ H NMR (600 MHz, CDCl_3_) Spectrum of Compound **5g**

^13^ C NMR (151 MHz, CDCl_3_) Spectrum of Compound **5g**

^19^ F NMR (565 MHz, CDCl_3_) Spectrum of Compound **5g**

^1^ H NMR (600 MHz, CDCl_3_) Spectrum of Compound **5h**

^13^ C NMR (151 MHz, CDCl_3_) Spectrum of Compound **5h**

^19^ F NMR (565 MHz, CDCl_3_) Spectrum of Compound **5h**

^1^ H NMR (600 MHz, CDCl_3_) Spectrum of Compound **5i**

^13^ C NMR (151 MHz, CDCl_3_) Spectrum of Compound **5i**

^19^ F NMR (565 MHz, CDCl_3_) Spectrum of Compound **5i**

^1^ H NMR (500 MHz, CDCl_3_) Spectrum of Compound **5j**

^13^ C NMR (126 MHz, CDCl_3_) Spectrum of Compound **5j**

^19^ F NMR (471 MHz, CDCl_3_) Spectrum of Compound **5j**

^1^ H NMR (500 MHz, CDCl_3_) Spectrum of Compound **5k**

^13^ C NMR (126 MHz, CDCl_3_) Spectrum of Compound **5k**

^19^ F NMR (471 MHz, CDCl_3_) Spectrum of Compound **5k**

^1^ H NMR (500 MHz, CDCl_3_) Spectrum of Compound **5l**

^13^ C NMR (126 MHz, CDCl_3_) Spectrum of Compound **5l**

^19^ F NMR (471 MHz, CDCl_3_) Spectrum of Compound **5l**

^1^ H NMR (500 MHz, CDCl_3_) Spectrum of Compound **5m**

^13^ C NMR (126 MHz, CDCl_3_) Spectrum of Compound **5m**

^19^ F NMR (471 MHz, CDCl_3_) Spectrum of Compound **5m**

^1^ H NMR (500 MHz, DMSO*-d_6_*) Spectrum of Compound **5n**

^13^ C NMR (126 MHz, DMSO*-d_6_*) Spectrum of Compound **5n**

^19^ F NMR (471 MHz, DMSO*-d_6_*) Spectrum of Compound **5n**

^1^ H NMR (500 MHz, DMSO*-d_6_*) Spectrum of Compound **5o**

^13^ C NMR (126 MHz, DMSO*-d_6_*) Spectrum of Compound **5o**

^19^ F NMR (471 MHz, DMSO*-d_6_*) Spectrum of Compound **5o**

^1^ H NMR (500 MHz, DMSO*-d_6_*) Spectrum of Compound **5p**

^13^ C NMR (126 MHz, DMSO*-d_6_*) Spectrum of Compound **5p**

^19^ F NMR (471 MHz, DMSO*-d_6_*) Spectrum of Compound **5p**

^1^ H NMR (500 MHz, DMSO*-d_6_*) Spectrum of Compound **5q**

^13^ C NMR (126 MHz, DMSO*-d_6_*) Spectrum of Compound **5q**

^19^ F NMR (471 MHz, DMSO*-d_6_*) Spectrum of Compound **5q**

^1^ H NMR (500 MHz, DMSO*-d_6_*) Spectrum of Compound **5r**

^13^ C NMR (126 MHz, DMSO*-d_6_*) Spectrum of Compound **5r**

^19^ F NMR (471 MHz, DMSO*-d_6_*) Spectrum of Compound **5r**

^1^ H NMR (400 MHz, CDCl_3_) Spectrum of Compound **5s**

^13^ C NMR (101 MHz, CDCl_3_) Spectrum of Compound **5s**

^19^ F NMR (376 MHz, CDCl_3_) Spectrum of Compound **5s**

^1^ H NMR (500 MHz, DMSO*-d_6_*) Spectrum of Compound **5t**

^13^ C NMR (126 MHz, DMSO*-d_6_*) Spectrum of Compound **5t**

^19^ F NMR (471 MHz, DMSO*-d_6_*) Spectrum of Compound **5t**

^1^ H NMR (500 MHz, DMSO*-d_6_*) Spectrum of Compound **5u**

^13^ C NMR (126 MHz, DMSO*-d_6_*) Spectrum of Compound **5u**

^19^ F NMR (471 MHz, DMSO*-d_6_*) Spectrum of Compound **5u**

^1^ H NMR (400 MHz, CDCl_3_) Spectrum of Compound **5v**

^13^ C NMR (101 MHz, CDCl_3_) Spectrum of Compound **5v**

^19^ F NMR (376 MHz, CDCl_3_) Spectrum of Compound **5v**

^1^ H NMR (500 MHz, DMSO*-d_6_*) Spectrum of Compound **5w**

^13^ C NMR (126 MHz, DMSO*-d_6_*) Spectrum of Compound **5w**

^19^ F NMR (471 MHz, DMSO*-d_6_*) Spectrum of Compound **5w**

^1^ H NMR (400 MHz, CDCl_3_) Spectrum of Compound **6a**

^13^ C NMR (101 MHz, CDCl_3_) Spectrum of Compound **6a**

^19^ F NMR (376 MHz, CDCl_3_) Spectrum of Compound **6a**

^1^ H NMR (400 MHz, CDCl_3_) Spectrum of Compound **6e**

^13^ C NMR (101 MHz, CDCl_3_) Spectrum of Compound **6e**

^19^ F NMR (376 MHz, CDCl_3_) Spectrum of Compound **6e**

^1^ H NMR (500 MHz, DMSO*-d_6_*) Spectrum of Compound **7a**

^13^ C NMR (126 MHz, DMSO*-d_6_*) Spectrum of Compound **7a**

^19^ F NMR (471 MHz, DMSO*-d_6_*) Spectrum of Compound **7a**

HPLC Data of Products

| Peak# | Ret.time | Area | Area% |
| --- | --- | --- | --- |
| 1 | 9.762 | 3122299 | 49.998 |
| 2 | 10.436 | 3122599 | 50.002 |
| Total |  | 6244898 | 100.000 |

| Peak# | Ret.time | Area | Area% |
| --- | --- | --- | --- |
| 1 | 9.698 | 80701 | 0.726 |
| 2 | 10.351 | 11042622 | 99.274 |
| Total |  | 11123323 | 100.000 |

| Peak# | Ret.time | Area | Area% |
| --- | --- | --- | --- |
| 1 | 12.788 | 10885840 | 50.595 |
| 2 | 20.548 | 10629828 | 49.405 |
| Total |  | 21515668 | 100.000 |

| Peak# | Ret.time | Area | Area% |
| --- | --- | --- | --- |
| 1 | 12.843 | 136629 | 0.900 |
| 2 | 20.547 | 15037587 | 99.100 |
| Total |  | 15174216 | 100.000 |

| Peak# | Ret.time | Area | Area% |
| --- | --- | --- | --- |
| 1 | 15.207 | 4245503 | 50.005 |
| 2 | 19.257 | 4244689 | 49.995 |
| Total |  | 8490191 | 100.000 |

| Peak# | Ret.time | Area | Area% |
| --- | --- | --- | --- |
| 1 | 15.291 | 161582 | 0.997 |
| 2 | 19.365 | 16041361 | 99.003 |
| Total |  | 16202943 | 100.000 |

| Peak# | Ret.time | Area | Area% |
| --- | --- | --- | --- |
| 1 | 14.729 | 7277424 | 49.969 |
| 2 | 29.921 | 7286421 | 50.031 |
| Total |  | 14563844 | 100.000 |

| Peak# | Ret.time | Area | Area% |
| --- | --- | --- | --- |
| 1 | 15.045 | 195516 | 0.631 |
| 2 | 29.572 | 30781125 | 99.369 |
| Total |  | 21155647 | 100.000 |

| Peak# | Ret.time | Area | Area% |
| --- | --- | --- | --- |
| 1 | 10.462 | 2699301 | 51.485 |
| 2 | 13.184 | 2543572 | 48.515 |
| Total |  | 5242873 | 100.000 |

| Peak# | Ret.time | Area | Area% |
| --- | --- | --- | --- |
| 1 | 9.963 | 87638 | 0.744 |
| 2 | 13.341 | 11692281 | 99.256 |
| Total |  | 11779919 | 100.000 |

| Peak# | Ret.time | Area | Area% |
| --- | --- | --- | --- |
| 1 | 12.498 | 2211183 | 48.163 |
| 2 | 16.993 | 2379844 | 51.837 |
| Total |  | 4591027 | 100.000 |

| Peak# | Ret.time | Area | Area% |
| --- | --- | --- | --- |
| 1 | 12.635 | 582808 | 1.149 |
| 2 | 17.098 | 50135297 | 98.851 |
| Total |  | 50718104 | 100.000 |

| Peak# | Ret.time | Area | Area% |
| --- | --- | --- | --- |
| 1 | 13.365 | 13760720 | 49.258 |
| 2 | 17.722 | 14175471 | 50.742 |
| Total |  | 27936192 | 100.000 |

| Peak# | Ret.time | Area | Area% |
| --- | --- | --- | --- |
| 1 | 13.471 | 500411 | 0.932 |
| 2 | 17.768 | 53199342 | 99.068 |
| Total |  | 53699753 | 100.000 |

| Peak# | Ret.time | Area | Area% |
| --- | --- | --- | --- |
| 1 | 11.631 | 6704236 | 49.519 |
| 2 | 18.902 | 6834557 | 50.481 |
| Total |  | 13538793 | 100.000 |

| Peak# | Ret.time | Area | Area% |
| --- | --- | --- | --- |
| 1 | 11.759 | 289198 | 0.874 |
| 2 | 18.857 | 32818191 | 99.126 |
| Total |  | 33107389 | 100.000 |

| Peak# | Ret.time | Area | Area% |
| --- | --- | --- | --- |
| 1 | 16.777 | 13850034 | 50.628 |
| 2 | 18.420 | 13506288 | 49.372 |
| Total |  | 27356322 | 100.000 |

| Peak# | Ret.time | Area | Area% |
| --- | --- | --- | --- |
| 1 | 16.859 | 46031 | 0.507 |
| 2 | 18.489 | 9030282 | 99.493 |
| Total |  | 9076313 | 100.000 |

| Peak# | Ret.time | Area | Area% |
| --- | --- | --- | --- |
| 1 | 14.387 | 5844118 | 49.320 |
| 2 | 16.226 | 6005214 | 50.680 |
| Total |  | 11849332 | 100.000 |

| Peak# | Ret.time | Area | Area% |
| --- | --- | --- | --- |
| 1 | 14.455 | 738876 | 1.264 |
| 2 | 16.217 | 57737858 | 98.736 |
| Total |  | 58476734 | 100.000 |

| Peak# | Ret.time | Area | Area% |
| --- | --- | --- | --- |
| 1 | 10.052 | 7140226 | 50.140 |
| 2 | 11.696 | 7100440 | 49.860 |
| Total |  | 14240666 | 100.000 |

| Peak# | Ret.time | Area | Area% |
| --- | --- | --- | --- |
| 1 | 10.031 | 235302 | 2.066 |
| 2 | 11.664 | 11151265 | 97.934 |
| Total |  | 11386567 | 100.000 |

| Peak# | Ret.time | Area | Area% |
| --- | --- | --- | --- |
| 1 | 10.012 | 31264956 | 49.632 |
| 2 | 12.183 | 31723115 | 50.368 |
| Total |  | 62994071 | 100.000 |

| Peak# | Ret.time | Area | Area% |
| --- | --- | --- | --- |
| 1 | 10.102 | 104784 | 0.860 |
| 2 | 12.345 | 12080347 | 99.140 |
| Total |  | 12185131 | 100.000 |

| Peak# | Ret.time | Area | Area% |
| --- | --- | --- | --- |
| 1 | 10.763 | 2739899 | 48.890 |
| 2 | 14.955 | 2864302 | 51.110 |
| Total |  | 5604201 | 100.000 |

| Peak# | Ret.time | Area | Area% |
| --- | --- | --- | --- |
| 1 | 10.782 | 1356971 | 2.560 |
| 2 | 14.984 | 51642657 | 97.440 |
| Total |  | 52999628 | 100.000 |

| Peak# | Ret.time | Area | Area% |
| --- | --- | --- | --- |
| 1 | 20.316 | 987628 | 49.951 |
| 2 | 29.479 | 989553 | 50.049 |
| Total |  | 1977181 | 100.000 |

| Peak# | Ret.time | Area | Area% |
| --- | --- | --- | --- |
| 1 | 20.319 | 136469 | 1.234 |
| 2 | 29.323 | 10922387 | 98.766 |
| Total |  | 11058856 | 100.000 |

| Peak# | Ret.time | Area | Area% |
| --- | --- | --- | --- |
| 1 | 24.120 | 9620477 | 50.720 |
| 2 | 42.362 | 9347392 | 49.280 |
| Total |  | 18967868 | 100.000 |

| Peak# | Ret.time | Area | Area% |
| --- | --- | --- | --- |
| 1 | 24.433 | 331303 | 0.866 |
| 2 | 42.190 | 37934468 | 99.134 |
| Total |  | 38265571 | 100.000 |

| Peak# | Ret.time | Area | Area% |
| --- | --- | --- | --- |
| 1 | 12.403 | 21864070 | 49.800 |
| 2 | 17.461 | 22039554 | 50.200 |
| Total |  | 43903624 | 100.000 |

| Peak# | Ret.time | Area | Area% |
| --- | --- | --- | --- |
| 1 | 12.433 | 1189462 | 1.514 |
| 2 | 17.475 | 77373545 | 98.486 |
| Total |  | 78563007 | 100.000 |

| Peak# | Ret.time | Area | Area% |
| --- | --- | --- | --- |
| 1 | 13.698 | 33386959 | 48.467 |
| 2 | 22.454 | 35499701 | 51.533 |
| Total |  | 68886660 | 100.000 |

| Peak# | Ret.time | Area | Area% |
| --- | --- | --- | --- |
| 1 | 13.839 | 930490 | 1.750 |
| 2 | 22.414 | 52239936 | 98.250 |
| Total |  | 53170425 | 100.000 |

| Peak# | Ret.time | Area | Area% |
| --- | --- | --- | --- |
| 1 | 12.461 | 4903438 | 50.524 |
| 2 | 14.840 | 4801807 | 49.476 |
| Total |  | 9705245 | 100.000 |

| Peak# | Ret.time | Area | Area% |
| --- | --- | --- | --- |
| 1 | 12.480 | 1759296 | 4.733 |
| 2 | 14..650 | 35410779 | 95.267 |
| Total |  | 37170076 | 100.000 |

| Peak# | Ret.time | Area | Area% |
| --- | --- | --- | --- |
| 1 | 9.291 | 12669539 | 50.794 |
| 2 | 10.247 | 12273593 | 49.206 |
| Total |  | 24943133 | 100.000 |

| Peak# | Ret.time | Area | Area% |
| --- | --- | --- | --- |
| 1 | 9.330 | 414323 | 2.067 |
| 2 | 10.256 | 19631434 | 97.933 |
| Total |  | 20045757 | 100.000 |

| Peak# | Ret.time | Area | Area% |
| --- | --- | --- | --- |
| 1 | 10.405 | 806451 | 49.789 |
| 2 | 12.037 | 813286 | 50.211 |
| Total |  | 1619737 | 100.000 |

| Peak# | Ret.time | Area | Area% |
| --- | --- | --- | --- |
| 1 | 10.256 | 1211936 | 2.682 |
| 2 | 11.859 | 43983527 | 97.318 |
| Total |  | 45195463 | 100.000 |

| Peak# | Ret.time | Area | Area% |
| --- | --- | --- | --- |
| 1 | 12.179 | 17625425 | 50.632 |
| 2 | 13.984 | 17185445 | 49.368 |
| Total |  | 34810870 | 100.000 |

| Peak# | Ret.time | Area | Area% |
| --- | --- | --- | --- |
| 1 | 12.203 | 324902 | 1.364 |
| 2 | 13.996 | 23500031 | 98.636 |
| Total |  | 23824933 | 100.000 |

| Peak# | Ret.time | Area | Area% |
| --- | --- | --- | --- |
| 1 | 11.206 | 8869995 | 49.112 |
| 2 | 13.715 | 9190862 | 50.888 |
| Total |  | 18060857 | 100.000 |

| Peak# | Ret.time | Area | Area% |
| --- | --- | --- | --- |
| 1 | 10.592 | 74030 | 1.599 |
| 2 | 13.708 | 4557158 | 98.401 |
| Total |  | 4631188 | 100.000 |

| Peak# | Ret.time | Area | Area% |
| --- | --- | --- | --- |
| 1 | 9.100 | 7153174 | 48.828 |
| 2 | 10.148 | 7496439 | 51.172 |
| Total |  | 14649613 | 100.000 |

| Peak# | Ret.time | Area | Area% |
| --- | --- | --- | --- |
| 1 | 8.935 | 102482 | 0.728 |
| 2 | 9.974 | 13970601 | 99.272 |
| Total |  | 14073084 | 100.000 |

| Peak# | Ret.time | Area | Area% |
| --- | --- | --- | --- |
| 1 | 27.926 | 4011226 | 50.060 |
| 2 | 44.881 | 4001683 | 49.940 |
| Total |  | 8012909 | 100.000 |

| Peak# | Ret.time | Area | Area% |
| --- | --- | --- | --- |
| 1 | 28.125 | 387281 | 1.351 |
| 2 | 44.746 | 28282200 | 98.649 |
| Total |  | 28669482 | 100.000 |

| Peak# | Ret.time | Area | Area% |
| --- | --- | --- | --- |
| 1 | 11.750 | 4866065 | 50.965 |
| 2 | 15.469 | 4681877 | 49.035 |
| Total |  | 9547934 | 100.000 |

| Peak# | Ret.time | Area | Area% |
| --- | --- | --- | --- |
| 1 | 12.183 | 555609 | 1.836 |
| 2 | 15.587 | 29698270 | 98.164 |
| Total |  | 28915755 | 100.000 |

| Peak# | Ret.time | Area | Area% |
| --- | --- | --- | --- |
| 1 | 25.147 | 13454508 | 51.048 |
| 2 | 31.802 | 12901859 | 48.952 |
| Total |  | 26356366 | 100.000 |

| Peak# | Ret.time | Area | Area% |
| --- | --- | --- | --- |
| 1 | 24.809 | 87808 | 0.573 |
| 2 | 31.406 | 15231180 | 99.427 |
| Total |  | 15318988 | 100.000 |

| Peak# | Ret.time | Area | Area% |
| --- | --- | --- | --- |
| 1 | 10.803 | 3825265 | 50.087 |
| 2 | 12.551 | 3811916 | 49.913 |
| Total |  | 7637182 | 100.000 |

| Peak# | Ret.time | Area | Area% |
| --- | --- | --- | --- |
| 1 | 10.972 | 241097 | 0.765 |
| 2 | 12.730 | 31267107 | 99.235 |
| Total |  | 31508204 | 100.000 |

| Peak# | Ret.time | Area | Area% |
| --- | --- | --- | --- |
| 1 | 8.870 | 2806470 | 50.190 |
| 2 | 10.987 | 2785263 | 49.810 |
| Total |  | 5591733 | 100.000 |

| Peak# | Ret.time | Area | Area% |
| --- | --- | --- | --- |
| 1 | 8.881 | 931512 | 2.146 |
| 2 | 10.900 | 42466809 | 97.854 |
| Total |  | 43398321 | 100.000 |

| Peak# | Ret.time | Area | Area% |
| --- | --- | --- | --- |
| 1 | 14.740 | 3705680 | 50.490 |
| 2 | 24.010 | 3633812 | 49.510 |
| Total |  | 7339493 | 100.000 |

| Peak# | Ret.time | Area | Area% |
| --- | --- | --- | --- |
| 1 | 14.984 | 228273 | 3.533 |
| 2 | 24.014 | 6232015 | 96.467 |
| Total |  | 6460288 | 100.000 |

| Peak# | Ret.time | Area | Area% |
| --- | --- | --- | --- |
| 1 | 11.357 | 2081454 | 50.025 |
| 2 | 13.758 | 2079338 | 49.975 |
| Total |  | 4160792 | 100.000 |

| Peak# | Ret.time | Area | Area% |
| --- | --- | --- | --- |
| 1 | 11.326 | 185270 | 4.932 |
| 2 | 13.717 | 3571150 | 95.068 |
| Total |  | 3756419 | 100.000 |

| Peak# | Ret.time | Area | Area% |
| --- | --- | --- | --- |
| 1 | 21.427 | 2049574 | 50.464 |
| 2 | 25.486 | 2011869 | 49.536 |
| Total |  | 4061443 | 100.000 |

| Peak# | Ret.time | Area | Area% |
| --- | --- | --- | --- |
| 1 | 22.258 | 1892282 | 6.626 |
| 2 | 26.231 | 26664573 | 93.374 |
| Total |  | 28556854 | 100.000 |

| Peak# | Ret.time | Area | Area% |
| --- | --- | --- | --- |
| 1 | 65.385 | 13435109 | 50.981 |
| 2 | 79.421 | 12918067 | 49.019 |
| Total |  | 26353176 | 100.000 |

| Peak# | Ret.time | Area | Area% |
| --- | --- | --- | --- |
| 1 | 62.282 | 99265 | 2.636 |
| 2 | 76.430 | 3666126 | 97.364 |
| Total |  | 3765391 | 100.000 |

| Peak# | Ret.time | Area | Area% |
| --- | --- | --- | --- |
| 1 | 23.414 | 14233826 | 50.626 |
| 2 | 37.635 | 13881574 | 49.374 |
| Total |  | 28115400 | 100.000 |

| Peak# | Ret.time | Area | Area% |
| --- | --- | --- | --- |
| 1 | 22.685 | 1076328 | 1.175 |
| 2 | 35.801 | 90489852 | 98.825 |
| Total |  | 91566180 | 100.000 |

| Peak# | Ret.time | Area | Area% |
| --- | --- | --- | --- |
| 1 | 27.209 | 11947212 | 50.778 |
| 2 | 39.189 | 11580948 | 49.222 |
| Total |  | 23528160 | 100.000 |

| Peak# | Ret.time | Area | Area% |
| --- | --- | --- | --- |
| 1 | 29.880 | 86097 | 0.338 |
| 2 | 42.179 | 25419490 | 99.662 |
| Total |  | 25505587 | 100.000 |

| Peak# | Ret.time | Area | Area% |
| --- | --- | --- | --- |
| 1 | 11.782 | 45031753 | 50.018 |
| 2 | 15.718 | 45000121 | 49.982 |
| Total |  | 90031874 | 100.000 |

| Peak# | Ret.time | Area | Area% |
| --- | --- | --- | --- |
| 1 | 11.752 | 376381 | 1.383 |
| 2 | 15.685 | 26836304 | 98.617 |
| Total |  | 27212685 | 100.000 |

| Peak# | Ret.time | Area | Area% |
| --- | --- | --- | --- |
| 1 | 72.861 | 836622 | 51.188 |
| 2 | 77.559 | 797793 | 48.812 |
| Total |  | 1634415 | 100.000 |

| Peak# | Ret.time | Area | Area% |
| --- | --- | --- | --- |
| 1 | 74.212 | 95625 | 1.149 |
| 2 | 78.479 | 8229228 | 98.851 |
| Total |  | 8324853 | 100.000 |

| Peak# | Ret.time | Area | Area% |
| --- | --- | --- | --- |
| 1 | 22.256 | 7285075 | 50.003 |
| 2 | 31.294 | 7284267 | 49.997 |
| Total |  | 14569342 | 100.000 |

| Peak# | Ret.time | Area | Area% |
| --- | --- | --- | --- |
| 1 | 21.956 | 94294 | 0.859 |
| 2 | 30.776 | 10888516 | 99.141 |
| Total |  | 10982810 | 100.000 |

| Peak# | Ret.time | Area | Area% |
| --- | --- | --- | --- |
| 1 | 38.659 | 17034093 | 50.265 |
| 2 | 42.160 | 16854716 | 49.735 |
| Total |  | 33888809 | 100.000 |

| Peak# | Ret.time | Area | Area% |
| --- | --- | --- | --- |
| 1 | 38.527 | 29089631 | 96.308 |
| 2 | 42.339 | 1115019 | 3.692 |
| Total |  | 30204650 | 100.000 |

| Peak# | Ret.time | Area | Area% |
| --- | --- | --- | --- |
| 1 | 29.107 | 4150377 | 50.884 |
| 2 | 65.618 | 4006099 | 49.116 |
| Total |  | 8156476 | 100.000 |

| Peak# | Ret.time | Area | Area% |
| --- | --- | --- | --- |
| 1 | 29.282 | 1823156 | 4.693 |
| 2 | 66.350 | 37021321 | 95.307 |
| Total |  | 38844476 | 100.000 |

| Peak# | Ret.time | Area | Area% |
| --- | --- | --- | --- |
| 1 | 52.315 | 5780988 | 49.313 |
| 2 | 60.938 | 5942148 | 50.687 |
| Total |  | 11723136 | 100.000 |

| Peak# | Ret.time | Area | Area% |
| --- | --- | --- | --- |
| 1 | 52.657 | 16228900 | 95.613 |
| 2 | 62.283 | 744696 | 4.387 |
| Total |  | 16973596 | 100.000 |

| Peak# | Ret.time | Area | Area% |
| --- | --- | --- | --- |
| 1 | 18.950 | 1157664 | 49.654 |
| 2 | 30.818 | 1173801 | 50.346 |
| Total |  | 2331465 | 100.000 |

| Peak# | Ret.time | Area | Area% |
| --- | --- | --- | --- |
| 1 | 20.286 | 221498 | 4.302 |
| 2 | 32.278 | 4927683 | 95.698 |
| Total |  | 5149181 | 100.000 |

| Peak# | Ret.time | Area | Area% |
| --- | --- | --- | --- |
| 1 | 24.763 | 3561221 | 50.272 |
| 2 | 27.051 | 3522691 | 49.728 |
| Total |  | 7083912 | 100.000 |

| Peak# | Ret.time | Area | Area% |
| --- | --- | --- | --- |
| 1 | 24.713 | 801645 | 7.214 |
| 2 | 26.943 | 10310034 | 92.786 |
| Total |  | 11111679 | 100.000 |

| Peak# | Ret.time | Area | Area% |
| --- | --- | --- | --- |
| 1 | 20.320 | 1213991 | 50.488 |
| 2 | 27.555 | 1190510 | 49.512 |
| Total |  | 2404501 | 100.000 |

| Peak# | Ret.time | Area | Area% |
| --- | --- | --- | --- |
| 1 | 20.214 | 1512185 | 7.100 |
| 2 | 27.014 | 19787572 | 92.900 |
| Total |  | 21299756 | 100.000 |

| Peak# | Ret.time | Area | Area% |
| --- | --- | --- | --- |
| 1 | 32.216 | 2654431 | 50.914 |
| 2 | 34.844 | 2559177 | 49.086 |
| Total |  | 5213608 | 100.000 |

| Peak# | Ret.time | Area | Area% |
| --- | --- | --- | --- |
| 1 | 33.402 | 16819148 | 95.018 |
| 2 | 36.247 | 881918 | 4.982 |
| Total |  | 17701065 | 100.000 |

| Peak# | Ret.time | Area | Area% |
| --- | --- | --- | --- |
| 1 | 25.529 | 5040907 | 50.575 |
| 2 | 32.699 | 4926204 | 49.425 |
| Total |  | 9967112 | 100.000 |

| Peak# | Ret.time | Area | Area% |
| --- | --- | --- | --- |
| 1 | 25.474 | 53913805 | 94.898 |
| 2 | 32.822 | 2898698 | 5.102 |
| Total |  | 56812503 | 100.000 |

| Peak# | Ret.time | Area | Area% |
| --- | --- | --- | --- |
| 1 | 34.995 | 9803383 | 50.582 |
| 2 | 58.718 | 9577813 | 49.418 |
| Total |  | 19381196 | 100.000 |

| Peak# | Ret.time | Area | Area% |
| --- | --- | --- | --- |
| 1 | 35.262 | 64778131 | 96.829 |
| 2 | 59.578 | 2898698 | 3.171 |
| Total |  | 66899326 | 100.000 |

| Peak# | Ret.time | Area | Area% |
| --- | --- | --- | --- |
| 1 | 32.882 | 1785691 | 50.597 |
| 2 | 35.393 | 1743576 | 49.403 |
| Total |  | 3529266 | 100.000 |

| Peak# | Ret.time | Area | Area% |
| --- | --- | --- | --- |
| 1 | 32.109 | 17345114 | 97.128 |
| 2 | 34.593 | 512834 | 2.872 |
| Total |  | 17857949 | 100.000 |

| Peak# | Ret.time | Area | Area% |
| --- | --- | --- | --- |
| 1 | 64.978 | 2823693 | 49.747 |
| 2 | 69.036 | 2852380 | 50.253 |
| Total |  | 5676074 | 100.000 |

| Peak# | Ret.time | Area | Area% |
| --- | --- | --- | --- |
| 1 | 65.811 | 6842525 | 95.694 |
| 2 | 70.837 | 307874 | 4.306 |
| Total |  | 7150399 | 100.000 |

| Peak# | Ret.time | Area | Area% |
| --- | --- | --- | --- |
| 1 | 26.373 | 2792820 | 51.201 |
| 2 | 32.849 | 2661812 | 48.799 |
| Total |  | 5454632 | 100.000 |

| Peak# | Ret.time | Area | Area% |
| --- | --- | --- | --- |
| 1 | 27.010 | 794643 | 4.476 |
| 2 | 33.497 | 16957080 | 95.524 |
| Total |  | 17751723 | 100.000 |

| Peak# | Ret.time | Area | Area% |
| --- | --- | --- | --- |
| 1 | 65.406 | 924676 | 50.290 |
| 2 | 70.814 | 913994 | 49.710 |
| Total |  | 1838670 | 100.000 |

| Peak# | Ret.time | Area | Area% |
| --- | --- | --- | --- |
| 1 | 65.301 | 908288 | 2.958 |
| 2 | 70.078 | 29794475 | 97.042 |
| Total |  | 30702763 | 100.000 |

| Peak# | Ret.time | Area | Area% |
| --- | --- | --- | --- |
| 1 | 59.669 | 1961157 | 48.814 |
| 2 | 77.477 | 2056472 | 51.186 |
| Total |  | 4017628 | 100.000 |

| Peak# | Ret.time | Area | Area% |
| --- | --- | --- | --- |
| 1 | 60.616 | 811300 | 2.478 |
| 2 | 77.493 | 31925945 | 97.552 |
| Total |  | 32737245 | 100.000 |

| Peak# | Ret.time | Area | Area% |
| --- | --- | --- | --- |
| 1 | 66.831 | 3635479 | 48.042 |
| 2 | 80.946 | 3931865 | 51.958 |
| Total |  | 7567344 | 100.000 |

| Peak# | Ret.time | Area | Area% |
| --- | --- | --- | --- |
| 1 | 67.177 | 1177796 | 3.199 |
| 2 | 81.434 | 35634559 | 96.801 |
| Total |  | 36812356 | 100.000 |

| Peak# | Ret.time | Area | Area% |
| --- | --- | --- | --- |
| 1 | 55.760 | 937754 | 49.243 |
| 2 | 65.393 | 966599 | 50.757 |
| Total |  | 1904353 | 100.000 |

| Peak# | Ret.time | Area | Area% |
| --- | --- | --- | --- |
| 1 | 55.045 | 1108056 | 2.735 |
| 2 | 67.203 | 39411256 | 97.265 |
| Total |  | 40519312 | 100.000 |

| Peak# | Ret.time | Area | Area% |
| --- | --- | --- | --- |
| 1 | 81.863 | 3939823 | 49.212 |
| 2 | 86.664 | 4066023 | 50.788 |
| Total |  | 8005846 | 100.000 |

| Peak# | Ret.time | Area | Area% |
| --- | --- | --- | --- |
| 1 | 79.867 | 29842991 | 95.278 |
| 2 | 85.007 | 1479170 | 4.722 |
| Total |  | 31322161 | 100.000 |

| Peak# | Ret.time | Area | Area% |
| --- | --- | --- | --- |
| 1 | 64.867 | 1783728 | 51.192 |
| 2 | 68.875 | 1700645 | 48.808 |
| Total |  | 3484373 | 100.000 |

| Peak# | Ret.time | Area | Area% |
| --- | --- | --- | --- |
| 1 | 65.989 | 8887783 | 94.633 |
| 2 | 70.332 | 504052 | 5.367 |
| Total |  | 9391835 | 100.000 |

| Peak# | Ret.time | Area | Area% |
| --- | --- | --- | --- |
| 1 | 50.938 | 2320152 | 50.095 |
| 2 | 56.943 | 2311351 | 49.905 |
| Total |  | 4631503 | 100.000 |

| Peak# | Ret.time | Area | Area% |
| --- | --- | --- | --- |
| 1 | 51.358 | 936946 | 4.842 |
| 2 | 56.542 | 18411674 | 95.158 |
| Total |  | 19348620 | 100.000 |

| Peak# | Ret.time | Area | Area% |
| --- | --- | --- | --- |
| 1 | 28.340 | 9303389 | 50.533 |
| 2 | 37.489 | 9107097 | 49.467 |
| Total |  | 18410486 | 100.000 |

| Peak# | Ret.time | Area | Area% |
| --- | --- | --- | --- |
| 1 | 28.098 | 56804241 | 95.398 |
| 2 | 37.704 | 2740138 | 4.602 |
| Total |  | 59544379 | 100.000 |

| Peak# | Ret.time | Area | Area% |
| --- | --- | --- | --- |
| 1 | 33.577 | 328155 | 50.740 |
| 2 | 49.013 | 318583 | 49.260 |
| Total |  | 646738 | 100.000 |

| Peak# | Ret.time | Area | Area% |
| --- | --- | --- | --- |
| 1 | 32.955 | 6313090 | 94.918 |
| 2 | 47.721 | 337985 | 5.082 |
| Total |  | 6651075 | 100.000 |

| Peak# | Ret.time | Area | Area% |
| --- | --- | --- | --- |
| 1 | 31.967 | 3204378 | 49.440 |
| 2 | 39.366 | 3277031 | 50.560 |
| Total |  | 6481409 | 100.000 |

| Peak# | Ret.time | Area | Area% |
| --- | --- | --- | --- |
| 1 | 32.317 | 4552367 | 94.732 |
| 2 | 40.296 | 253154 | 5.268 |
| Total |  | 4805521 | 100.000 |

| Peak# | Ret.time | Area | Area% |
| --- | --- | --- | --- |
| 1 | 30.188 | 590521 | 49.824 |
| 2 | 41.343 | 594700 | 50.176 |
| Total |  | 1185221 | 100.000 |

| Peak# | Ret.time | Area | Area% |
| --- | --- | --- | --- |
| 1 | 30.531 | 1345974 | 95.437 |
| 2 | 41.698 | 64347 | 4.563 |
| Total |  | 1410320 | 100.000 |

| Peak# | Ret.time | Area | Area% |
| --- | --- | --- | --- |
| 1 | 28.714 | 4567671 | 48.943 |
| 2 | 34.848 | 4764917 | 51.057 |
| Total |  | 9332589 | 100.000 |

| Peak# | Ret.time | Area | Area% |
| --- | --- | --- | --- |
| 1 | 28.740 | 17465105 | 98.298 |
| 2 | 35.531 | 302338 | 1.702 |
| Total |  | 17767442 | 100.000 |

| Peak# | Ret.time | Area | Area% |
| --- | --- | --- | --- |
| 1 | 37.475 | 5625424 | 50.507 |
| 2 | 82.985 | 5512392 | 49.493 |
| Total |  | 11137817 | 100.000 |

| Peak# | Ret.time | Area | Area% |
| --- | --- | --- | --- |
| 1 | 37.617 | 426956 | 4.364 |
| 2 | 82.623 | 9356104 | 95.636 |
| Total |  | 9783060 | 100.000 |

| Peak# | Ret.time | Area | Area% |
| --- | --- | --- | --- |
| 1 | 13.027 | 6100260 | 51.269 |
| 2 | 14.135 | 5798290 | 48.731 |
| Total |  | 11898550 | 100.000 |

| Peak# | Ret.time | Area | Area% |
| --- | --- | --- | --- |
| 1 | 12.774 | 22884931 | 99.318 |
| 2 | 13.892 | 157100 | 0.682 |
| Total |  | 23042031 | 100.000 |

Unsuccessful examples

We propose that electronic and/or steric effects are likely the primary contributing factors. A plausible explanation is that aliphatic and sterically hindered vinyl iodides undergo oxidative addition with the nickel species at a slower rate compared to their aryl-conjugated analogues.

X-ray Crystallographic Data of 3d and 5a

| Identification code | LP-5-44-1_auto |
| --- | --- |
| Empirical formula | C_18_H_14_F_2_N_2_O |
| Formula weight | 312.31 |
| Temperature/K | 293(2) |
| Crystal system | triclinic |
| Space group | P1 |
| a/Å | 4.9328(2) |
| b/Å | 12.1049(7) |
| c/Å | 13.7737(7) |
| α/° | 75.535(5) |
| β/° | 87.756(4) |
| γ/° | 89.747(4) |
| Volume/Å^3^ | 795.76(7) |
| Z | 2 |
| ρ_calc_g/cm^3^ | 1.303 |
| μ/mm^‑1^ | 0.823 |
| F(000) | 324.0 |
| Crystal size/mm^3^ | 0.22 × 0.21 × 0.18 |
| Radiation | Cu Kα (λ = 1.54184) |
| 2Θ range for data collection/° | 7.542 to 146.108 |
| Index ranges | -5 ≤ h ≤ 5, -14 ≤ k ≤ 14, -16 ≤ l ≤ 17 |
| Reflections collected | 11076 |
| Independent reflections | 5630 [R_int_ = 0.0242, R_sigma_ = 0.0304] |
| Data/restraints/parameters | 5630/4/415 |
| Goodness-of-fit on F^2^ | 1.060 |
| Final R indexes [I>=2σ (I)] | R_1_ = 0.0712, wR_2_ = 0.2028 |
| Final R indexes [all data] | R_1_ = 0.0861, wR_2_ = 0.2223 |
| Largest diff. peak/hole / e Å^-3^ | 0.77/-0.21 |
| Flack parameter | -0.08(10) |

| Identification code | lp-5-107-1_auto |
| --- | --- |
| Empirical formula | C19H16F3NO3 |
| Formula weight | 363.33 |
| Temperature/K | 293(2) |
| Crystal system | monoclinic |
| Space group | P21 |
| a/Å | 5.07225(13) |
| b/Å | 9.9005(2) |
| c/Å | 17.3012(4) |
| α/° | 90 |
| β/° | 98.217(2) |
| γ/° | 90 |
| Volume/Å3 | 859.91(4) |
| Z | 2 |
| ρcalcg/cm3 | 1.403 |
| μ/mm‑1 | 1.000 |
| F(000) | 376.0 |
| Crystal size/mm3 | 0.18 × 0.17 × 0.17 |
| Radiation | CuKα (λ = 1.54184) |
| 2Θ range for data collection/° | 10.32 to 145.854 |
| Index ranges | -6 ≤ h ≤ 5, -12 ≤ k ≤ 12, -21 ≤ l ≤ 21 |
| Reflections collected | 12091 |
| Independent reflections | 3345 [Rint = 0.0287, Rsigma = 0.0186] |
| Data/restraints/parameters | 3345/1/236 |
| Goodness-of-fit on F2 | 1.055 |
| Final R indexes [I>=2σ (I)] | R1 = 0.0377, wR2 = 0.0995 |
| Final R indexes [all data] | R1 = 0.0401, wR2 = 0.1028 |
| Largest diff. peak/hole / e Å-3 | 0.14/-0.17 |
| Flack parameter | -0.06(6) |

References

[1] Jin, R. X.; Wu, B. B.; Bian, K. J.; Yu, J. L.; Dai, J. C.; Zuo, Y. W.; Zhang, Y. F.; Wang, X. S.; *Nature Communications*, **2022**, *13*, 7035.

[2] Philip, B.; Elliott H. D.; Joel W.; Bill M.; *J. Org. Chem. Soc*. **2023**, *88,* 5069-5077.

[3] Friedrich W.; Wolfgang S.; Zstvan L.; Ferdinand.; Walter O.; *Chemische Berichte*, **1966**, *99*, 1944-1956.

[4] Chen, Y. Z.; Lin, D. K.; Yu, Z. J.; Dong, Z.; Song, L. L.; Chen, L. A.; *Asian J. Org. Chem*. **2023**, *12*, e202300352.

[5] Frisch, M. J.; Trucks, G. W.; Schlegel, H. B.; Scuseria, G. E.; Robb, M. A.; Cheeseman, J. R.; Scalmani, G.; Barone, V.; Petersson, G. A.; Nakatsuji, H.; Li, X.; Caricato, M.; Marenich, A. V.; Bloino, J.; Janesko, B. G.; Gomperts, R.; Mennucci, B.; Hratchian, H. P.; Ortiz, J. V.; Izmaylov, A. F.; Sonnenberg, J. L.; Williams-Young, D.; Ding, F.; Lipparini, F.; Egidi, F.; Goings, J.; Peng, B.; Petrone, A.; Henderson, T.; Ranasinghe, D.; Zakrzewski, V. G.; Gao, J.; Rega, N.; Zheng, G.; Liang, W.; Hada, M.; Ehara, M.; Toyota, K.; Fukuda, R.; Hasegawa, J.; Ishida, M.; Nakajima, T.; Honda, Y.; Kitao, O.; Nakai, H.; Vreven, T.; Throssell, K.; Jr. Montgomery, J. A.; Peralta, J. E.; Ogliaro, F.; Bearpark, M. J.; Heyd, J. J.; Brothers, E. N.; Kudin, K. N.; Staroverov, V. N.; Keith, T. A.; Kobayashi, R.; Normand, J.; Raghavachari, K.; Rendell, A. P.; Burant, J. C.; Iyengar, S. S.; Tomasi, J.; Cossi, M.; Millam, J. M.; Klene, M.; Adamo, C.; Cammi, R.; Ochterski, J. W.; Martin, R. L.; Morokuma, K.; Farkas, O.; Foresman, J. B.; Fox, D. J. *Gaussian 16, Revision C.01, Gaussian, Inc., Wallingford CT*, **2016**.

[6] (a) Becke, A. D. J. Chem. Phys. 1993, 98, 5648. (b) Lee, C. T.; Yang, W. T.; Parr, R. G. *Phys. Rev. B* **1988**, *37*, 785.

[7] Andrae, D.; Häußermann, U.; Dolg, M.; Stoll, H.; Preuß, H. *Theor. Chim. Acta* **1990,** *77*, 123.

[8] Hehre, W. J.; Radom, L.; Schleyer, P. v. R.; Pople. J. A, Ab Initio Molecular Orbital Theory; Wiley: New York, **1986**.

[9] (a) Fukui, K. *Acc. Chem. Res*. **1981**, *14*, 363. (b) Fukui, K. *J. Phys. Chem.*, **1970**, *74*, 4161.

[10] CYLview20; Legault, C. Y., Université de Sherbrooke, **2020** (http://www.cylview.org)
